# High-capacity adenovector delivery of forced CRISPR-Cas9 heterodimers fosters precise chromosomal deletions in human cells

**DOI:** 10.1016/j.omtn.2023.02.025

**Published:** 2023-02-22

**Authors:** Francesca Tasca, Marcella Brescia, Jin Liu, Josephine M. Janssen, Kamel Mamchaoui, Manuel A.F.V. Gonçalves

**Affiliations:** 1 Department of Cell and Chemical Biology, Leiden University Medical Center, Einthovenweg 20, 2333 ZC Leiden, the Netherlands; 2 Sorbonne Université, Inserm, Institut de Myologie, Centre de Recherche en Myologie, Paris, France

**Keywords:** MT: RNA/DNA Editing, multiplexing gene editing, gene delivery, fusion Cas9 proteins, high-capacity adenoviral vectors, on-target specificity, precision gene editing, Duchenne muscular dystrophy, dystrophin

## Abstract

Genome editing based on dual CRISPR-Cas9 complexes (multiplexes) permits removing specific genomic sequences in living cells leveraging research on functional genomics and genetic therapies. Delivering the required large and multicomponent reagents in a synchronous and stoichiometric manner remains, however, challenging. Moreover, uncoordinated activity of independently acting CRISPR-Cas9 multiplexes increases the complexity of genome editing outcomes. Here, we investigate the potential of fostering precise multiplexing genome editing using high-capacity adenovector particles (AdVPs) for the delivery of Cas9 ortholog fusion constructs alone (forced Cas9 heterodimers) or together with their cognate guide RNAs (forced CRISPR-Cas9 heterodimers). We demonstrate that the efficiency and accuracy of targeted chromosomal DNA deletions achieved by single AdVPs encoding forced CRISPR-Cas9 heterodimers is superior to that obtained when the various components are delivered separately. Finally, all-in-one AdVP delivery of forced CRISPR-Cas9 heterodimers triggers robust *DMD* exon 51 splice site excision resulting in reading frame restoration and selection-free detection of dystrophin in muscle cells derived from Duchenne muscular dystrophy patients. In conclusion, AdVPs promote precise multiplexing genome editing through the integrated delivery of forced CRISPR-Cas9 heterodimer components, which, in comparison with split conventional CRISPR-Cas9 multiplexes, engage target sequences in a more coordinated fashion.

## Graphical abstract

**Figure:**
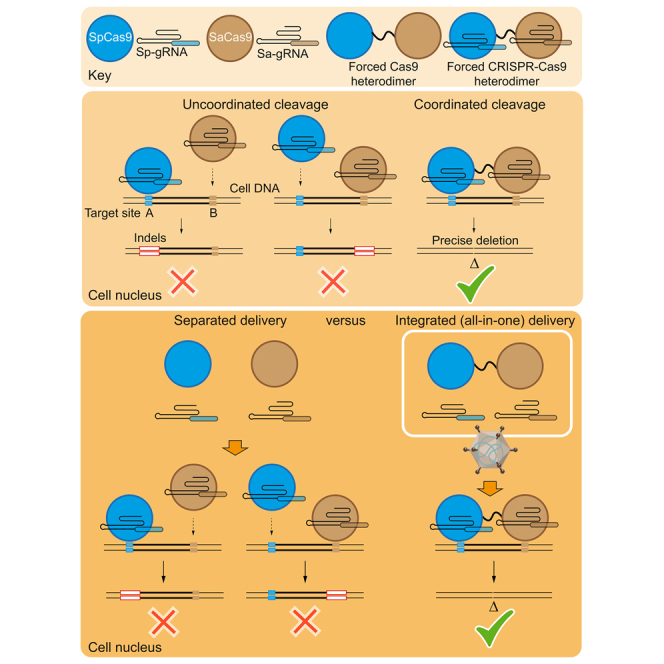

## Teaser

Tasca and colleagues demonstrate the feasibility in introducing into hard-to-transfect cells large CRISPR-Cas9 fusion constructs by packaging them in adenoviral capsids and show that the performance of multiplexing genome editing can profit from the integrated delivery and coordinated assembly of the attendant multiple reagents in target cells.

## Introduction

Diverse types of genetic modifications spanning from single base pairs to mega-bases have been identified as disease-causing genotypes. During the past decade, genetic therapies based on prokaryotic type II clustered regularly interspaced short palindromic repeat (CRISPR)-associated Cas9 (CRISPR-Cas9) systems have started to be investigated and tested for the correction or complementation of such genotypes.^1^^,^^2^ Engineered CRISPR-Cas9 nucleases are ribonucleoprotein complexes consisting of a Cas9 endonuclease and a single guide RNA (gRNA).^3^ The Cas9 protein cleaves target DNA upon recognition of a protospacer adjacent motif (PAM) located next to ∼20-nucleotide tracts complementary to the 5′ end of the gRNA (protospacer). PAM nucleotide sequences differ from one CRISPR-Cas9 system to another. For instance, the PAM of the prototypic and most used *Streptococcus pyogenes* Cas9 (SpCas9) nuclease is NGG.^4^ Its ortholog Cas9 nuclease from *Staphylococcus aureus* (SaCas9) recognizes instead the longer PAM consensus sequence NNGRRT (R = A or G).^5^ The generation of a site-specific double-strand DNA break (DSB) by engineered Cas9:gRNA complexes elicits endogenous DNA repair pathways that can be exploited for targeted genetic modifications.^3^ In mammalian cells, a prevalent DNA repair pathway that arises in response to a DSB is the classical non-homologous end joining (NHEJ).^6^ This pathway results in end-to-end ligation of DSBs that, when inaccurate, yields small insertions and deletions (indels) that can lead to targeted gene knockouts.^3^ In contrast to homology-directed DSB repair, the prevalence of NHEJ throughout the cell cycle makes its exploitation for genome editing purposes possible in both dividing and post-mitotic cells or tissues.^6^

Delivering dual CRISPR-Cas9 complexes formed by a Cas9 nuclease, and two gRNAs (multiplexes) addressed to neighboring target sites, induces NHEJ-mediated intrachromosomal deletions encompassing the sequences located between the site-specific DSBs.^7^^,^^8^^,^^9^ Of notice, this multiplexing genome editing principle has been explored in the first *in vivo* CRISPR-based gene editing clinical trial (BRILLIANCE Phase 1/2). In this trial, patients suffering from Leber congenital amaurosis 10, a severe retinal dystrophy caused by a cryptic exon in *CEP290*, received sub-retinal injections of an advanced medicinal therapy product consisting of a pair of adeno-associated viral (AAV) vectors (EDIT-101). Together, these vectors express *Staphylococcus aureus* CRISPR-Cas9 multiplexes designed for *CEP290* reading frame restoration through NHEJ-mediated excision of the disease-causing cryptic exon.^10^ However, a general consideration regarding multiplexing genome editing concerns the fact that, next to the intended chromosomal deletions, complex genomic modifications normally emerge with much higher frequencies.^8^^,^^9^^,^^11^ The uncoordinated action of the individual CRISPR-Cas9 components contributes to these unintended genome editing endpoints (Figure S1). Unintended bystander products mostly comprise indels at either or both target sites and imprecise deletions in which indels locate at the junction of end-to-end chromosomal termini ligations.^8^^,^^9^^,^^11^ Co-transfection of plasmid constructs encoding dual gRNAs and covalently linked Cas9 nucleases has been shown to heighten the accuracy of targeted DNA deletions following tandem DSB formation.^12^ In addition, owing to orthogonal gRNA-Cas9 interactions, covalently linked Cas9 orthologs (orthogonal Cas9-Cas9 chimeras),^12^ here dubbed forced Cas9 heterodimers, ensure the formation of functional CRISPR-Cas9 multiplexes that further maximize the accumulation of precise deletions over unintended genomic modifications (Figure S1).

In this study, we hypothesized that advanced multiplexing genome editing approaches based on forced Cas9 heterodimers should profit from synchronous and stoichiometric assembly of the attendant reagents in target cells. However, delivering the required large and multicomponent reagents in such a fashion is challenging, especially in hard-to-transfect cell types such as those with potential or established therapeutic potential. Although viral vectors have a proven track record in achieving efficient and non-cytotoxic delivery of genome editing tools into hard-to-transfect cells, *in vitro* and *in vivo*, commonly used AAV vectors cannot deliver large genetic cargoes due to their limited DNA packaging capacity (<4.7 kb).^13^^,^^14^ Therefore, we sought to investigate the potential of high-capacity adenoviral vectors, henceforth dubbed adenovector particles (AdVPs), for genome editing involving conventional and advanced multiplexing strategies based on split and forced CRISPR-Cas9 heterodimers, respectively. Indeed, AdVPs congregate a valuable set of features for this purpose, namely (1) lack of viral genes, (2) vast packaging capacity (up to 36 kb), (3) high genetic stability, (4) amenability to straightforward cell-tropism modifications, and (5) efficient transduction of dividing and post-mitotic cells.^15^^,^^16^^,^^17^ AdVPs achieved efficient transfer of forced Cas9 heterodimers alone or together with their cognate gRNAs (forced CRISPR-Cas9 heterodimers) into muscle progenitor cells (myoblasts) from healthy (wild-type) and Duchenne muscular dystrophy (DMD) individuals. Importantly, as *S*. *pyogenes* Cas9:gRNA complexes can present high off-target activities, we assembled next-generation forced Cas9 heterodimers in which the *S*. *pyogenes* protein component is eCas9^4NLS^, a variant of the high-specificity SpCas9 nuclease eSpCas9(1.1)^18^ whose improved performance results from having two extra nuclear localization signals.^19^ Moreover, a dual gRNA pair in which the *S*. *pyogene*s gRNA component has an optimized scaffold^20^ was used to direct forced CRISPR-Cas9 heterodimers to the repair of defective alleles underlying DMD.

DMD (MIM no. 310200) is a lethal muscle-wasting X-linked disorder caused by loss-of-function mutations in the vast (∼2.4 Mb) dystrophin-encoding *DMD* gene (prevalence: ∼1 in 4,700 boys).^21^ Although *DMD* segmental duplications and point mutations give rise to this pathology, most DMD-causing mutations consist of intragenic deletions comprising one or more exons that disrupt the mRNA reading frame.^22^ Crucially, it is known that in-frame deletions within the *DMD* gene can yield internally truncated dystrophin proteins whose partial functionality causes a less severe muscular dystrophy, named Becker muscular dystrophy (MIM no. 300376). Hence, targeted removal of reading frame-disrupting mutations that result in in-frame mRNA transcripts encoding shorter, yet partially functional, Becker-like dystrophins have therapeutic potential.^21^^,^^23^ The assembly of Becker-like dystrophins has been achieved via multiplexing genome editing strategies in DMD patient-derived myoblasts,^11^^,^^24^^,^^25^^,^^26^^,^^27^ induced pluripotent stem cells,^28^ and dystrophic Dmd^*mdx*^ mice.^29^^,^^30^^,^^31^^,^^32^ These experiments involved the use of different agents to deliver dual programmable nucleases based on zinc fingers, transcription activator-like effectors, and CRISPR systems (reviewed in Maggio et al.^33^).

In this study, we build on the AdVP platform to demonstrate that transferring forced Cas9 heterodimers rather than each Cas9 component separately, increases the frequency of precise targeted DNA deletions while decreasing the extent of unintended genomic modifications. Significantly, single AdVPs assembled for all-in-one transfer of forced CRISPR-Cas9 heterodimers (i.e., forced Cas9 heterodimers and their respective gRNAs), further improves the performance of multiplexing genome editing. Finally, transduction experiments using AdVPs encoding forced CRISPR-Cas9 heterodimers combined with DNA- and protein-level assays established robust *DMD* exon 51 splice site motif excision resulting in reading frame restoration and dystrophin synthesis in unselected DMD patient-derived muscle cell populations.

## Results

### AdVP delivery of forced Cas9 heterodimers promotes targeted DNA deletions

AdVP capsids have potential for packaging and delivering full-length forced Cas9 heterodimer constructs alone or together with their cognate gRNA units. Moreover, through interactions with ubiquitously expressed CD46 receptors, AdVPs with fiber motifs from species B adenoviruses, such as those from type-50, transduce otherwise refractory coxsackievirus and adenovirus receptor-negative cells with established and potential therapeutic relevance, including stem cells and progenitor cells from the hematopoietic and skeletal muscle systems, respectively.^34^^,^^35^^,^^36^ Finally, compared with earlier-generation viral gene-containing adenoviral vectors, AdVPs have dampened cytotoxicity *in vitro* and immunogenicity *in vivo*, which bodes well for their potential clinical translation involving endogenous gene repair paradigms.^16^

**Figure 1:**
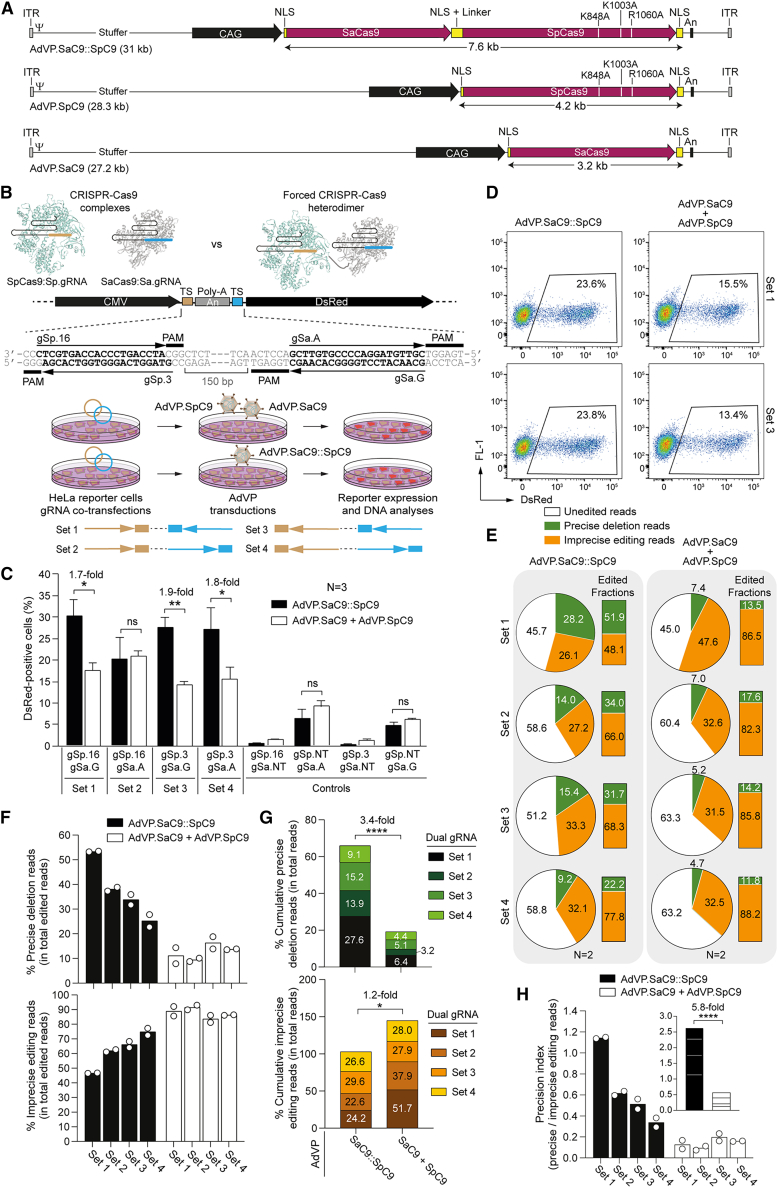
AdVP delivery of forced Cas9 heterodimers incudes robust targeted DNA deletions (A) Schematics of AdVP genomes. AdVP.SaCas9 and AdVP.SpC9 encode, respectively, *S*. *aureus* SaCas9 (SaC9) and eCas9^4NLS^ (SpC9), a variant of the high-specificity *S*. *pyogenes* eSpCas9(1.1) nuclease. The point mutations conferring enhanced specificity to SpC9 are specified. AdVP.SaC9::SpC9 encodes a forced Cas9 heterodimer consisting of SaCas9 fused to eCas9^4NLS^ (SaC9::SpC9). The synthesis of all nucleases is driven from the hybrid CAG regulatory sequences. An, polyadenylation signal; NLS, nuclear localization signal; ITR and Ψ, adenoviral inverted terminal repeats and packaging signal *cis*-acting elements required for vector DNA replication and encapsidation, respectively. (B) Workflow of the functional readout for assessing multiplexing gene editing. Reporter HeLa.dsRed^TS.An.TS^ cells were used for tracking multiplexing gene editing outcomes with conventional (split) and forced (linked) Cas9 heterodimers. Upper panel: HeLa.dsRed^TS.An.TS^ cells encode a dsRed reporter whose expression is dependent on the elimination of a polyadenylation signal (An) located between the CMV promoter and the reporter ORF. The protospacer and protospacer adjacent motif (PAM) sequences corresponding to the target sites (TS) for SpCas9 gRNAs (i.e., gSp.16 and gSp.3) and SaCas9 gRNAs (i.e., gSa.A and gSa.G), are indicated. Lower panel: generic experimental design. HeLa.dsRed^TS.An.TS^ are exposed to different combinations of gRNA pairs (dual gRNAs) via plasmid co-transfections and subsequently are co-transduced with AdVP.SaC9 and AdVP.SpC9 or transduced with AdVP.SaC9::SpC9. Multiplexing gene editing outcomes at the cellular and molecular levels are evaluated through flow cytometry and amplicon deep sequencing analyses, respectively. (C) Quantification of targeted DNA deletions. HeLa.dsRed^TS.An.TS^ cells were transfected with four different combinations of dual gRNAs (i.e., sets 1 through 4) and then transduced with AdVP.SaC9::SpC9 or co-transduced with AdVP.SaC9 and AdVP.SpC9. The target sites of each CRISPR-Cas9 complex are represented in the upper schematics, with boxes and arrows indicating PAM and gRNA protospacer orientations, respectively. Target DNA deletion frequencies were measured by dsRed-directed flow cytometry at 3 days post-transduction. Data are shown as mean ± SEM of three independent biological replicates. Significant differences between the indicated datasets were determined by two-tailed Student’s t tests; ∗∗p < 0.01, ∗p < 0.05; p > 0.05 was considered non-significant (ns). (D) Representative flow cytometry dot plots of HeLa.dsRed^TS.An.TS^ cells transfected and transduced with the specified reagents. (E–G) Characterization of gene editing outcomes through amplicon deep sequencing. The precise deletion and imprecise editing read frequencies within the total edited read counts and the cumulative precise deletion and imprecise editing read frequencies within the total read counts in reporter cells exposed to the indicated gene editing reagents are shown in (F) and (G), respectively. The next-generation sequencing analysis was performed on genomic DNA from two independent biological replicates (∼50,000 paired-end reads per sample). Significant differences between the indicated datasets were determined by two-way ANOVA; ∗p < 0.05, ∗∗∗∗p < 0.0001. (H) Multiplexing DNA editing precision in reporter cells. gRNA set-specific and cumulative (inset) precision index plot corresponding to the ratios between precise and imprecise editing read frequencies in HeLa.dsRed^TS.An.TS^ cells transfected with gRNA sets 1 through 4 and transduced or co-transduced with the indicated AdVPs.

Hence, we started by assembling AdVPs displaying type-50 fibers and encoding a forced Cas9 heterodimer (SaC9::SpC9) consisting of the *S*. *aureus* SaCas9 nuclease (SaC9)^5^ fused through a flexible linker to eCas9^4NLS^ (SpC9). SpC9 is a variant of the *S*. *pyogenes* high-specificity eSpCas9(1.1) nuclease^18^ whose enhanced activity results from having two additional nuclear localization signals.^19^ The resulting vector AdVP.SaC9::SpC9 was produced together with control AdVP.SaC9 and AdVP.SpC9 vectors encoding, respectively, SaC9 and SpC9 separately (Figure 1A). To test AdVP-assisted multiplexing genome editing strategies aimed at targeted chromosomal DNA deletions, we first generated HeLa.dsRed^TS.An.TS^ reporter cells containing a conditional *dsRed* expression unit at the *AAVS1* safe harbor locus. In these cells, dsRed protein synthesis ensues upon the deletion of a polyadenylation signal (An) situated between the reporter and a constitutively active CMV promoter (Figure 1B). Four different sets of gRNA pairs, each tailored for the targeted excision of the intervening An sequence, were tested. The dual gRNAs were designed to engage their bipartite target sequences in different orientations and to have a constant spacing between their target sites (i.e., ∼150 bp) (Figure 1B). Multiplexing genome editing experiments in HeLa.dsRed^TS.An.TS^ cells were initiated by co-transfecting expression plasmids for specific gRNA pairs. Subsequently, the transfected cells were exposed to equivalent functional units of AdVP.SaC9::SpC9 or AdVP.SaC9 and AdVP.SpC9. As negative controls, HeLa.dsRed^TS.An.TS^ cells subjected to the same AdVP transduction conditions, were initially co-transfected with plasmids expressing dual gRNAs in which one member consisted of an irrelevant non-targeting gRNA. In cells exposed to three out of the four dual gRNA sets tested, higher levels of dsRed expression were detected by flow cytometry in cells transduced with AdVP.SaC9::SpC9 than in cells co-transduced with AdVP.SaC9 and AdVP.SpC9 (Figures 1C and 1D). One gRNA pair (i.e., set 2) yielded similar frequencies of dsRed-positive cells upon transduction with AdVP.SaC9::SpC9 and co-transduction with AdVP.SaC9 plus AdVP.SpC9 (Figure 1C). These data demonstrate that the forced Cas9 heterodimer construct SaC9::SpC9 is functional and capable of inducing robust RNA-programmable targeted DNA deletions regardless of the relative orientations of the bipartite target sequences of dual gRNAs.

Next, we sought to investigate the types of DNA editing events registered in AdVP-treated reporter cells through amplicon deep sequencing analysis. This genotyping analysis revealed that, independently of the dual gRNA set used, transfer of forced Cas9 heterodimers led to higher levels of precise DNA deletions than those obtained through the separate delivery of Cas9 heterodimer moieties (Figures 1E and 1F). Conversely, imprecise DNA modifications consisting in large part of indels at each target site and at chromosomal junctions were detected at lower frequencies in reporter cells exposed to SaC9::SpC9 than in cells subjected to SaC9 and SpC9 (Figures 1E and 1F). Indeed, cumulative analysis of precise and imprecise DNA modifications corresponding to the dual gRNA set aggregate showed a robust increase in the former and a modest yet statistically significant reduction in the latter events in cells transduced with AdVP.SaC9::SpC9 (Figure 1G). As a result, the precision index represented by the ratios between accurate and imprecise genome editing reads, was most favorable by a 5.8-fold factor in HeLa.dsRed^TS.An.TS^ cells transduced with AdVP.SaC9::SpC9 than in cells independently transduced with AdVP.SaC9 and AdVP.SpC9 (Figure 1H).

### AdVP delivery of forced Cas9 heterodimers achieves robust endogenous *DMD* gene repair

**Figure 2:**
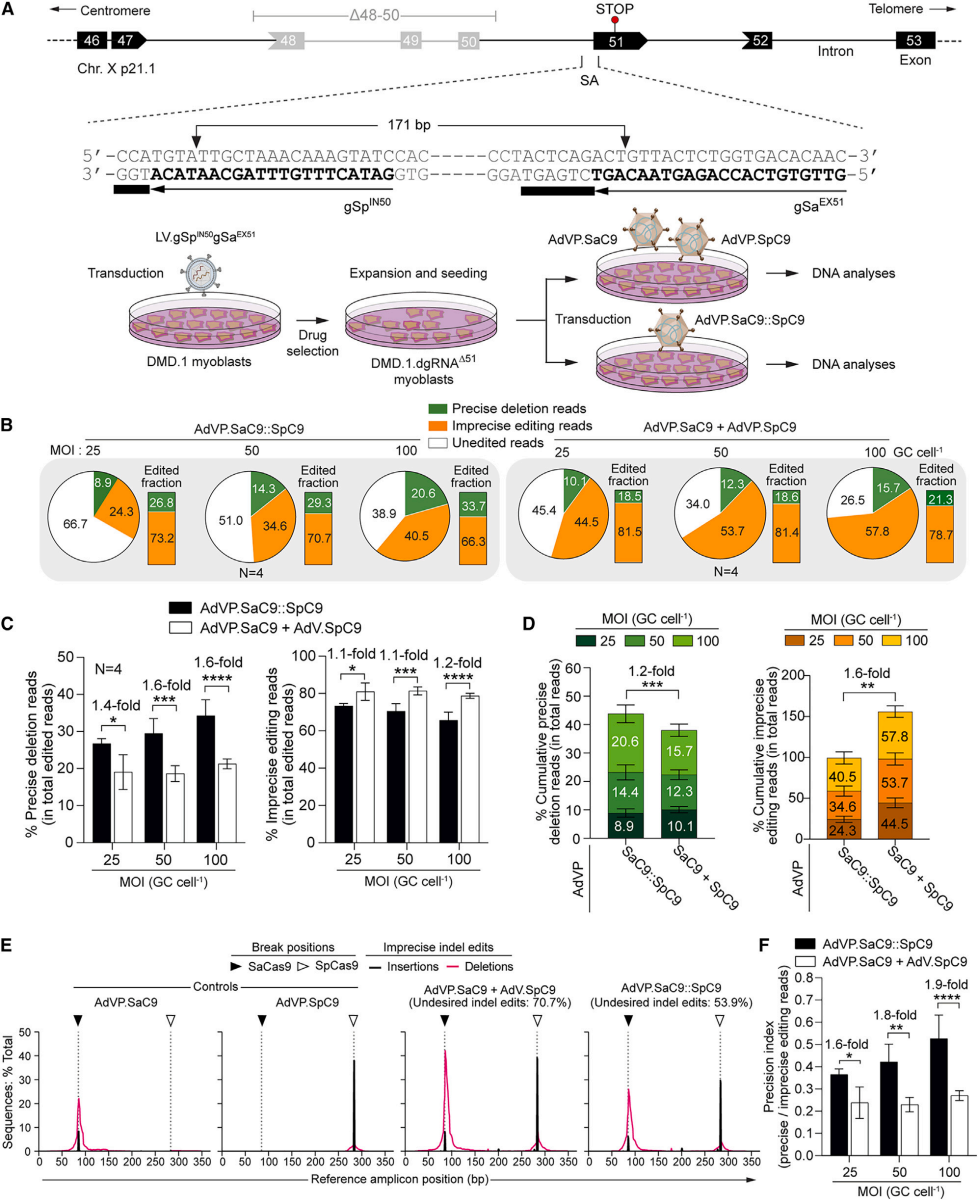
AdVP delivery of forced Cas9 heterodimers achieves efficient *DMD* gene repair (A) Schematics of the *DMD* gene repair strategy. *DMD* gene repair approaches based on the excision of the exon 51 splice acceptor (SA) motif after AdVP delivery of conventional or forced Cas9 heterodimers. Upper panel: the concomitant generation of DSBs at *DMD* intron 50 and exon 51 sequences by SpC9:gSp^IN50^ and SaC9:gSa^EX51^ complexes, respectively, leads to the removal of the intervening sequence containing the exon 51 SA motif. The ligation of the resulting chromosomal termini by NHEJ yields in-frame *DMD* transcripts coding for Becker-like dystrophins in muscle cells with out-of-frame *DMD* deletions. Lower panel: generic experimental design. Multiplexing *DMD* gene editing experiments were carried out in DMD.1 myoblasts (Δ48-50) stably expressing gSp^IN50^ and gSa^EX51^ upon transduction with a lentivector encoding both gRNAs and a drug resistance gene (i.e., DMD.1.dgRNA^Δ51^ myoblasts). Multiplexing gene editing outcomes in DMD.1.dgRNA^Δ51^ myoblasts transduced with AdVP.SaC9::SpC9 or co-transduced with AdVP.SaC9 and AdVP.SpC9 were evaluated through amplicon deep sequencing. (B–D) Quantification of *DMD* editing outcomes in DMD.1.dgRNA^Δ51^ myoblasts. The proportions between sequencing reads derived from unedited endogenous *DMD* alleles, precise deletions, and unintended edits in DMD.1.dgRNA^Δ51^ myoblasts exposed to AdVP.SaC9::SpC9 or to AdVP.SaC9 and AdVP.SpC9 at the indicated total MOIs, are displayed in (B). The precise deletion and imprecise editing read frequencies within the total edited read counts are plotted in (C) (left and right graph, respectively). The cumulative precise deletion and imprecise editing read frequencies within the total read counts in DMD.1.dgRNA^Δ51^ exposed to three MOIs of the indicated AdVPs are shown in (D) (left and right graph, respectively). The next-generation sequencing data were derived from four biological replicates with ∼50,000 paired-end reads analyzed per sample. (E) Representative indel profiles in DMD.1.dgRNA^Δ51^ myoblasts transduced with the indicated AdVPs at a total MOI of 100 GCs cell^−1^. The frequencies, types, and distributions of unintended indel “footprints” detected within the *DMD* target region are plotted. MOI, multiplicity of infection; GCs cell^−1^, genome copies per cell. Significant differences between the indicated datasets were determined by two-way ANOVA; ∗∗∗∗p < 0.0001, ∗∗∗p < 0.001, ∗∗p < 0.01, ∗p < 0.05. (F) Multiplexing DNA editing precision in DMD.1.dgRNA^Δ51^ myoblasts. Precision index plot corresponding to the ratios between precise-deletion to unintended-edit read frequencies in AdVP-treated DMD.1.dgRNA^Δ51^ myoblasts.

To investigate the performance of conventional (untethered) vis-a-vis forced (tethered) Cas9 heterodimers at an endogenous human locus, we selected a dual gRNA composed of *S*. *pyogenes* gRNA gSp^IN50^ and *S*. *aureus* gRNA gSa^EX51^ (dgRNA^Δ51^) designed for NHEJ-mediated excision of the *DMD* exon 51 splice acceptor (SA) coding motif upon site-specific cleavage at intron 50 and exon 51, respectively (Figure 2A, top panel). Targeted SA elimination is expected to induce exon skipping during pre-mRNA processing resulting in reading frame restoration and ensuing Becker-like dystrophin expression in muscle cells from 13%–14% of DMD patients.^22^ Among these are patients with deletions of *DMD* exons 48 through 50 (Δ48-50). To this end, we started by transducing myoblasts from a donor with the *DMD* Δ48-50 genotype (DMD.1 myoblasts) stably expressing dgRNA^Δ51^ (DMD.1.dgRNA^Δ51^ myoblasts) with AdVP.SaC9::SpC9 or with AdVP.SaC9 and AdVP.SpC9, at total multiplicities of infection (MOIs) of 25, 50, and 100 genome copies per cell (GCs cell^−1^) (Figure 2A, bottom panel). Parallel cultures of DMD.1.dgRNA^Δ51^ myoblasts individually transduced with AdVP.SaC9 or AdVP.SpC9 at the same total MOI provided for negative controls. Amplicons diagnostic for the 171-bp genomic deletion encompassing the *DMD* exon 51 SA coding motif were readily detected in cells receiving conventional and forced Cas9 heterodimers (Figure S2). In addition to these deletion-specific PCR products, genotyping assays based on incubating PCR amplicons with the mismatch sensing T7 endonuclease I (T7EI) also detected a prevalence of imprecise indels resulting from the activity of SaC9:gSa^EX51^ and SpC9:gSp^IN50^ complexes at their target sequences (Figure S2).

To characterize genome editing outcomes in the form of precise deletions vs. unintended genomic modifications comprising indels at target sites or chromosomal deletion junctions, amplicon deep sequencing analysis was performed on AdVP-treated DMD.1.dgRNA^Δ51^ myoblasts. Consistent with the transduction experiments in HeLa.dsRed^TS.An.TS^ cells, this sensitive genotyping analysis showed that forced Cas9 heterodimers yielded precise deletions and imprecise genomic modifications at higher and lower rates, respectively, than those induced by untethered Cas9 heterodimers (Figures 2B–2E), which, in turn, resulted in an ∼1.8-fold increase in the precision index for AdVP.SaC9::SpC9 (Figure 2F). Equally consistent with the next-generation sequencing and T7EI genotyping assays in reporter HeLa.dsRed^TS.An.TS^ cells and engineered DMD.1.dgRNA^Δ51^ myoblasts (Figures 1E–1H and S2, respectively), the majority of genomic modifications consisted of imprecise DNA editing products independently of the MOI used as indicated by precision indexes below 1 (Figure 2F).

### All-in-one AdVP delivery of forced CRISPR-Cas9 heterodimers enhances *DMD* gene repair precision

**Figure 3:**
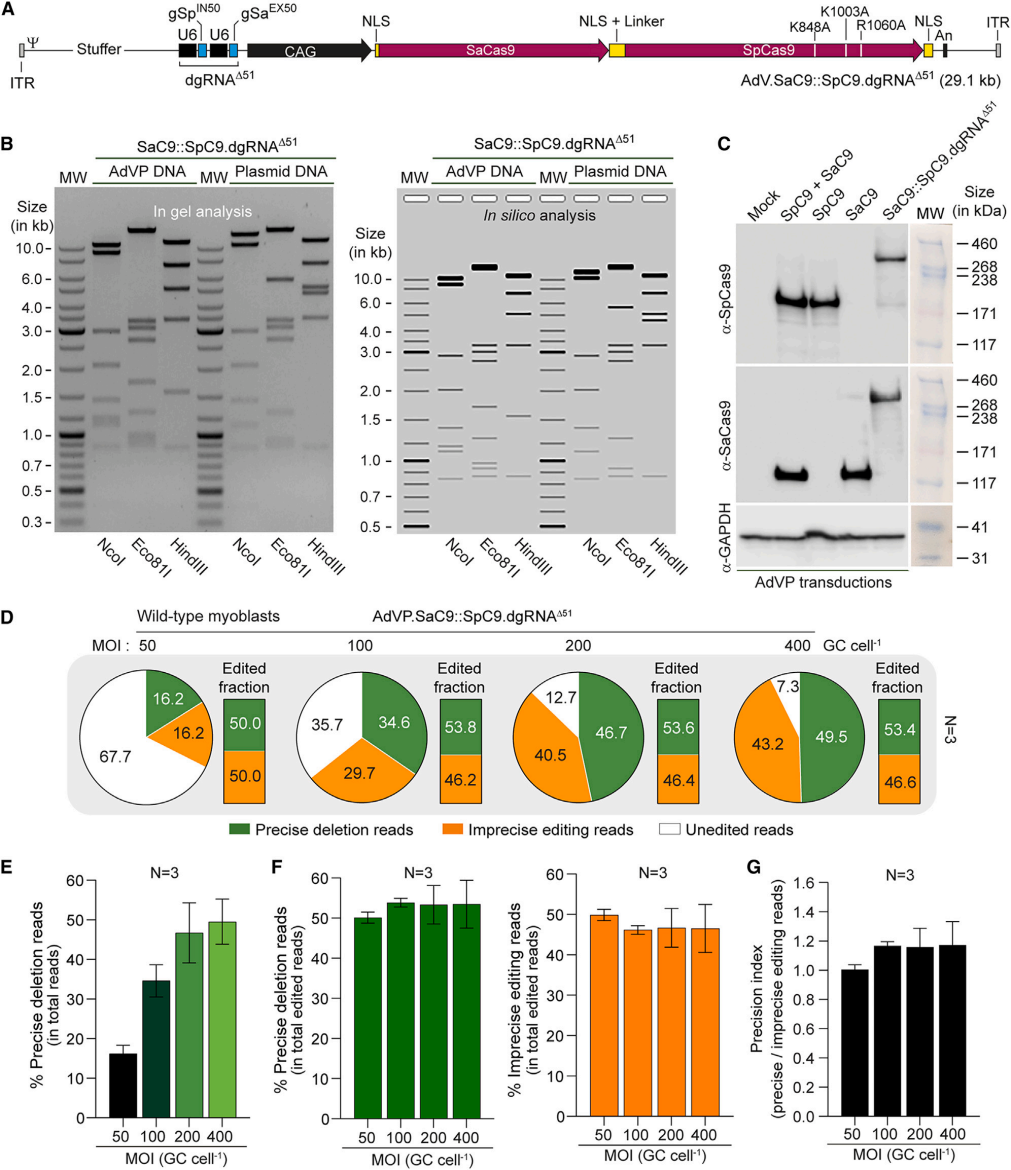
All-in-one AdVP delivery of forced CRISPR-Cas9 heterodimers promotes precise *DMD* gene repair (A) Schematics of AdVP for all-in-one delivery of forced CRISPR-Cas9 heterodimers. AdVP.SaC9::SpC9.dgRNA^Δ51^ encodes the forced Cas9 heterodimer SaC9::SpC9 composed of the SaCas9 nuclease covalently linked through flexible linkers to the optimized high-specificity SpCas9 nuclease variant eCas9^4NLS^. The synthesis of SaC9::SpC9 and of the dual gRNA pair gSp^IN50^ and gSa^EX51^ (dgRNA^Δ51^) is controlled via the hybrid CAG regulatory sequences and the human *U6* promoter, respectively. An, polyadenylation signal; NLS, nuclear localization signal; ITR and Ψ, adenoviral inverted terminal repeats and packaging signal *cis*-acting elements for vector DNA replication and encapsidation, respectively. (B) Assessing AdVP.SaC9::SpC9.dgRNA^Δ51^ DNA integrity. Restriction fragment length analysis (RFLA) of vector DNA isolated from purified AdVP.SaC9::SpC9.dgRNA^Δ51^ particles. *In silico* and in gel RFLA analyses are presented. Marker, GeneRuler DNA Ladder molecular weight mix. Parental circular plasmid served as additional molecular weight references. (C) Assessing full-length SaC9::SpC9 synthesis upon AdVP transduction. Western blot analysis was performed on wild-type myoblasts exposed to AdVP.SaC9::SpC9.dgRNA^Δ51^ or to AdVP.SaC9 and AdVP.SpC9 at a total MOI of 400 GCs cell^−1^ at 3 days post-transduction. Detection of GAPDH provided for protein loading controls. MW, HiMark Pre-Stained Protein Standard molecular weight marker. (D–G) Deep sequencing analysis of *DMD* editing outcomes in wild-type myoblasts. The proportions between sequencing reads corresponding to unmodified *DMD* alleles, precise deletions, and unintended edits in wild-type myoblasts transduced with AdVP.SaC9::SpC9.dgRNA^Δ51^ at the indicated MOIs, are presented in (D). The precise deletion read frequencies within the total read counts and within the total edited read counts are depicted in (E) and in the left graph of (F), respectively; while the unintended editing read frequencies within the total edited read counts are presented in the right graph of (F). (G) Multiplexing DNA editing precision in wild-type myoblasts. Precision index plot corresponding to the relation between deletion to imprecise editing reads in wild-type myoblasts transduced with AdVP.SaC9::SpC9.dgRNA^Δ51^ at the indicated MOIs. MOI, multiplicity of infection; GCs cell^−1^, genome copies per cell.

To streamline and further investigate AdVP-assisted multiplexing *DMD* gene repair, we next generated vector AdVP.SaC9::SaC9.dgRNA^Δ51^ for all-in-one delivery of forced CRISPR-Cas9 heterodimers comprising SaC9::SpC9 and dgRNA^Δ51^ (Figure 3A). AdVP.SaC9::SaC9.dgRNA^Δ51^ was produced to a high titer (i.e., 9.8 × 10^10^ GC mL^−1^) and, importantly, restriction fragment length analysis of DNA isolated from purified particles revealed that packaged vector genomes retained their structural integrity (Figure 3B). Moreover, synthesis of forced full-length Cas9 heterodimers was established by western blot analysis of wild-type myoblasts transduced with AdVP.SaC9::SaC9.dgRNA^Δ51^ (Figure 3C). Interestingly, confocal immunofluorescence microscopy disclosed that, in contrast to SpC9 and SaC9, the SpC9::SaC9 fusion product was, in most cells, prevalently found in the cytoplasm despite having more NLS motifs than its constituent subunits (Figure S3). This sub-cellular distribution suggests that the large size of SpC9::SaC9 is a contributing factor to its relatively lower nuclear translocation capability. Regardless, T7EI genotyping assays on wild-type myoblasts transduced with AdVP.SaC9::SaC9.dgRNA^Δ51^ showed a clear dose-dependent increase in the frequency of targeted DNA deletions (Figure S4), and lower amounts of imprecise genome editing events than those observed in engineered DMD.1.dgRNA^Δ51^ myoblasts transduced with AdVP.SaC9::SpC9 (compare Figure S2 with Figure S4). Amplicon deep sequencing analysis of wild-type myoblasts transduced with AdVP.SaC9::SaC9.dgRNA^Δ51^ confirmed that targeted DNA deletions occurred in an AdVP dose-dependent manner (Figures 3D and 3E). Significantly, regardless of the MOI used, the distribution between precise and imprecise genome editing events (Figures 3D and 3F) was more balanced than that previously observed in DMD.1.gRNA^Δ51^ myoblasts exposed to either single AdVP.SaC9::SpC9 or to dual AdVP.SaC9 and AdVP.SpC9 vectors (Figure 2B). Indeed, all-in-one AdVP delivery of forced CRISPR-Cas9 heterodimer components resulted in precision indexes at or above 1 (Figure 3G).

**Figure 4:**
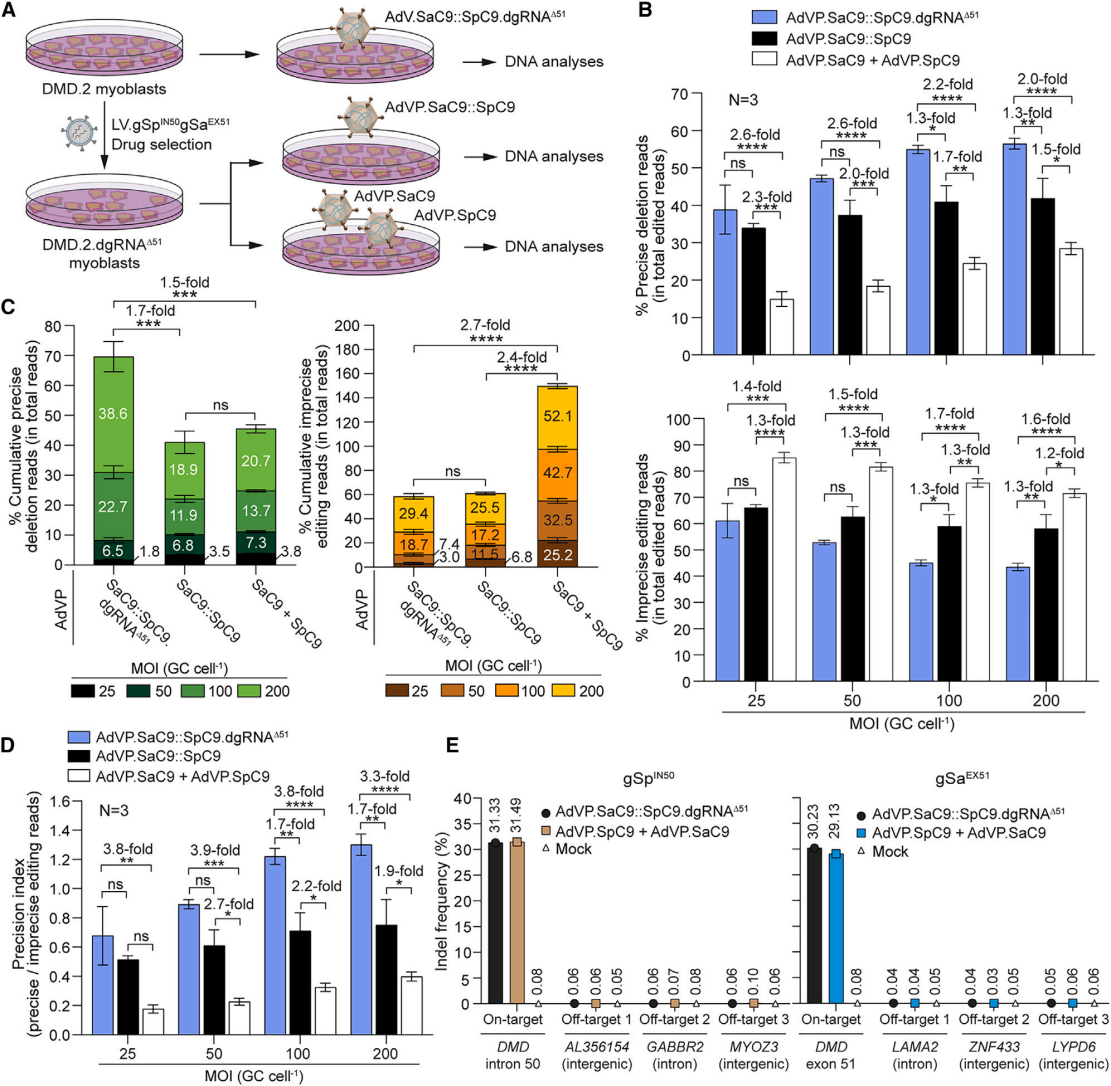
All-in-one AdVP delivery of forced CRISPR-Cas9 heterodimers enhances *DMD* gene repair precision (A) Schematics of the experimental design. Multiplexing *DMD* gene editing experiments were performed in parental DMD.2 myoblasts (Δ48-50) and their derivatives stably expressing gSp^IN50^ and gSa^EX51^ upon transduction with a lentivector encoding both gRNAs and a drug resistance gene (i.e., DMD.2.dgRNA^Δ51^ myoblasts). Multiplexing gene editing outcomes in parental DMD.2 myoblasts transduced with AdVP.SaC9::SpC9.dgRNA^Δ51^ were compared with those registered in engineered DMD.2.dgRNA^Δ51^ myoblasts transduced with AdVP.SaC9::SpC9 or co-transduced with AdVP.SaC9 and AdVP.SpC9 at different total MOIs. (B and C) Amplicon deep sequencing analysis of *DMD* editing outcomes upon split vs. all-in-one delivery of CRISPR-Cas9 multiplex components. The precise deletion and unintended editing read frequencies within the total edited read counts are shown in (B) (top and bottom graph, respectively); while the cumulative precise deletion and unintended editing read frequencies within the total read counts obtained through the three different AdVP transduction conditions are presented in (C). Bars and error bars correspond to mean ± SEM from three biological replicates (∼50,000 paired-end reads per sample). (D) Multiplexing DNA editing precision upon split vs. all-in-one delivery of CRISPR-Cas9 multiplex components. Precision index plot corresponding to the ratios between precise deletion and unintended editing read frequencies in myoblasts subjected to the AdVP transduction conditions depicted in (A). Significant differences between the indicated datasets were determined by two-way ANOVA; ∗∗∗∗p < 0.0001, ∗∗∗p < 0.001, ∗∗p < 0.01, ∗p < 0.05; p > 0.05 was considered non-significant (ns). MOI, multiplicity of infection; GCs cell^−1^, genome copies per cell. (E) Assessing off-target DNA cleavage upon all-in-one AdVP transduction. Parental DMD.2 myoblasts and engineered DMD.2.dgRNA^Δ51^ myoblasts were exposed to AdVP.SaC9::SpC9.dgRNA^Δ51^ and to AdVP.SpC9 and AdVP.SaC9, respectively, at a total MOI of 200 GCs cell^−1^. DNA cleaving activities at the dual gRNA target sites in *DMD* intron 50 and *DMD* exon 51 and at three top-ranked candidate off-target sites for gSp^EX51^ (i.e., *AL356154*, *GABBR2*, and *MYOZ3*) and gSa^IN50^ (i.e., *LAMA2*, *ZNF433*, and *LYPD6*) were quantified by amplicon deep sequencing using ∼50,000 paired-end reads per sample.

Based on these data, we next sought to compare side-by-side the performance of *DMD* gene repair resulting from delivering multiplexing CRISPR genome editing components as independent or integrated units. To this end, we transduced myoblasts from a second donor with the *DMD* Δ48-50 genotype (i.e., DMD.2 myoblasts) with AdVP.SaC9::SaC9.dgRNA^Δ51^ and, in parallel, transduced these myoblasts stably expressing dual gRNA^Δ51^ (i.e., DMD.2.dgRNA^Δ51^) with either AdVP.SaC9::SpC9 or AdVP.SaC9 plus AdVP.SpC9 at equivalent total MOI (Figure 4A). Amplicon deep sequencing analysis showed a progressive increase in precise DNA deletions and concomitant decrease in imprecise DNA modifications in cell populations transduced with AdVP.SaC9 and AdVP.SpC9, AdVP.SaC9::SpC9 and AdVP.SaC9::SaC9.dgRNA^ΔE51^ (Figure 4B). This trend was observed at all MOIs tested and reached statistical significance in most transduction group comparisons (Figure 4B). Moreover, cumulative genome editing outcome analysis corresponding to the aggregate of the various MOIs confirmed that transductions with AdVP.SaC9::SaC9.dgRNA^Δ51^ yielded increased precise and decreased imprecise genomic edits when compared with those triggered by co-transductions with AdVP.SaC9 and AdVP.SpC9 (i.e., 1.5- and 2.7-fold, respectively) (Figure 4C). As a result, the precision indexes representing the relation between precise to imprecise editing read frequencies of AdVP.SaC9::SpC9.dgRNA^ΔE51^ transductions were higher than those of AdVP.SaC9 and AdVP.SpC9 co-transductions by a factor of ∼3- to 4-fold (Figure 4D). Moreover, AdVP.SaC9::SaC9.dgRNA^Δ51^ also led to higher precise DNA deletion frequencies than AdVP.SaC9::SpC9 (i.e., 1.7-fold) (Figure 4C). In contrast, AdVP.SaC9::SaC9 transductions, while capable of reducing imprecise edits by 2.4-fold, were not capable of increasing the precise DNA editing fractions when compared with AdVP.SaC9 and AdVP.SpC9 co-transductions (Figure 4C). Collectively, these data indicate that the performance of multiplexing genome editing procedures can profit from integrated all-in-one delivery of forced CRISPR-Cas9 heterodimer units. Importantly, amplicon deep sequencing analysis of unmodified DMD.2 myoblasts and engineered DMD.2.dgRNA^Δ51^ myoblasts transduced at the highest total MOI of 200 GCs cell^−1^ with AdVP.SaC9::SaC9.dgRNA^Δ51^ and AdVP.SaC9 together with AdVP.SpC9, respectively, showed background indel frequencies at top-ranked candidate off-target sequences for gSp^IN50^ and Sa^EX51^ (Figure 4E).

**Figure 5:**
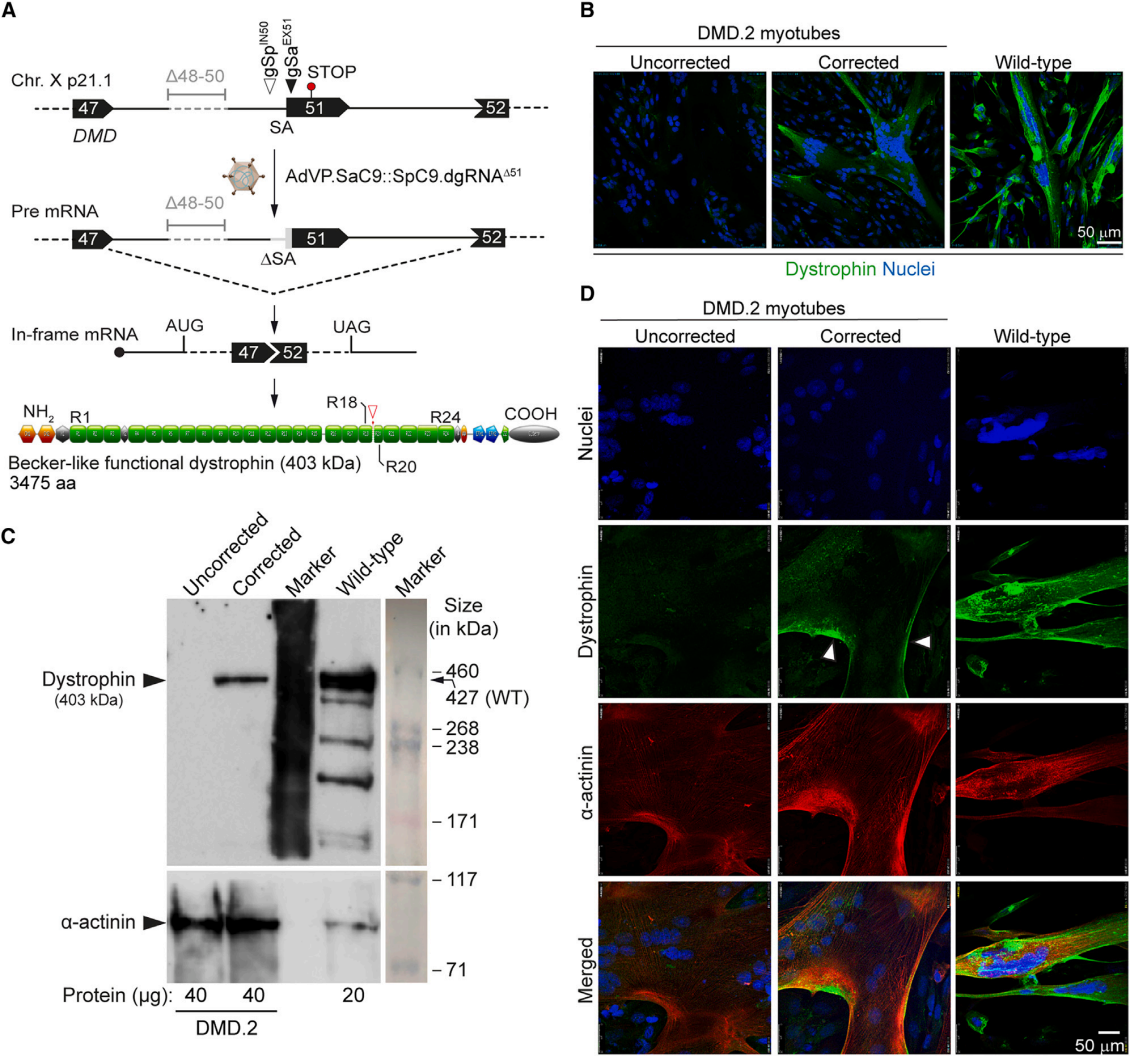
All-in-one AdVP delivery of forced CRISPR-Cas9 heterodimers rescues dystrophin synthesis in DMD muscle cells (A) Schematics of the *DMD* gene repair strategy and outcome. AdVP delivery of forced CRISPR-Cas9 heterodimers addressed to *DMD* intron 50 and exon 51 results in NHEJ-mediated excision of the exon 51 splice acceptor (SA) site motif leading to in-frame *DMD* transcript assembly and Becker-like dystrophin expression in muscle cells with out-of-frame *DMD* deletions. (B) Detection of dystrophin by fluorescence microscopy. Confocal immunofluorescence microscopy analysis on muscle cells differentiated from DMD.2 patient-derived myoblasts transduced with AdVP.SaC9::SpC9.dgRNA^Δ51^ at an MOI of 200 GCs cell^−1^ (corrected). Mock-transduced DMD.2 myoblasts (uncorrected) and healthy donor-derived myoblasts (wild-type) subjected to the same myogenic differentiation conditions served as negative and positive controls, respectively. Immunostaining was done at 10 days post-transduction and nuclei were labeled with the DNA dye DAPI. (C) Detection of dystrophin by western blot analysis. Western blotting was performed on muscle cells differentiated from DMD.2 patient-derived myoblasts transduced with AdVP.SaC9::SpC9.dgRNA^Δ51^ at an MOI of 200 GCs cell^−1^ (corrected). Mock-transduced DMD.2 myoblasts (uncorrected) and healthy donor-derived myoblasts (wild-type) treated with the same myogenic differentiation conditions served as negative and positive controls, respectively. Differentiated wild-type muscle cells provided controls for the expression of endogenous full-length dystrophin and the skeletal muscle differentiation marker sarcomeric α-actinin. Marker, HiMark Pre-Stained Protein Standard molecular weight marker. (D) Assessing the differentiation capacity of AdVP-edited muscle cells. Confocal microscopy co-detection of dystrophin and sarcomeric α-actinin was performed on muscle cells differentiated from DMD.2 myoblasts initially transduced with AdVP.SaC9::SpC9.dgRNA^Δ51^ at an MOI of 200 GCs cell^−1^ (corrected). Mock-transduced DMD.2 myoblasts (uncorrected) and healthy donor-derived myoblasts (wild-type) exposed to the same myogenic differentiation conditions served as negative and positive controls, respectively. The co-immunostaining was done at 10 days post-transduction and nuclei were labeled with the DNA dye DAPI.

Encouraged by the collective data on the activity and specificity of forced CRISPR-Cas9 heterodimers, we proceeded to assess *de novo* assembly and expression of Becker-like dystrophin molecules upon multiplexing *DMD* gene repair with AdVP.SaC9::SaC9.dgRNA^Δ51^ (Figure 5A). To this end, DMD.2 myoblasts were transduced with AdVP.SaC9::SaC9.dgRNA^Δ51^ and subsequently induced to differentiate into syncytial myotubes. Fluorescence microscopy and western blot analyses readily led to the detection of Becker-like dystrophin synthesis specifically in the cultures containing myotubes differentiated from AdVP.SaC9::SaC9.gRNA^Δ51^-transduced myoblasts (Figures 5B and 5C, respectively). Finally, co-detection of the late skeletal muscle marker sarcomeric α-actinin and Becker-like dystrophin in myotubes by confocal microscopy confirmed the differentiation capacity of the DMD muscle progenitor cells edited through AdVP delivery of forced CRISPR-Cas9 heterodimers addressed to the *DMD* intron 50-exon 51 junction (Figure 5D).

## Discussion

Rapid progression in the genome editing field is contributing to widen the options for tackling complex scientific questions and developing candidate gene and cell therapies. The integration of gene delivery and gene editing systems is expected to advance this field by enabling efficient and predictable genetic modification of relevant target cell types *in vitro* and *in vivo*. Yet, an emerging trend concerns the fact that precision genome editing is increasingly underpinned by large and multicomponent reagents that render the application of commonly used delivery agents, such as AAV, cumbersome or ineffective.^33^ As a case in point, forced Cas9 heterodimer proteins and cognate orthogonal dual gRNAs constitute robust and versatile genome editing tools owing to the cooperative action of the resulting CRISPR-Cas9 multiplexes. Indeed, plasmid co-transfection experiments in HEK293T cells demonstrated that forced Cas9 heterodimers, besides promoting the generation of precise DNA deletions, permit cleaving otherwise refractory target sites with non-canonical PAMs, thus enlarging the genome editing targeting range.^12^ Presumably these attributes result from the increased effective concentration of CRISPR-Cas9 multiplexes at target sequences in a synchronous fashion (Figure S1). However, the specificity conferred by forced Cas9 heterodimers based on wild-type nucleases is not superior to that of untethered Cas9 proteins.^12^ In addition, productive and reproducible delivery of Cas9 fusion constructs in hard-to-transfect cells is severely limited due to their large size.

In this study, we have hence introduced a next-generation forced Cas9 heterodimer architecture (SaC9::SpC9) consisting of SaCas9 fused through a flexible linker to the enhanced high-specificity eCas9^4NLS 19^ and, using this tool, demonstrate the capacity of AdVPs to package functional Cas9 fusion constructs together with dual gRNA units. In doing so, we overcome the bottlenecks associated with the production and delivery of forced CRISPR-Cas9 heterodimer components for multiplexing gene editing purposes in hard-to-transfect dividing and non-dividing cells. AdVP transduction experiments comparing the performance of separated vs. integrated delivery of multiplexing genome editing components supports the importance of introducing such components in an integrated and stoichiometric fashion (Figure S5).

Hence, it is possible that the efficiency as well as the accuracy of other advanced genome editing principles based on large and multicomponent reagents will equally profit from integrated all-in-one delivery approaches. Prime editing (PE),^37^ for instance, depends on the coordinated action between a Cas9 nickase fused to an engineered reverse transcriptase (prime editor) and, often, dual gRNAs in which one or both members are extended as PE gRNAs (pegRNAs). In fact, recently, dual PE complexes consisting of a prime editor protein and two pegRNAs specifying bipartite target sequences and edits of interest, have been successfully tailored for inserting or deleting specific DNA tracts upon plasmid co-transfections in diverse cell lines.^38^^,^^39^^,^^40^^,^^41^^,^^42^^,^^43^ Previously, by exploiting the cell-cycle independent AdVP platform to transduce dividing and post-mitotic cells alike, work in our laboratory had demonstrated that the activity of single PE complexes is significantly promoted in cycling cells.^44^ Hence, it should be informative to investigate dual PE- and NHEJ-based multiplexing genome editing endpoints in *ex vivo* and *in vivo* settings using AdVP delivery as these settings are normally associated with cycling and post-mitotic cells, respectively.

Candidate *in vivo* and *ex vivo* DMD genetic therapies under investigation present their own sets of pros and cons.^33^ Current experimental cell therapies for muscular dystrophies based on the transplantation of *ex vivo* corrected myogenic cells present important bottlenecks, including limited cell survival, migration, and tissue engraftment.^45^^,^^46^ On the other hand, *ex vivo* approaches offer a controlled gene correction environment, bypass vector-neutralizing antibodies, and minimize direct contact between the patient and immunogenic components.^33^ In this study, AdVP delivery of forced CRISPR-Cas9 heterodimers was directed to the targeted excision of the *DMD* exon 51 splice acceptor motif in DMD muscle progenitor cells to evaluate *DMD* reading frame restoration and ensuing detection of dystrophin molecules. This NHEJ-based *DMD* gene editing strategy, amenable to 13%–14% of the DMD patient population,^22^ readily led to the synthesis of Becker-like dystrophins in syncytial muscle cell populations differentiated from unselected DMD myoblasts.

In conclusion, in this study, we have introduced a next-generation forced Cas9 heterodimer construct for RNA-programmable installation of chromosomal deletions in an efficient, specific, and accurate manner. In addition, we demonstrate the feasibility of using AdVPs to package forced CRISPR-Cas9 constructs for stoichiometric delivery of synchronously acting multiplexing gene editing components into human cells. Finally, by capitalizing on AdVPs to similarly transfer genome editing tools independently of their size, we found that integrated rather than separated delivery of forced CRISPR-Cas9 heterodimer units can foster the precision of targeted DNA deletions at the expense of unintended genomic modifications. Collectively, our data provide insights that can guide the future development and application of genome editing principles that depend on the balanced delivery and coordinated action of individual parts.

## Methods

### Cells

HeLa cells (American Type Culture Collection), EGFP expressing HeLa cell-derived H27 cells,^47^ and HeLa.dsRed^TS.An.TS^ cells, conditionally expressing a dsRed reporter, were cultured in Dulbecco’s modified Eagle’s medium (DMEM; Thermo Fisher Scientific, cat. no. 41966-029) supplemented with 5% (v/v) fetal bovine serum (FBS) ultra-low endotoxin (Biowest, cat. no. S1860-500). The culture conditions for the human wild-type and *DMD*-defective myoblasts no. 6594 and no. AB1098, herein referred to as DMD.1 and DMD.2 myoblasts, respectively, have been described previously.^48^^,^^49^ In brief, these muscle progenitor cells were grown in Skeletal Muscle Cell Growth Medium (Ready-to-use) (PromoCell, cat. no. C-23060) supplemented with 20% FBS, 1× GlutaMAX (Thermo Fisher Scientific, cat. no. 35050) and 100 U mL^−1^ penicillin/streptomycin or in F10 medium (Thermo Fisher Scientific, cat. no. 41550021) supplemented with 10% FBS (Gibco, cat. no. 10500064), 10 ng μL^−1^ recombinant human basic fibroblast growth factor (Peprotech; cat. no. 100-18B), 1 μM dexamethasone (Sigma-Aldrich, cat. no. D2915), and 100 U mL^−1^ penicillin/streptomycin. The PEC3.30 AdVP packaging cells^27^ were maintained in high-glucose DMEM supplemented with 10% FBS, 10 mM MgCl_2_, and 0.4 μg mL^−1^ puromycin (Thermo Fisher Scientific, cat. no. A11138-03). HEK293T cells were maintained in DMEM supplemented with 5% FBS and 100 U mL^−1^ penicillin/streptomycin. The cells used in this study were mycoplasma-free and were kept at 37°C in humidified air atmospheres with 10% CO_2_ (i.e., HeLa, HeLa.dsRed^TS.An.TS^, and HEK293T cells), 5% CO_2_ (i.e., human myoblasts), or at 39°C in a humidified air atmosphere with 10% CO_2_ (i.e., PEC3.30 cells).

### Recombinant DNA

The construct AV44_pCAG.Cas9^D10A^.gRNA^S1^ encodes the *S*. *pyogenes* nicking enzyme SpCas9^D10A^ together with *AAVS1*-targeting gRNA^S1^.^50^ This construct together with AQ02_pDonor.AAVS1.CMV.TS.An.TS.dsRed was employed to knock in, at the *AAVS1* safe harbor locus, the conditional *dsRed* cassette via in trans paired nicking,^50^ resulting in the fluorescence-based reporter cell line HeLa.dsRed^TS.An.TS^. Plasmid AW42_pLV.gSp^IN50^gSa^EX51^ was used for the assembly of lentivector particles LV.gSp^IN50^gSa^EX51^ employed for the generation of myoblasts constitutively expressing a dual gRNA consisting of gSp^IN50^ and gRNA gSa^EX51^ (dgRNA^Δ51^) targeting *DMD* intron 50 and *DMD* exon 51, respectively. The former and latter gRNAs are compatible with the orthogonal Cas9 proteins SpCas9 and SaCas9, respectively. The gRNA expressing plasmids AZ43_gSp.16, BA21_gSp.3, AM51_gSp.ISceI, BB10_gSa.G, and AV73_gSa.A were assembled by inserting the annealed oligonucleotides listed in Table S1 into the BveI-digested *S*. *pyogenes* gRNA acceptor construct AY56_Sp.gRNA-acceptor,^19^ or into the Esp3I-digested *S*. *aureus* gRNA acceptor construct BPK2660 (Addgene plasmid no. 70709),^51^ herein named AZ46_Sa.gRNA-acceptor. The *S*. *pyogenes* gRNA expression units used in this study have mutations in the scaffold coding sequence that disrupt a cryptic RNA polymerase III terminator and extend a stabilizing gRNA duplex which, together, can contribute to improved DNA editing activities.^20^ AM51_gSp.ISceI encodes a non-targeting gRNA, i.e., gRNA^NT^. This gRNA is irrelevant in human cells as it addresses *S*. *pyogenes* Cas9 proteins to the recognition sequence of the *S*. *cerevisiae* I-SceI homing endonuclease. AM51_gSp.ISceI, and AZ46_Sa.gRNA-acceptor served as negative controls in transfection experiments. The annotated maps and nucleotide sequences of all the constructs generated for this study are available in Figures S6–S11.

### Cell transfections

HeLa cells were seeded at a density of 5 × 10^4^ cells per well of 24-well plates (Greiner Bio-One). Next, transfections were initiated by adding 1 mg mL^−1^ 25 kDa linear polyethyleneimine (PEI) (Polysciences) solution (pH 7.4) to the different plasmid mixtures diluted in 150 mM NaCl (Merck) to a final volume of 50 μL. The amounts of PEI and DNA (in μL and ng, respectively) as well as the compositions of each of the DNA mixtures corresponding to the different transfection reactions are specified in Tables S2–S4. After the addition of the PEI polycation, the transfection reactions were immediately and vigorously vortexed for 10 s, after which, DNA-PEI complexes were allowed to form for 15 min at room temperature (RT). The resulting DNA-PEI complexes were subsequently added directly into the culture media of the target cells and, after 6 h, the transfection media were substituted by regular culture media.

### Generation of a fluorescence-based reporter cell line

The fluorescence-based reporter system HeLa.dsRed^TS.An.TS^ consists of HeLa cells genetically modified with a conditional *dsRed* reporter cassette and a puromycin resistance gene. Expression of the dsRed fluorescent protein is dependent on the elimination of a polyadenylation signal located between a CMV promoter and the *dsRed* ORF. NHEJ-mediated DNA sequence deletion is made possible in the presence of CRISPR-Cas9 nucleases designed to cleave at target sites flanking the transcription termination sequence. The single-cell-derived clone HeLa.dsRed^TS.An.TS^ was obtained by targeted integration of the reporter cassette into the *AAVS1* safe harbor locus.^52^^,^^53^ The integration of the exogenous cassette was achieved through a seamless gene knockin strategy named in trans paired nicking^50^^,^^54^ based on simultaneous single-strand break formation at donor and acceptor DNA by CRISPR-Cas9 nickases, in this case, Cas9^D10A^:gRNA^S1^ complexes encoded by AV44_pCAG.Cas9^D10A^.gRNA^S1^.^50^ The annotated map and nucleotide sequence of the donor construct AQ02_Donor.S1.CMV.TS.An.TS.dsRed employed to generate the HeLa.dsRed^TS.An.TS^ reporter cells are available in Figure S6 of the supplemental information. The generation of this fluorescence-based reporter cell line was initiated by transfecting HeLa cells following the PEI-based protocol described above and the transfection scheme depicted in Table S2. After 3 days, the cells were transferred to a new plate containing regular growth medium and, 1 day later, the growth medium was supplemented with 1 μg mL^−1^ of puromycin. Parental mock-transfected cells served as negative controls during the drug selection procedure. A puromycin-resistant single-cell-derived clone was expanded and employed in all the genome editing experiments involving sequential plasmid transfections and AdVP transductions.

### Production and characterization of AdVPs

The AdVP molecular clones AO76_pHC-Ad.CAG.SaCas9, AW71_pHC-Ad.CAG.eCas9^4NLS^, AW78_pHC-Ad.CAG.SaCas9.link.eCas9^4NLS^, and X65_pHC-Ad.SaCas9.link.eCas9^4NLS^.dgRNA^Δ51^ were assembled through standard recombinant DNA techniques and then used for the production of the fiber-modified AdVPs AdVP.SaC9, AdVP.SpC9, AdVP.SaC9::SpC9, and AdVP.SaC9::SpC9.dgRNA^Δ51^, respectively. The annotated maps and relevant nucleotide sequences of the AdVP genomes are available in Figures S8–S11 of the supplemental information. The protocols used in the generation, purification, and characterization of the resulting fiber-modified AdVP stocks have been described previously in detail.^27^^,^^44^ In brief, to initiate AdVP production, PEC3.30 producer cells expressing bacteriophage P1 Cre recombinase and adenovirus type-5 *E1*- and *E2A*-encoded proteins were seeded at a density of 1.6 × 10^6^ cells per well of 6-well plates (Greiner Bio-One). The following day, 6.25 μg of MssI-linearized AdVP plasmid clones were diluted in a total volume of 200 μL of 150 mM NaCl to which 20.6 μL of a 1 mg mL^−1^ solution of 25-kDa linear PEI (Polysciences) was added. The transfection solutions were then immediately and thoroughly mixed in a vortex and subsequently incubated for 25 min at RT to let DNA-PEI complexes form before being added in a dropwise manner to the medium of the producer cells. Six hours post-transfection the medium was replaced with fresh medium containing *E1*-deleted helper AdV vector AdV.SRα.LacZ.1.50^55^ at an MOI of 40 infectious units per cell. The helper vector enables the expression of the proteins necessary for the replication and assembly of the AdVPs. In addition, by transferring the cells to the permissive temperature of 34°C, expression of a thermosensitive version of the adenovirus DNA-binding protein ensues in the PEC3.30 cells, further contributing to vector complementation. Producer cells were harvested upon helper-triggered full cytopathic effect and then were subjected to three cycles of freezing and thawing in liquid N_2_ and 37°C water baths, respectively. After centrifugation for 10 min at 2,000 × *g*, the supernatants containing the vector particles were recovered and employed in three subsequent amplifications rounds in producer cells co-transduced with helper AdV.SRα.LacZ.1.50. The AdVPs retrieved from the last propagation round, involving 20 T175-cm^2^ culture flasks, were purified by sequential block and continuous CsCl buoyant density ultracentrifugation steps. Finally, the purified AdVPs were de-salted by ultrafiltration through Amicon Ultra-15 100K MWCO filters (MerckMillipore, cat. no. UFC910024).

The transducing unit titers of purified AdVP stocks were determined through qPCR assays using iQ SYBR Green Supermix (Bio-Rad, cat. no. L010171C) and primers targeting the AdVPs packaging signal (ψ) listed in Table S5. Three 3-fold serial dilutions of the vector genomes extracted from the purified AdVP stocks using the DNeasy Blood & Tissue Kits (QIAGEN, cat. no. 69506) were diluted 1:100 and employed as qPCR templates. Eight 10-fold serial dilutions of a linearized parental plasmid stock containing 1 × 10^7^ GC μL^−1^ were used as qPCR templates to generate a standard curve. The qPCR primers, cycling conditions and reaction components are specified in Tables S5 and S6. Data analysis was performed by using the Bio-Rad CFX Manager 3.1 software and the titers were calculated based on the Ct values of standard curves and extracted AdVP genome dilutions. The AdVP titers obtained are listed in Table S7. In addition, the functional titers of AdVP.SaC9, AdVP.SpC9, and AdVP.SaC9::SpC9 were assessed by using an assay based on flow cytometric quantification of *EGFP* knockout frequencies in H27 indicator cells following gRNA and Cas9 nuclease delivery. To express the appropriate *EGFP*-targeting *S*. *pyogenes* and *S*. *aureus* gRNAs, H27 cells were first transfected according to the protocol described above and the transfection scheme depicted in Table S3. Next, these cells were transduced with a range of AdVP stock dilutions. Three days post-transduction the percentages of reporter-negative cells were determined through flow cytometry and used to calculate AdVP stock titers in gene knockout units per mL (Table S8).

The assessment of the structural integrity of packaged vector genomes in purified AdVP stocks of AdVP.SaC9::SpC9.dgRNA^Δ51^ was essentially carried out as described previously.^56^ In brief, 80 μL of purified AdVPs were treated with 8 μL of 10 mg mL^−1^ DNase I (Sigma-Aldrich, cat. no. 10104159001) at 37°C for 30 min. Next, 2.4 μL of 0.5 M EDTA (pH 8.0), 6 μL of 10% (w/v) sodium dodecyl sulfate (SDS), and 1.5 μL of 20 mg mL^−1^ proteinase K (Thermo Fisher Scientific, cat. no. EO0491) were added to inactivate the DNase I activity. Following an incubation at 55°C for 1 h, vector DNA isolation was completed by using the QIAEX II Gel Extraction Kit (QIAGEN, cat. no. 20021) according to the manufacturer’s instructions. The isolated vector genomes were then subjected to restriction enzyme fragment analysis by using the Gel-Doc XR+ system (Bio-Rad) and the Image Lab 6.0.1 software (Bio-Rad). Parental AdVP plasmid clones digested with the same restriction enzymes applied to vector genomes served as molecular weight references. The *in silico* restriction patterns corresponding to intact plasmid and vector DNA were made with the aid of the SnapGene (version 5.3.1) software.

### Production of lentivector particles

The lentivector LV.gSp^IN50^gSa^EX51^ was assembled according to previously detailed protocols.^57^^,^^58^ In brief, HEK293T cells were seeded in 175-cm^2^ culture flasks (Greiner Bio-One) and, the next day, were transfected with a 30-μg DNA mixture composed of lentivector shuttle plasmid AW42_pLV.gSp^IN50^gSa^EX51^ (Figure S7) packaging plasmid psPAX2 (Addgene plasmid no. 12260; a gift from Didier Trono), and VSV-G-pseudotyping plasmid pLP/VSVG (Invitrogen) at 2:1:1 (size-normalized for molecule copy number) diluted in 150 mM NaCl to a final 1-mL volume. Next, after receiving 90 μL of a 1 mg mL^−1^ PEI solution (25 kDa PEI; Polysciences), the transfection mixture was immediately and vigorously vortexed for approximately 10 s. After 15–20 min at RT the DNA-PEI complexes were diluted in 19 mL of culture medium and directly added to the HEK293T producer cells. After 24 h, the transfection medium was replaced by fresh DMEM supplemented with 5% FBS and, at 3 days post-transfection, the producer-cell-conditioned medium was harvested and the cellular debris were removed by centrifugation and filtration through 0.45-μm pore-sized HT Tuffryn membrane filters (Pall Life Sciences, cat. no. PN4184). The lentivector particle titer in the clarified supernatant was assessed by employing the RETROTEK HIV-1 p24 antigen ELISA kit (ZeptoMetrix, cat. no. 0801111). On the basis of the resulting physical particle concentration of 416 ng p24^gag^ mL^−1^ the functional lentivector dose applied for generating dual gRNA expressing DMD myoblasts was estimated by converting 1 ng of p24^gag^ antigen to 2,500 lentiviral vector transducing units.^59^

### Generation of myoblasts expressing dual gRNAs

The generation of DMD.1 and DMD.2 myoblasts constitutively expressing gSa^EX51^ and gSp^IN50^ was done via transduction with LV.gSp^IN50^gSa^EX51^. In brief, cells were seeded in regular growth medium at a density of 5 × 10^4^ cells per well of 24-well plates. The following day the cells were exposed to medium containing the lentivector at an MOI of 5 TU cell^−1^. After 2–3 days, the cells were transferred to a new plate containing regular growth medium and, 1 day later, the medium of DMD.1 and DMD.2 myoblasts was supplemented with 20 and 50 μg mL^−1^ of hygromycin B (Invitrogen, cat. no. 10687010), respectively. Parental mock-transduced cells served as negative controls during the drug selection procedure.

### Transduction experiments

Transduction experiments in HeLa.dsRed^TS.An.TS^ cells were initiated by seeding the cells in wells of 24-well plates at a density of 5 × 10^4^ cells per well. The next day, the cells were exposed to the appropriate gRNA constructs by using the PEI-based transfection protocol described above and the transfection mixtures indicated in Table S4. After 6 h the transfection medium was replaced by 500 μL of regular culture medium containing equivalent functional units of AdVPs. At 3 days post-transduction the cells were analyzed through dsRed-directed flow cytometry and were collected for the quantification and characterization of targeted genome-modifying events.

Transduction experiments in human myoblasts were initiated by seeding the myoblasts in wells of 24-well plates at a density of 5 × 10^4^ cells per well. The next day, the medium was replaced by 500 μL of medium containing AdVPs at different MOIs. At 3 days post-transduction, the myoblasts were transferred into wells of 6-well plates and, after reaching confluency, the myoblasts were collected for genomic DNA extraction to quantify and characterize genome-modifying events via next-generation deep sequencing analysis.

### Cell differentiation assays

Human myoblasts were transferred in regular culture medium into wells of 24- or 6-well plates pre-coated with a 0.1% (w/v) gelatin solution (Sigma-Aldrich, cat. no. G13393). After reaching full confluency, the muscle progenitor cells were exposed to myogenic differentiation medium consisting of phenol red-free DMEM (Thermo Fisher Scientific, cat. no. 11880–028) supplemented with 100 μg mL^−1^ human holo-transferrin (Sigma-Aldrich, cat. no. T0665), 10 μg mL^−1^ human insulin (Sigma-Aldrich, cat. no. I9278), and 100 U mL^−1^ penicillin/streptomycin. The differentiation of post-mitotic myotubes was assessed 4–5 days later by confocal immunofluorescence microscopy and western blot analyses using the antibodies specified in Tables S9 and S10, respectively.

### Flow cytometry

The frequencies of edited HeLa.dsRed^TS.An.TS^ cells were determined by reporter-directed flow cytometry using a BD LSR II flow cytometer (BD Biosciences). In brief, after large-volume PBS washes and trypsin treatments, the reporter cells were collected by centrifugation at 300 × *g* for 5 min and cell pellets were resuspended in PBS containing 0.5% bovine serum albumin (BSA) and 2 mM EDTA (pH 8.0) (FACS buffer). Mock-transduced cells served as control to establish background fluorescence levels. A minimum of 10,000 single live cells were acquired per sample and the resulting data were analyzed with the aid of the FlowJo 10.6.0 software (TreeStar).

### Target DNA cleaving assays

Targeted DSB formation in transduced cells was assessed by using genotyping assays based on the mismatch sensing T7EI enzyme. To this end, genomic DNA from mock-transduced and AdVP-transduced cells was isolated by using the DNeasy Blood & Tissue Kit (QIAGEN, cat. no. 69506) following the manufacturer’s recommendations. The *DMD*-specific PCR amplifications were performed with the Phusion High-Fidelity DNA Polymerase system (Thermo Fisher Scientific, cat. no. F-530). The primers, PCR mixtures, and cycling parameters used are specified in Tables S11 and S12. The resulting PCR amplicons were denaturated and reannealed by applying the thermocycling program indicated in Table S13. T7EI-based DNA cleaving assays were done as follows. First, 8 μL of each PCR mixture was incubated in 15-μL reactions consisting of 1× NEBuffer 2 (New England Biolabs, cat. no. B7002S) and 5 U of T7EI (New England Biolabs, cat. no. M0302). Next, after 15-min incubations at 37°C, the DNA samples were subjected to electrophoresis through 2% (w/v) agarose gels in 1× Tris-acetate-EDTA buffer. The resulting ethidium bromide-stained DNA species were then detected by using a Molecular Imager Gel-DocTM XR+ system (Bio-Rad) and analyzed via the Image Lab 6.0.1 software (Bio-Rad).

### Confocal immunofluorescence microscopy

Undifferentiated myoblasts and differentiated myotubes were analyzed through immunofluorescence microscopy analysis. Cells cultured on glass coverslips were fixed with 4% paraformaldehyde for 10 min. Next, after three washes with PBS, the cells were permeabilized in 0.5% (v/v) Triton X-100 in TBS (50 mM Tris-HCl [pH 7.5], 100 mM NaCl) at RT for 5 min, after which they were washed three times for 10 min with 0.1% Triton X-100 in TBS. Subsequently, the cells were incubated overnight at 4°C with a blocking Antibody Dilution Solution (Abdil) consisting of 0.1% Triton X-100, 2% BSA, and 0.1% sodium azide in TBS. The specimens were then exposed for 2 h at RT to the appropriate primary antibodies diluted in the blocking solution (Table S9). After three 5-min washes with 0.1% Triton X-100 in TBS, the specimens were incubated with fluorochrome-conjugated secondary antibodies diluted in blocking solution for 1 h in the dark at RT (Table S9). Finally, after three 10-min washes with 0.1% Triton X-100 in TBS, ProLong Diamond Antifade Mountant reagent containing DAPI (Thermo Fisher Scientific, cat. no. P36971) was used for mounting the specimens. Immunofluorescence microscopy images were acquired by using an upright Leica SP8 confocal microscope equipped with Leica hybrid detectors HyD. All images were analyzed through the LAS X (Leica Microsystems) and ImageJ (US National Institutes of Health) software packages.

### Western blotting

Cultures of differentiated myotubes were processed for western blot analysis as follows. After 4–5 days in differentiation medium, the myotube-containing cultures were lysed on ice for 30 min by incubation in 50 μL of RIPA buffer (Thermo Fisher Scientific, cat. no. 89900) supplemented with a protease inhibitor cocktail (cOmplete Mini, Sigma-Aldrich, cat. no. 11836153001). The resulting cell lysates were then passed through a 30-gauge syringe several times. Protein quantification was carried out by using the Pierce BCA Protein Assay Kit (Thermo Fisher Scientific, cat. no. 23225), following the manufacturer’s instructions. Next, the indicated amounts of total protein diluted in 4× sample buffer (Bio-Rad, cat. no. 161-0791) and 20× reducing agent (Bio-Rad, cat. no. 161-0792), were incubated at 95°C for 5 min. Protein samples and 15 μL of HiMark Prestained Protein Standard (Thermo Fisher Scientific, cat. no. LC5699) were loaded in a 3%–8% Criterion XT Tris-Acetate precast gel (Bio-Rad, cat. no. 3450130). The polyacrylamide gel was then placed in a Criterion Cell containing XT Tricine running buffer (Bio-Rad, cat. no. 1610790) and run for 30 min at 75 V (0.07 A) and for 1.5 h at 150 V (0.12 A). Subsequently, the resolved proteins were transferred to polyvinylidene difluoride (PVDF) membranes with the aid of a Trans-Blot Turbo Midi PVDF pack (Bio-Rad, cat. no. 1704157) and a Trans-Blot Turbo system (Bio-Rad) according to the manufacturer’s recommendations for high-molecular-weight proteins (2.5 A, 25 V, 10 min). The PVDF membranes were then blocked for 2 h at RT in 5% non-fat dry milk (Campina Elk, cat. no. 112349) dissolved in TBS with 0.1% (v/v) Tween 20 (TBST). Next, the membranes were incubated overnight at 4°C with primary antibodies (Table S10) diluted in 5% non-fat dry milk. After three 10-min washes in TBST, the membranes were incubated for 2 h at RT with the appropriate secondary antibodies (Table S10) conjugated to horseradish peroxidase (IgG-HRP) diluted in 5% non-fat dry milk. Proteins were detected by using horseradish peroxidase substrate Clarity Western ECL (Thermo Fisher Scientific, cat. no. 1705061) following the manufacturer’s specifications. The protein lysate samples employed for Cas9 detection were instead retrieved at 3 days post-transduction. The samples were lysed in Laemmli buffer consisting of 8.0% glycerol, 3% SDS, and 200 mM Tris-HCl (pH 6.8) and were subsequently incubated for 5 min at 100°C. Next, the samples underwent the same procedures as described above.

### Next-generation sequencing for on-target and off-target analyses

HeLa cells and human myoblasts that underwent various AdVP-based genome editing approaches were analyzed by amplicon deep sequencing to quantify and characterize the resulting genome editing events at on- and off-target sites. Genomic DNA isolated with the aid of the DNeasy Blood & Tissue kit reagents and protocol was used as input to a previously described amplicon deep sequencing analyses pipeline.^44^^,^^60^ In brief, the *DMD* exon 51 target region was first amplified with primers containing adapter tag overhangs using Phusion High-Fidelity Polymerase (Thermo Fisher Scientific, cat. no. F-530L). The primers, PCR mixtures, and cycling parameters used are specified in Tables S14 and S15. The resulting amplicons were subsequently purified with AMPure XP beads (Beckman Coulter, cat. no. A63881) and subjected to PCR barcoding using Illumina tag-specific primer pairs with unique sequence combinations for demultiplexing and sample identification. The cycling parameters, primers, and PCR mixtures used for the preparation of barcoded amplicons are indicated in Tables S15, S16, and S17, respectively. Next, the samples were again purified with AMPure XP beads and the concentrations of barcoded amplicons were determined by using the Qubit dsDNA HS assay kit (Thermo Fisher Scientific, cat. no. Q32854) and a Qubit2.0 fluorometer. Finally, purified amplicons were pooled in equal molar ratios and then subjected to Illumina MiSeq deep sequencing for retrieving 50,000 paired-end reads. Finally, after demultiplexing and adapter trimming of the paired-end MiSeq raw reads (R1 and R2 fastq files) with Cutadapt version 2.10,^61^ alignment of amplicon sequences to reference sequences was conducted by using the CRISPResso2 software.^62^ The scripts applied in each CRISPResso2 analyses round are available in Figures S12–S15.

### Statistical analysis

Statistical analyses were performed with the aid of the GraphPad Prism software (version 8.0.1) on datasets derived from independent biological replicates. Statistical significances were calculated with the tests indicated in the various figure legends. p values lower than 0.05 were considered statistically significant.

## Data availability

The data supporting the findings described in this study are available in the article and supplemental information. The next-generation sequencing read libraries are deposited at the NCBI Sequence Read Archive (SRA) database under BioProject ID PRJNA910947. Additional raw datasets are available from the corresponding author on reasonable request.

## Supplemental information

Document S1. Figures S1–S15 and Tables S1–S17

Document S2. Article plus supplemental information
