# Supplementary material for: High-capacity adenovector delivery of forced CRISPR-Cas9 heterodimers fosters precise chromosomal deletions in human cells
Source: Mol Ther Nucleic Acids. 2023 Feb 22;31:746–62. doi: 10.1016/j.omtn.2023.02.025 (PMC10020486; doi:10.1016/j.omtn.2023.02.025)
Supplement: Document S1. Figures S1–S15 and Tables S1–S17 [file mmc1.pdf]

OMTN, Volume *31*

Supplemental information

High-capacity adenovector delivery of forced CRISPR-Cas9 heterodimers fosters precise chromosomal deletions in human cells

Francesca Tasca, Marcella Brescia, Jin Liu, Josephine M. Janssen, Kamel Mamchaoui, and Manuel A.F.V. Gonçalves

Supplemental Tables

Table S1. Oligonucleotides used to assemble gRNA constructs.

Identifier and plasmid gRNA name	Oligo Codes	Oligos (5' → 3')
AZ43_gSp.16	#203	ACCGCTCGTGACCACCCTGACCTA
	#204	AAACTAGGTCAGGGTGGTCACGAG
BA21_gSp.3	#163	ACCGTAGGTCAGGGTGGTCACGA
	#164	AAACTCGTACCACCCTGACCTA
AV37_gSa.A	#407	CACCGCTTGTGCCCCAGGATGTTGC
	#408	AAACGCAACATCCTGGGGCACAAGC
BB10_gSa.G	#289	CACCGCAACATCCTGGGGCACAAGC
	#290	AAACGCTTGTGCCCCAGGATGTTGC
AM51_gSp.ISceI	#25	CACCGGTGAGCTCTTATTTGCGTAGCTAGCTGAC
	#26	AAACGTCAGCTAGCTACGCAAATAAGAGCTCAC

Table S2. Transfection scheme to generate the fluorescence-based reporter cell line HeLa.DsRed$^{TS.p(A).TS}$.

HeLa Cells	5 × 10^4 cells per 24-well	
	1.61 μl PEI / well (medium replaced at 6 h post-transfection)	
Plasmid	AQ02_Donor.AAVS1.CMV.TS.p(A).TS.DsRed	AV44_pCAG.Cas9^{D10A}.gRNA^{S1}
Size (bp)	9450	9450
1	205,3	214,7

Table S3. Transfection scheme to deliver gRNAs into H27 reporter cells.

H27	5 × 10^4 cells per 24-well
	1.15 μl PEI /well (medium replaced at 6 h post-transfection)
Plasmid	BA21_gSp.3
Size (bp)	3046
1	300

H27	5 × 10^4 cells per 24-well
	1.15 μl PEI /well (medium replaced at 6 h after post-transfection)
Plasmid	BB10_gSa.G
Size (bp)	2288
1	300

Table S4. Transfection scheme to deliver gRNAs into fluorescence-based reporter HeLa.DsRed$^{TS.pA.TS}$ cells.

HeLa.DsRed$^{TS.p(A).TS}$	5 × 10^4 cells per 24-well					
	1.61 μl PEI / well (medium replaced at 6 h post-transfection)					
Plasmid	AM51_gSp.ISceI	AZ43_gSp.16	BA21_gSp.3	AZ46_gSa-acceptor	AV73_gSa.A	BB10_gSa.G
Size (bp)	2056	3057	3046	2288	2288	2288
1	171,6			128,4		
2	171,6				128,4	
3		171,6		128,4		
4		171,6			128,4	
5			171,6	128,4		

6			171,6		128,4	
7	171,6					128,4
8		171,6				128,4
9			171,6			128,4

Table S5. Composition of mixtures used for qPCR.

Targets	Primer codes	Primers (5' → 3')	SYBR Green Master mix	Primers (μM)	Amplicons size (bp)
Ad5 Ψ	#95	CGGTGTACACAGGAAGTGACA	1×	0.2	123
	#1045	CAGATTTCACTTCCTCTTATTCAG			

Table S6. Thermocycler program used in qPCR amplification.

Steps	Temperatures	Times
Initial denaturation	95.0 ℃	5 min
Denaturation	95.0 ℃	10 sec
Annealing	60.0 ℃	30 sec
Elongation		
Plate read		
Cycles (Go to step 2)	40	
Melt curve analysis	65.0 ℃ to 95.0 ℃ (increase in 0.5 ℃ increments with a hold time of 5 sec for each read)	
Plate read		

Table S7. Transducing particle titers of AdVP stocks.

	qPCR titration method transduced vector genome copies per ml (GC ml^{-1})
AdVP.SaC9	1.20×10^{11}
AdVP.SpC9	1.70×10^{11}
AdVP.SaC9::SpC9	1.30×10^{10}
AdVP.SaC9::SpC9.dgRNA$^{\Delta51}$	9.82×10^{10}

Table S8. Gene knockout functional titers of AdVP stocks used in reporter HeLa.DsRed$^{TS.pA.TS}$ cell transduction experiments.

HeLa.DsRed$^{TS.pA.TS}$ (5×10^4 cells per 24-well)	Sp-based functional titer (Gene knockout units μl^{-1})	Sa-based functional titer (Gene knockout units μl^{-1})
AdVP.SaC9		$4{,}04\times10^5$
AdVP.SpC9	$9{,}97\times10^6$	
AdVP. SaC9::SpC9		$9{,}44\times10^5$
AdVP. SaC9::SpC9	$4{,}13\times10^6$	

Table S9. Antibodies used in confocal microscopy assays.

Primary antibody	Secondary antibody
Anti-Dystrophin (1:200; Abcam; Cat.No.: ab15277)	Alexa Fluor 488 goat anti-rabbit IgG (1:500; ThermoFisher; Cat.No.: A11034)
Anti-sarcomeric α-actinin (1:500; Sigma-Aldrich; Cat.No.: A7811)	Alexa Fluor 568 goat anti-mouse IgG (1:500; ThermoFisher; Cat.No.: A11004)
Anti-SpCas9 (1:500; Abcam; Cat. No.: ab191468)	Alexa Fluor 568 goat anti-mouse IgG (1:500; ThermoFisher; Cat.No.: A11004)
Anti-SaCas9 (1:1000; Diagenode; Cat.No.: C15310260)	Alexa Fluor 488 goat anti-rabbit IgG (1:500; ThermoFisher; Cat.No.: A11034)

Table S10. Antibodies used in the western blotting assays.

Primary antibody	Secondary antibody
Anti-Dystrophin (1:500; Abcam; Cat. No.: ab15277)	Goat anti-rabbit IgG-HRP (1:5000; Santa Cruz; Cat. No.: sc-2004)
Anti-sarcomeric α-actinin (1:10000; Sigma-Aldrich; Cat.No.: A7811)	Goat anti-mouse IgG-HRP (1:5000; Santa Cruz; Cat. No.: sc-2005)
Anti-SpCas9 (1:1000; Abcam; Cat. No.: ab191468)	Goat anti-mouse IgG-HRP (1:5000; Santa Cruz; Cat. No.: sc-2005)
Anti-GAPDH (1:1000; Millipore; Cat.No.: MAB374)	Goat anti-mouse IgG-HRP (1:5000; Santa Cruz; Cat. No.: sc-2005)
Anti-SaCas9 (1:15000; Diagenode; Cat.No.: C15310260)	Goat anti-rabbit IgG-HRP (1:5000; Santa Cruz; Cat. No.: sc-2004)

Table S11. Primer pair and PCR mixture composition for detecting targeted DNA cleavage.

Target	Primer code	Primers (5’ → 3’) / final concentrations (μM)	dNTPs (mM)	5X Phusion HF Buffer	Phusion HF DNA Polymerase (U μl^{-1})	Amplicon size (bp)
DMD Target site	#1674	TTCGGGTCACCTCTCACTCC / 0.5	0.2	1×	0.02	614 bp
	#1675	GGCTCCATCGTAAGCAAACC / 0.5				

Table S12. PCR cycling parameters for detecting targeted DNA cleavage.

Target	Initial denaturation	Denaturation	Annealing	elongation	Cycles	Final elongation
DMD Target site	98 °C	98 °C	66 °C	72 °C	29	72 ℃
	30 sec	10 sec	10 sec	15 sec		5 min

Table S13. Thermocycler program for generating heteroduplex substrates for T7EI.

Temperature	Time
95 °C	10 min
95 °C to 85 °C	-2.0 °C/s
85 °C	1 min
85 °C to 75 °C	-0.3 °C/s
75 °C	1 min
75 °C to 65 °C	-0.3 °C/s
65 °C	1 min
65 °C to 55 °C	-0.3 °C/s
55 °C	1 min
55 °C to 45 °C	-0.3 °C/s
45 °C	1 min
45 °C to 35 °C	-0.3 °C/s
35 °C	1 min
35 °C to 25 °C	-0.3 °C/s
25 °C	1 min
16 °C	Hold

Source: SURVEYOR Mutation Detection Kit (Transgenomic)

Table S14. Primers and PCR mixtures for NGS characterization of genome editing events.

**Target**	**Primer code**	**Primers (5' → 3') / final concentrations (µM)**	**dNTPs (mM)**	**5X Phusion HF Buffer**	**Phusion HF DNA Polymerase (U µl^{-1})**	**Amplicon size (bp)**
DMD Target site	#1720	GATGTGTATAAGAGACAGgtacctccaacatcaaggaag/ 0.5	0.2	1×	0.02	368 bp
	#1723	CGTGTGCTCTTCCGATCTgcatgagaatgagcaaaatcg/ 0.5				
LAMA2 Target site	#2064	GATGTGTATAAGAGACAGcccaagagtgagagtttgtct/ 0.5	0.2	1×	0.02	268 bp
	#2065	CGTGTGCTCTTCCGATCTgagacccaattgctgaaccat/ 0.5				
ZNF433 Target site	#2066	GATGTGTATAAGAGACAGcttaccacgggcattgcttt/ 0.5	0.2	1×	0.02	232 bp
	#2067	CGTGTGCTCTTCCGATCTgatttgccactccacactcc/ 0.5				
LYPD6 Target site	#2068	GATGTGTATAAGAGACAGcaagcatttgacgaagaagg/ 0.5	0.2	1×	0.02	286 bp
	#2069	CGTGTGCTCTTCCGATCTaatcccagctcaactaccta/ 0.5				
GABBR2 Target site	#2070	GATGTGTATAAGAGACAGggaattgaggctccaggaga/ 0.5	0.2	1×	0.02	281 bp
	#2071	CGTGTGCTCTTCCGATCTccgttctgtggacttgcttc/ 0.5				
MYOZ3 Target site	#2072	GATGTGTATAAGAGACAGtagaatgtcctgcgtcctgg/ 0.5	0.2	1×	0.02	308 bp
	#2073	CGTGTGCTCTTCCGATCTgcagcccacaacataggaag/ 0.5				
AL356153 Target site	#2074	GATGTGTATAAGAGACAGaatggttgaactgaagctca/ 0.5	0.2	1×	0.02	206 bp
	#2075	CGTGTGCTCTTCCGATCTtgctagggagtcatttccata/ 0.5				

Table S15. PCR cycling parameters for NGS assays to characterize genome editing events.

Target	Initial denaturation	Denaturation	Annealing	elongation	Cycles	Final elongation
DMD	98 °C	98 °C	67 °C	72 °C	29	72 ℃
	5 sec	10 sec	10 sec	15 sec		3 min
LAMA2	98 °C	98 °C	67 °C	72 °C	29	72 ℃
	5 sec	10 sec	10 sec	15 sec		3 min
ZNF433	98 °C	98 °C	67 °C	72 °C	29	72 ℃
	5 sec	10 sec	10 sec	15 sec		3 min
LYPD6	98 °C	98 °C	67 °C	72 °C	29	72 ℃
	5 sec	10 sec	10 sec	15 sec		3 min
GABBR2	98 °C	98 °C	67 °C	72 °C	29	72 ℃
	5 sec	10 sec	10 sec	15 sec		3 min
MYOZ3	98 °C	98 °C	67 °C	72 °C	29	72 ℃
	5 sec	10 sec	10 sec	15 sec		3 min
AL356153	98 °C	98 °C	67 °C	72 °C	29	72 ℃
	5 sec	10 sec	10 sec	15 sec		3 min
Barcoding	98 °C	98 °C	62 °C	72 °C	10	72 ℃
	30 sec	10 sec	10 sec	10 sec		5 min

Table S16. Barcode PCR primers used for NGS analyses to characterize genome editing events.

Primer code	Primers (5’ → 3’)
Fun-i501	AATGATACGGCGACCACCGAGATCTACACTAGATCGCTCGTCGGCAGCGTCAGATGTGTATAAGAGACA
Fun-i502	AATGATACGGCGACCACCGAGATCTACACCTCTCTATTCGTCGGCAGCGTCAGATGTGTATAAGAGACAG
Fun-i503	AATGATACGGCGACCACCGAGATCTACACTATCCTCTTCGTCGGCAGCGTCAGATGTGTATAAGAGACAG
Fun-i504	AATGATACGGCGACCACCGAGATCTACACTATCCTCTTCGTCGGCAGCGTCAGATGTGTATAAGAGACAG
Fun-i505	AATGATACGGCGACCACCGAGATCTACACAGAGTAGATCGTCGGCAGCGTCAGATGTGTATAAGAGACAG
Fun-i506	AATGATACGGCGACCACCGAGATCTACACGTAAGGAGTCGTCGGCAGCGTCAGATGTGTATAAGAGACAG
Fun-i507	AATGATACGGCGACCACCGAGATCTACACACTGCATATCGTCGGCAGCGTCAGATGTGTATAAGAGACAG
Fun-i508	AATGATACGGCGACCACCGAGATCTACACAAGGAGTATCGTCGGCAGCGTCAGATGTGTATAAGAGACAG
Fun-i517	AATGATACGGCGACCACCGAGATCTACACGCGTAAGATCGTCGGCAGCGTCAGATGTGTATAAGAGACAG
Fun-i501D	AATGATACGGCGACCACCGAGATCTACACTATAGCCTTCGTCGGCAGCGTCAGATGTGTATAAGAGACAG
Fun-i502D	AATGATACGGCGACCACCGAGATCTACACATAGAGGCTCGTCGGCAGCGTCAGATGTGTATAAGAGACAG
Fun-i503D	AATGATACGGCGACCACCGAGATCTACACCCTATCCTTCGTCGGCAGCGTCAGATGTGTATAAGAGACAG
Fun-i504D	AATGATACGGCGACCACCGAGATCTACACGGCTCTGATCGTCGGCAGCGTCAGATGTGTATAAGAGACAG
Fun-i505D	AATGATACGGCGACCACCGAGATCTACACAGGCGAAGTCGTCGGCAGCGTCAGATGTGTATAAGAGACA
Fun-i506D	AATGATACGGCGACCACCGAGATCTACACTAATCTTATCGTCGGCAGCGTCAGATGTGTATAAGAGACAG
Fun-i507D	AATGATACGGCGACCACCGAGATCTACACCAGGACGTTCGTCGGCAGCGTCAGATGTGTATAAGAGACAG
Fun-i508D	AATGATACGGCGACCACCGAGATCTACACGTACTGACTCGTCGGCAGCGTCAGATGTGTATAAGAGACAG

Fun-i701	CAAGCAGAAGACGGCATACGAGATTCGCCTTAGTGACTGGAGTTCAGACGTGTGCTCTTCCGATCT
Fun-i702	CAAGCAGAAGACGGCATACGAGATCTAGTACGGTGACTGGAGTTCAGACGTGTGCTCTTCCGATCT
Fun-i703	CAAGCAGAAGACGGCATACGAGATTTCTGCCTGTGACTGGAGTTCAGACGTGTGCTCTTCCGATCT
Fun-i704	CAAGCAGAAGACGGCATACGAGATGCTCAGGAGTGACTGGAGTTCAGACGTGTGCTCTTCCGATCT
Fun-i705	CAAGCAGAAGACGGCATACGAGATAGGAGTCCGTGACTGGAGTTCAGACGTGTGCTCTTCCGATCT
Fun-i706	CAAGCAGAAGACGGCATACGAGATCATGCCTAGTGACTGGAGTTCAGACGTGTGCTCTTCCGATCT
Fun-i707	CAAGCAGAAGACGGCATACGAGATGTAGAGAGGTGACTGGAGTTCAGACGTGTGCTCTTCCGATCT
Fun-i708	CAAGCAGAAGACGGCATACGAGATCCTCTCTGGTGACTGGAGTTCAGACGTGTGCTCTTCCGATCT
Fun-i709	CAAGCAGAAGACGGCATACGAGATAGCGTAGCGTGACTGGAGTTCAGACGTGTGCTCTTCCGATCT
Fun-i710	CAAGCAGAAGACGGCATACGAGATCAGCCTCGGTGACTGGAGTTCAGACGTGTGCTCTTCCGATCT
Fun-i711	CAAGCAGAAGACGGCATACGAGATTGCCTCTTGTGACTGGAGTTCAGACGTGTGCTCTTCCGATCT
Fun-i712	CAAGCAGAAGACGGCATACGAGATTCCTCTACGTGACTGGAGTTCAGACGTGTGCTCTTCCGATCT

Table S17. Composition of PCR mixtures used for the barcoding of NGS amplicons.

**Component**	**Volume**	**Final Concentration**
5× Phusion HF Buffer	4 µl	1×
dNTPs (2.5 mM each)	1.2 µl	0.15 mM (each)
PCR Grade Water	11.6 µl	-
Index primer p5-XX (5 µM)	1 µl	0.25 µM
Index primer p7-XX (5 µM)	1 µl	0.25 µM
Purified PCR product	1 µl	-
Phusion DNA Polymerase (2 U µl^{-1})	0.2 µl	0.02 U µl^{-1}
Total reaction volume	20 µl	-

Supplemental Figures

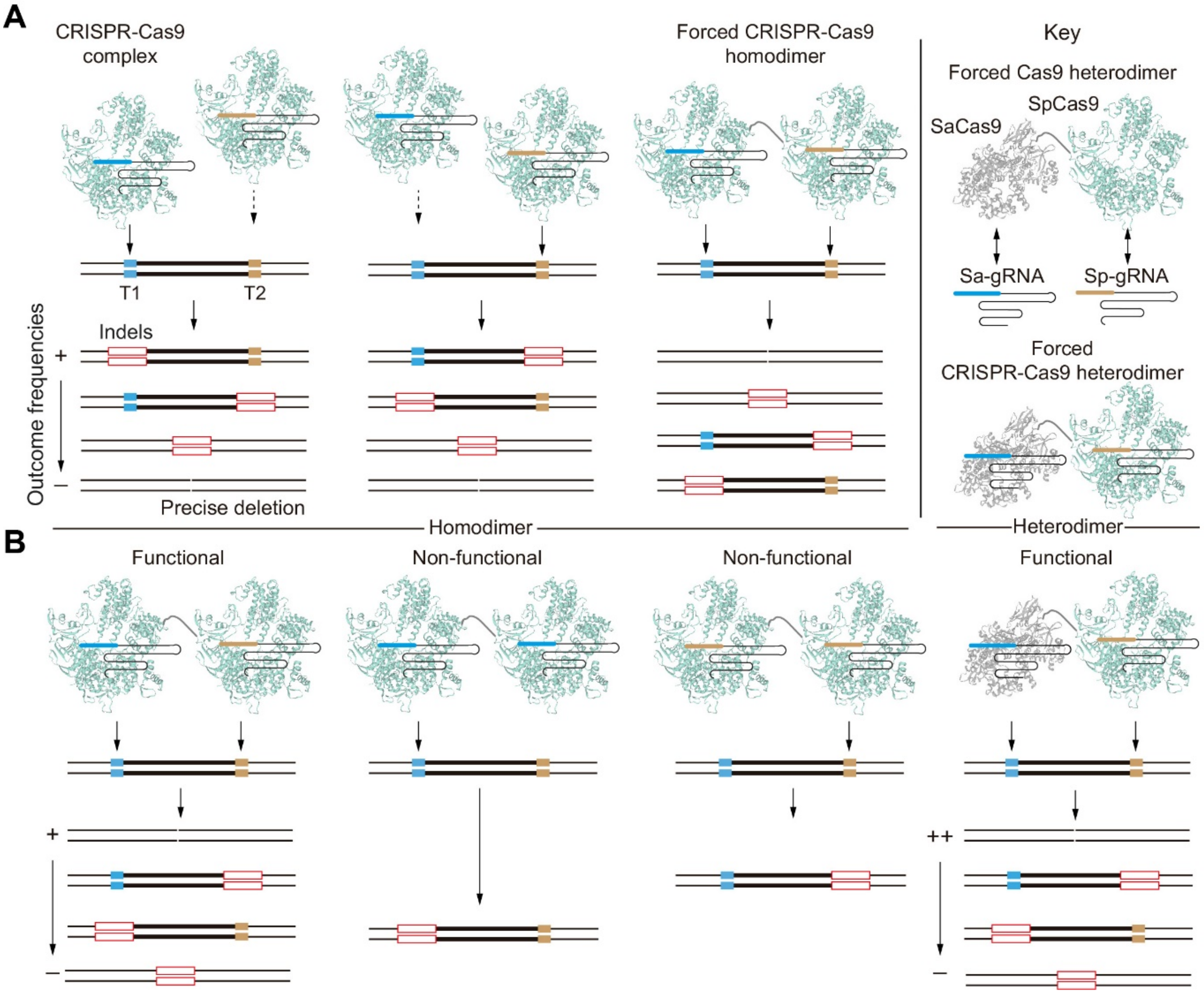

Figure S1. Rationale for multiplexing genome editing based on forced CRISPR-Cas9 heterodimers. (**A**) Multiplexing genome editing with conventional versus forced CRISPR-Cas9 complexes. Independent and linked monotypic CRISPR-Cas9 complexes (forced CRISPR-Cas9 homodimers) engage target sequences in an uncoordinated and coordinated fashion yielding low and high frequencies, respectively, of precise target DNA deletions. (**B**) Multiplexing genome editing with homodimeric versus heterodimeric CRISPR-Cas9 complexes. Forced Cas9 heterodimers and orthogonal gRNA-Cas9 interactions assure the exclusive assembly of functional CRISPR-Cas9 multiplexes further favoring precise deletions over unintended genome editing outcomes.

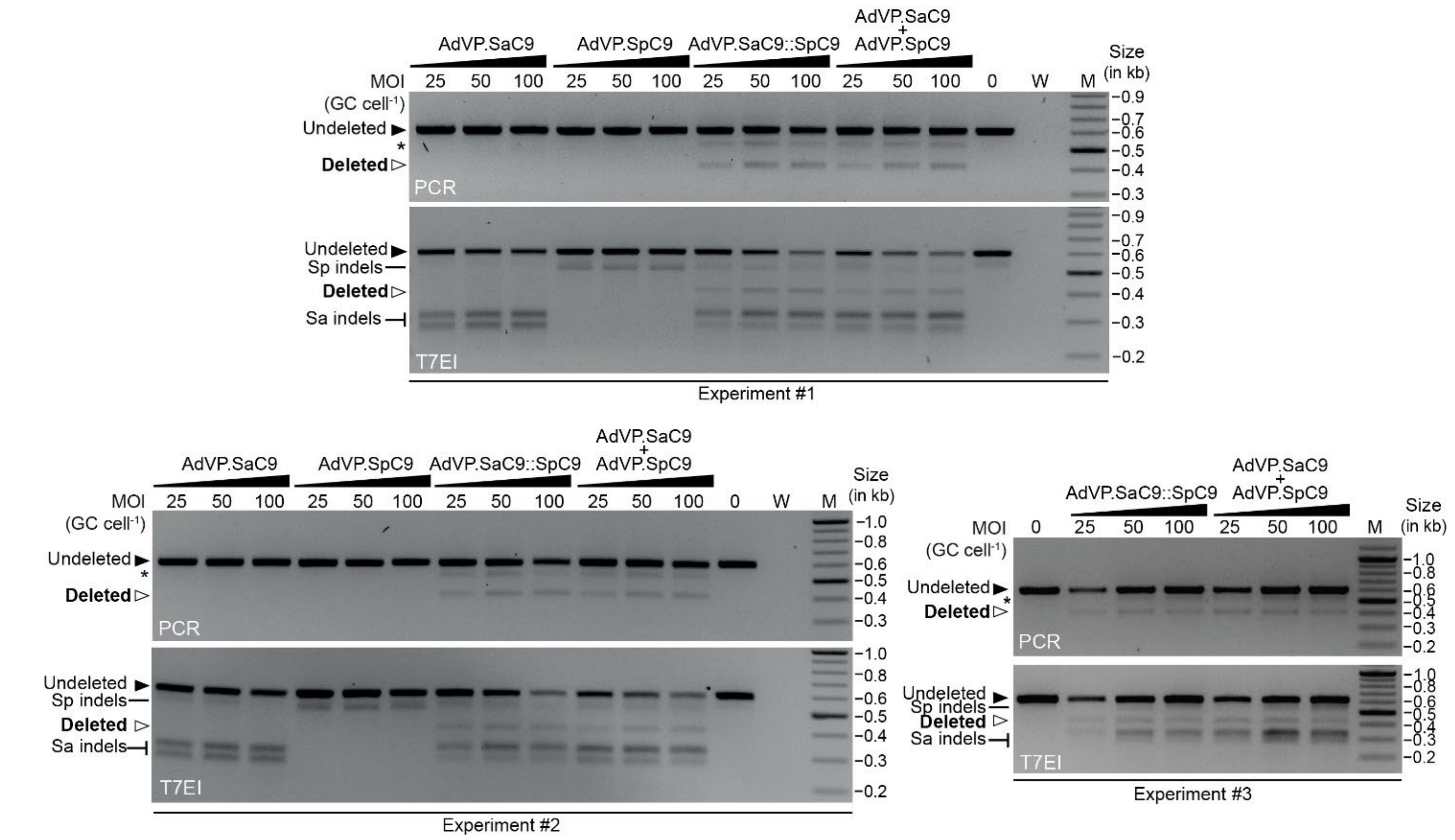

Figure S2. Testing genome editing upon AdVP delivery of unlinked and forced Cas9 heterodimers. PCR genotyping assays on DMD.1 myoblasts stably expressing gSa^{IN50} and gSp^{EX51} (DMD.1.$dgRNA^{\Delta 51}$) transduced with AdVP.SaC9::SpC9 or with AdVP.SaC9 and AdVP.SpC9 at the indicated total MOIs. Mock-transduced cells were used as negative controls. Solid and open arrowheads point to amplicons derived from undeleted and deleted *DMD* alleles. Line and crossed line indicate DNA species derived from T7EI digestion of amplicons with NHEJ-derived indels at SpCas9 and SaCas9 target sites, respectively. The asterisks mark the position of heteroduplexes formed during PCR by the hybridization of DNA strands from the upper and lower products. MOI, multiplicity of infection; GC $cell^{-1}$, genome copies per cell; Marker, GeneRuler DNA Ladder Mix.

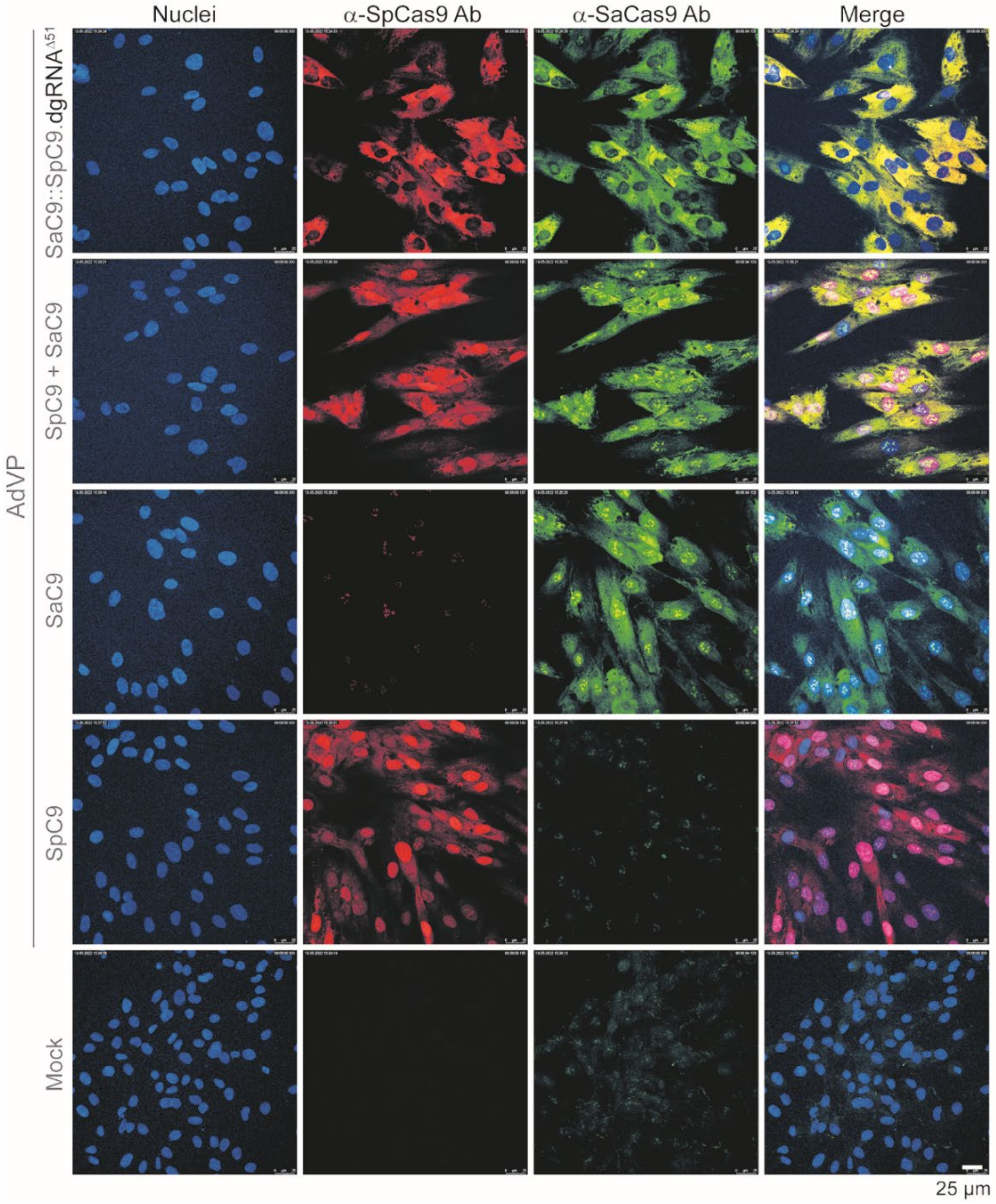

Figure S3. Assessing SaCas9 and SpCas9 synthesis upon AdVP delivery of unlinked and forced Cas9 heterodimers. Detection of SpCas9 and SaCas9 expression by confocal immunofluorescence microscopy in wild-type human myoblasts transduced with AdVP.SaC9::SpC9.dgRNA$^{\Delta51}$, AdVP.SaC9 and AdVP.SpC9 at an MOI of 400 GC cell^{-1} at 2 days post-transduction. Mock-transduced DMD cells served as negative controls. Nuclei were labelled with DAPI. MOI, multiplicity of infection; GC cell^{-1}, genome copies per cell.

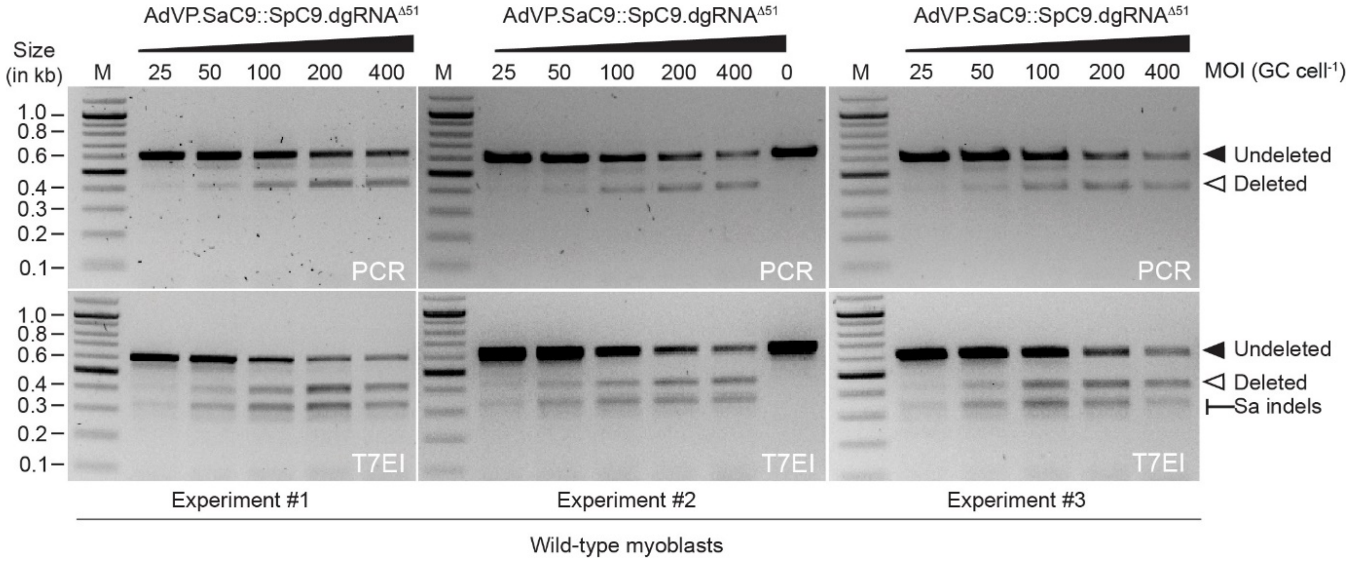

Figure S4. Testing genome editing upon all-in-one AdVP delivery of forced CRISPR-Cas9 heterodimers. PCR genotyping assays on wild-type myoblasts transduced with AdVP.SaC9::SpC9.dgRNA$^{\Delta51}$ at the indicated MOI. Mock-transduced cells were used as negative controls. Solid and open arrowheads point to amplicons derived from undeleted and deleted *DMD* alleles. Crossed line indicate DNA species derived from T7EI digestion of amplicons with NHEJ-derived indels at the SaCas9 target site. The asterisks mark the position of heteroduplexes formed during PCR by the hybridization of DNA strands from the upper and lower products. MOI, multiplicity of infection; GC cell^{-1}, genome copies per cell; Marker, GeneRuler DNA Ladder Mix.

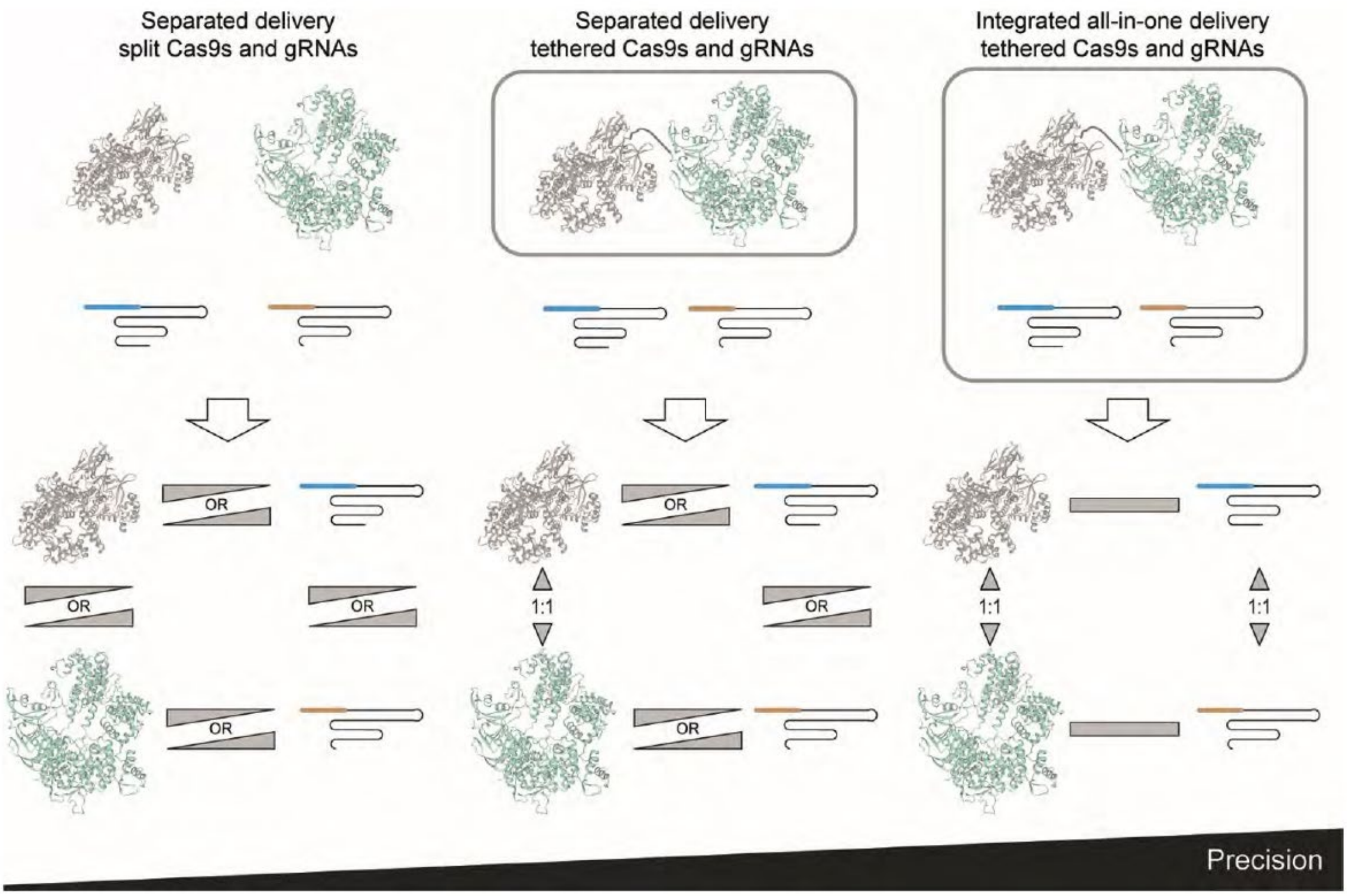

Figure S5. Working model on the role for balanced assembly of multiplexing CRISPR components in maximizing genome editing precision. The synchronous binding and coordinated activity of multiplexing CRISPR-Cas9 complexes at bipartite target sequences fosters precision genome editing. Equilibrated proportions between Cas9 and gRNA components (theoretical optimum 1:1) favor timely assembly of CRISPR-Cas9 multiplexes with functional Cas9:gRNA subunits. The likelihood of achieving equilibrated proportions among members of each CRISPR-Cas9 subunit increases gradually from experimental setups involving separate delivery of untethered Cas9 proteins and dual gRNAs; separate delivery of tethered Cas9 proteins and dual gRNAs; to integrated all-in-one delivery of tethered Cas9 proteins and dual gRNAs.

Supplemental Information

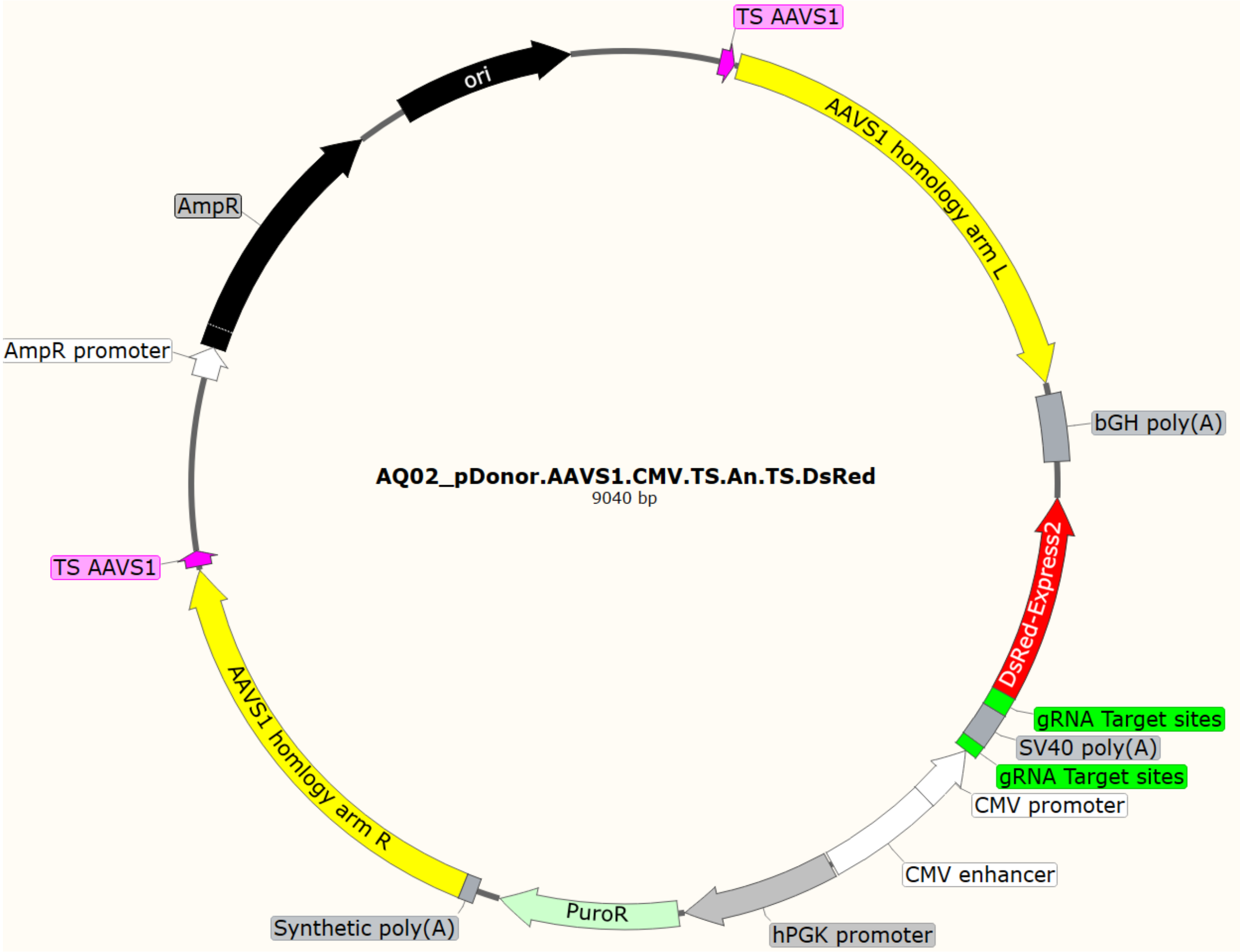

AGAGCGCCCAATACGCAAACCGCCTCTCCCCGCGCGTTGGCCGATTCATTAATGCAGCTGGCACGACAGGTTTCC
CGACTGGAAAGCGGGCAGTGAGCGCAACGCAATTAATGTGAGTTAGCTCACTCATTAGGCACCCCAGGCTTTACA
CTTTATGCTTCCGGCTCGTATGTTGTGTGGAATTGTGAGCGGATAACAATTTCACACAGGAAACAGCTATGACCA
TGATTACGCCAAGCTAATTCGAGCTCGGTACCTCGCGAATGCATCTAGCTAGATATCTAGGGATAACAGGGTAAT
GTCGAGGCCGGATTTAAATCTGTCCCCTCCACCCCACAGTGGGGCCACTAGGGACAGGATTGGTGACAGAGCGGC
CGCTGATGCCGAGCTCGACCCCGCCGCGCCGCCGCCCGCCCGCGCCGTGCTGGACTCCACCAACGCCGACGGTAT
CAGCGCCCTGCACCAGGTCAGCGCCCCCCGCCCGGCGTCTCCCGGGGCCAGGTCCACCCTCTGCTGCGCCACCTG
GGGCATCCTCCTTCCCCGTTGCCAGTCTCGATCCGCCCCGTCGTTCCTGGCCCTGGGCTTTGCCACCCTATGCTG
ACACCCCGTCCCAGTCCCCCTTACCATTCCCCTTCGACCACCCCACTTCCGAATTGGAGCCGCTTCAACTGGCCC
TGGGCTTAGCCACTCTGTGCTGACCACTCTGCCCCAGGCCTCCTTACCATTCCCCTTCGACCTACTCTCTTCCGC
ATTGGAGTCGCTTTAACTGGCCCTGGCTTTGGCAGCCTGTGCTGACCCATGCAGTCCTCCTTACCATCCCTCCCT
CGACTTCCCCTCTTCCGATGTTGAGCCCCTCCAGCCGGTCCTGGACTTTGTCTCCTTCCCTGCCCTGCCCTCTCC
TGAACCTGAGCCAGCTCCCATAGCTCAGTCTGGTCTATCTGCCTGGCCCTGGCCATTGTCACTTTGCGCTGCCCT
CCTCTCGCCCCCGAGTGCCCTTGCTGTGCCGCCGGAACTCTGCCCTCTAACGCTGCCGTCTCTCTCCTGAGTCCG
GACCACTTTGAGCTCTACTGGCTTCTGCGCCGCCTCTGGCCCACTGTTTCCCCTTCCCAGGCAGGTCCTGCTTTC
TCTGACCTGCATTCTCTCCCCTGGGCCTGTGCCGCTTTCTGTCTGCAGCTTGTGGCCTGGGTCACCTCTACGGCT
GGCCCAGATCCTTCCCTGCCGCCTCCTTCAGGTTCCGTCTTCCTCCACTCCCTCTTCCCCTTGCTCTCTGCTGTG
TTGCTGCCCAAGGATGCTCTTTCCGGAGCACTTCCTTCTCGGCGCTGCACCACGTGATGTCCTCTGAGCGGATCC
TCCCCGTGTCTGGGTCCTCTCCGGGCATCTCTCCTCCCTCACCCAACCCCATGCCGTCTTCACTCGCTGGGTTCC
CTTTTCCTTCTCCTTCTGGGGCCTGTGCCATCTCTCGTTTCTTAGGATGGCCTTCTCCGACGGATGTCTCCCTTG
CGTCCCGCCTCCCCTTCTTGTAGGCCTGCATCATCACCGTTTTTCTGGACAACCCCAAAGTACCCCGTCTCCCTG
GCTTTAGCCACCTCTCCATCCTCTTGCTTTCTTTGCCTGGACACCCCGTTCTCCTGTGGATTCGGGTCACCTCTC
ACTCCTTTCATTTGGGCAGCTCCCCTACCCCCCTTACCTCTCTAGTCTGTGCTAGCTCTTCCAGCCCCCTGTCAT
GGCATCTTCCAGGGGTCCGAGAGCTCAGCTAGTCTTCTTCCTCCAACCCGGGCCCCTATGTCCACTTCAGGACAG

CATGTTTGCTGCCTCCAGGGATCCTGTGTCCCCGAGCTGGGACCACCTTATATTCCCAGGGCCGGTTAATGTGGC
TCTGGTTCTGGGTACTTTTATCTGTCCCCTCCACCCCACAGTGGGGCCGCGCGCGACTAGTGTCCTGGTTCTTTC
CGCCTCAGAAGCCATAGAGCCCACCGCATCCCCAGCATGCCTGCTATTGTCTTCCCAATCCTCCCCCTTGCTGTC
CTGCCCCACCCCACCCCCCAGAATAGAATGACACCTACTCAGACAATGCGATGCAATTTCCTCATTTTATTAGGA
AAGGACAGTGGGAGTGGCACCTTCCAGGGTCAAGGAAGGCACGGGGGAGGGGCAAACAACAGATGGCTGGCAACT
AGAAGGCACAGTCGAGGCTGATCAGCGGGTTTGACTGCAGAGGCCTGCATGCAAGCTATCGATCGCGCGCAGATC
TACAGCTGCCTTGTAAGTCATTGGTCTTAAAGGTACCGAGCTCGAATTTTAAGGCCGCTACTGGAACAGGTGGTG
GCGGGCCTCGGCGCGCTCGTACTGCTCCACCACGGTGTAGTCCTCGTTGTGGAGGTGATGTCCAGCTTGGAGTC
CACGTAGTAGTAGCCGGGCAGCTTCACGGCTTCTTGGCCATGTAGATTGACTTGAACTCCACCAGGTAGTGGCC
GCCGCCCTTCAGCTTCAGCGCCTTGTGGATCTCGCCCTTCAGCACGCCGTCGCGGGGGTACAGGCGCTCGGTGGA
GGGCTCCCAGCCCAGAGTCTTCTTCTGCATTACGGGGCCGTCGGAGGGGAAGTTCACGCCGATGAACTTCACGTG
GTAGATGAAGGTGCCGTCCTGCAGGGAGGAGTCCTGGGTCACGGTCACCACGCCGCCGTCCTCGAAGTTCATCAC
GCGCTCCCACTTGAAGCCCTCGGGGAAGGACAGCTTCTTGTAGTCGGGGATGTCGGCGGGGTGCTTCACGTACAC
CTTGGAGCCGTACTGGAACTGGGGGGACAGGATGTCCCAGGCGAAGGGCAGGGGGCCGCCCTTGGTCACCTGCAG
CTTGGCGGTCTGGGTGCCCTCGTAGGGCTTGCCCTCGCCCTCGCCCTCGATCTCGAACTCGTGGCCGTTCACGGA
GCCCTCCATGTGCACCTTGAAGCGCATGAAGGGCTTGATGACGTTCTCAGTGCTATCCATGGTGGCGACCGGTAC
TCCAGCAACATCCTGGGGCACAAGCTGGAGTTGTACATTACTTATTTAAACTTGTTTATTGCAGCTTATAATGGT
TACAAATAAAGCAATAGCATCACAAATTTCACAAATAAAGCATTTTTTTCACTGCATTCTAGTTGTGGTTTGTCC
AAACTCATCAATGTATCTTATCATTCTAGAGCCGTAGGTCAGGGTGGTCACGAGGGTTGAATTCGAGCTCTGCTT
ATATAGACCTCCCACCGTACACGCCTACCGCCCATTTGCTTCAATGGGGCGGAGTTGTTACGACATTTTGGAAAG
TCCCGTTGATTTTGGTGCCAAAACAAACTCCCATTGACGTCAATGGGGTGGAGACTTGGAAATCCCCGTGAGTCA
AACCGCTATCCACGCCCATTGATGTACTGCCAAAACCGCATCACCATGGTAATAGCGATGACTAATACGTAGATG
TACTGCCAAGTAGGAAAGTCCCATAAGGTCATGTACTGGGCATAATGCCAGGCGGGCCATTTACCGTCATTGACG
TCAATAGGGGGCGTACTTGGCATATGATACACTTGATGTACTGCCAAGTGGGCAGTTTACCGTAAATACTCCACC
CATTGACGTCAATGGAAAGTCCCTATTGGCGTTACTATGGGAACATACGTCATTATTGACGTCAATGGGCGGGGG
TCGTTGGGCGGTCAGCCAGGCGGGCCATTTACCGTAAGTTATGTAACGCGGAACTCCATATATGGGCTATGAACT
AATGACCCCGTAATTGATTACTATTAATAACTAGTCAATAATCAATGTCAAATCTCGAGGGGGTTGGGGTTGCGC
CTTTTCCAAGGCAGCCCTGGGTTTGCGCAGGGACGCGGCTGCTCTGGGCGTGGTTCCGGGAAACGCAGCGGCGCC
GACCCTGGGTCTCGCACATTCTTCACGTCCGTTCGCAGCGTCACCCGGATCTTCGCCGCTACCCTTGTGGGCCCC
CCGGCGACGCTTCCTGCTCCGCCCCTAAGTCGGGAAGGTTCCTTGCGGTTCGCGGCGTGCCGGACGTGACAAACG
GAAGCCGCACGTCTCACTAGTACCCTCGCAGACGGACAGCGCCAGGGAGCAATGGCAGCGCGCCGACCGCGATGG
GCTGTGGCCAATAGCGGCTGCTCAGCAGGGCGCGCCGAGAGCAGCGGCCGGGAAGGGGCGGTGCGGGAGGCGGGG
TGTGGGGCGGTAGTGTGGGCCCTGTTCCTGCCCGCGCGGTGTTCCGCATTCTGCAAGCCTCCGGAGCGCACGTCG
GCAGTCGGCTCCCTCGTTGACCGAATCACCGACCTCTCTCCCCAGGGGGATCCACCGGAGCTTACCATGACCGAG
TACAAGCCCACGGTGCGCCTCGCCACCCGCGACGACGTCCCCAGGGCCGTACGCACCCTCGCCGCCGCGTTCGCC
GACTACCCCGCCACGCGCCACACCGTCGATCCGGACCGCCACATCGAGCGGGTCACCGAGCTGCAAGAACTCTTC
CTCACGCGCGTCGGGCTCGACATCGGCAAGGTGTGGGTCGCGGACGACGGCGCCGCGGTGGCGGTCTGGACCACG
CCGGAGAGCGTCGAAGCGGGGGCGGTGTTCGCCGAGATCGGCCCGCGCATGGCCGAGTTGAGCGGTTCCCGGCTG
GCCGCGCAGCAACAGATGGAAGGCCTCCTGGCGCCGCACCGGCCCAAGGAGCCCGCGTGGTTCCTGGCCACCGTC
GGCGTCTCGCCCGACCACCAGGGCAAGGGTCTGGGCAGCGCCGTCGTGCTCCCCGGAGTGGAGGCGGCCGAGCGC
GCCGGGGTGCCCGCCTTCCTGGAGACCTCCGCGCCCCGCAACCTCCCCTTCTACGAGCGGCTCGGCTTCACCGTC
ACCGCCGACGTCGAGGTGCCCGAAGGACCGCGCACCTGGTGCATGACCCGCAAGCCCGGTGCCTGACGCCCGCCC
CACGACCCGCAGCGCCCGACCGAAAGGAGCGCACGACCCCATGCATCGGTACCGCGGCCAATTCACGCGTAATAA
AATATCTTTATTTTCATTACATCTGTGTGTTGGTTTTTTGTGTGTAGGGACAGGATTGGTGACAGAAAAGCCCCA
TCCTTAGGCCTCCTCCTTCCTAGTCTCCTGATATTGGGTCTAACCCCCACCTCCTGTTAGGCAGATTCCTTATCT
GGTGACACACCCCCATTTCCTGGAGCCATCTCTCTCCTTGCCAGAACCTCTAAGGTTTGCTTACGATGGAGCCAG
AGAGGATCCTGGGAGGGAGAGCTTGGCAGGGGGTGGGAGGGAAGGGGGGGATGCGTGACCTGCCCGGTTCTCAGT
GGCCACCCTGCGCTACCCTCTCCCAGAACCTGAGCTGCTCTGACGCGGCCGTCTGGTGCGTTTCACTGATCCTGG
TGCTGCAGCTTCCTTACACTTCCCAAGAGGAGAAGCAGTTTGGAAAAACAAAATCAGAATAAGTTGGTCCTGAGT
TCTAACTTTGGCTCTTCACCTTTCTAGTCCCCAATTTATATTGTTCCTCCGTGCGTCAGTTTTACCTGTGAGATA
AGGCCAGTAGCCAGCCCCGTCCTGGCAGGGCTGTGGTGAGGAGGGGGGTGTCCGTGTGGAAAACTCCCTTTGTGA
GAATGGTGCGTCCTAGGTGTTCACCAGGTCGTGGCCGCCTCTACTCCCTTTCTCTTTCTCCATCCTTCTTTCCTT
AAAGAGTCCCCAGTGCTATCTGGGACATATTCCTCCGCCCAGAGCAGGGTCCCGCTTCCCTAAGGCCCTGCTCTG
GGCTTCTGGGTTTGAGTCCTTGGCAAGCCCAGGAGAGGCGCTCAGGCTTCCCTGTCCCCCTTCCTCGTCCACCAT
CTCATGCCCCTGGCTCTCCTGCCCCTTCCCTACAGGGGTTCCTGGCTCTGCTCTTCAGACTGAGCCCCGTTCCCC
TGCATCCCCGTTCCCCTGCATCCCCCTTCCCCTGCATCCCCCAGAGGCCCCAGGCCACCTACTTGGCCTGGACCC
CACGAGAGGCCACCCCAGCCCTGTCTACCAGGCTGCCTTTTGGGTGGATTCTCCTCCAACTGTGGGGTGACTGCT
TGGCAAACTCACTCTTCGGGGTATCCCAGGAGGCCTGGAGCATTGGGGTGGCTGGGGTTCAGAGAGGAGGGATT
CCCTTCTCAGGTTACGTGGCCAAGAAGCAGGGGAGCTGGGTTTGGGTCAGGTCTGGGTGTGGGGTGACCAGCTTA
TGCTGTTTGCCCAGGACAGCCTAGTTTTAGCACTGAAACCCTCAGTCCTAGGAAAACAGGATGGTTGGTCACTG
TCTCTGGGTGACTCTTGATTCCCGGCCAGTTTCTCCACCTGGGGCTGTGTTTCTCGTCCTGCATCCTTCTCCAGG

CAGGTCCCCAAGCATCGCCCCCCTGCTGTGGCTGTTCCCAAGTTCTTAGGGTACCCCACGTGGGTTTATCAACCA
CTTGGTGAGGCTGGTACCCTGCCCCCATTCCTGCACCCCAATTGGCCGCGCCGGCGCTGTCCCCTCCACCCCACA
GTGGGGCCACTAGGGACAGGATTGGTGACAGAGTTTAAACTAGGGATAACAGGGTAATGTCGAGGCCGGGATATC
GGATCCCGGGCCCGTCGACTGCAGAGGCCTGCATGCAAGCTAATTCACTGGCCGTCGTTTTACAACGTCGTGACT
GGGAAAACCCTGGCGTTACCCAACTTAATCGCCTTGCAGCACATCCCCCTTTCGCCAGCTGGCGTAATAGCGAAG
AGGCCCGCACCGATCGCCCTTCCCAACAGTTGCGCAGCCTGAATGGCGAATGGCGCCTGATGCGGTATTTTCTCC
TTACGCATCTGTGCGGTATTTCACACCGCATATGGTGCACTCTCAGTACAATCTGCTCTGATGCCGCATAGTTAA
GCCAGCCCCGACACCCGCCAACACCCGCTGACGCGCCCTGACGGGCTTGTCTGCTCCCGGCATCCGCTTACAGAC
AAGCTGTGACCGTCTCCGGGAGCTGCATGTGTCAGAGGTTTTCACCGTCATCACCGAAACGCGCGAGACGAAAGG
GCCTCGTGATACGCCTATTTTTATAGGTTAATGTCATGATAATAATGGTTTCTTAGACGTCAGGTGGCACTTTTC
GGGGAAATGTGCGCGGAACCCCTATTTGTTTATTTTTCTAAATACATTCAAATATGTATCCGCTCATGAGACAAT
AACCCTGATAAATGCTTCAATAATATTGAAAAAGGAAGAGTATGAGTATTCAACATTTCCGTGTCGCCCTTATTC
CCTTTTTTGCGGCATTTTGCCTTCCTGTTTTTGCTCACCCAGAAACGCTGGTGAAAGTAAAAGATGCTGAAGATC
AGTTGGGTGCACGAGTGGGTTACATCGAACTGGATCTCAACAGCGGTAAGATCCTTGAGAGTTTTCGCCCCGAAG
AACGTTTTCCAATGATGAGCACTTTTAAAGTTCTGCTATGTGGCGCGGTATTATCCCGTATTGACGCCGGGCAAG
AGCAACTCGGTCGCCGCATACACTATTCTCAGAATGACTTGGTTGAGTACTCACCAGTCACAGAAAAGCATCTTA
CGGATGGCATGACAGTAAGAGAATTATGCAGTGCTGCCATAACCATGAGTGATAACACTGCGGCCAACTTACTTC
TGACAACGATCGGAGGACCGAAGGAGCTAACCGCTTTTTTGCACAACATGGGGGATCATGTAACTCGCCTTGATC
GTTGGGAACCGGAGCTGAATGAAGCCATACCAAACGACGAGCGTGACACCACGATGCCTGTAGCAATGGCAACAA
CGTTGCGCAAACTATTAACTGGCGAACTACTTACTCTAGCTTCCCGGCAACAATTAATAGACTGGATGGAGGCGG
ATAAAGTTGCAGGACCACTTCTGCGCTCGGCCCTTCCGGCTGGCTGGTTTATTGCTGATAAATCTGGAGCCGGTG
AGCGTGGGTCTCGCGGTATCATTGCAGCACTGGGGCCAGATGGTAAGCCCTCCCGTATCGTAGTTATCTACACGA
CGGGGAGTCAGGCAACTATGGATGAACGAAATAGACAGATCGCTGAGATAGGTGCCTCACTGATTAAGCATTGGT
AACTGTCAGACCAAGTTTACTCATATATACTTTAGATTGATTTAAAACTTCATTTTTAATTTAAAAGGATCTAGG
TGAAGATCCTTTTTGATAATCTCATGACCAAAATCCCTTAACGTGAGTTTTCGTTCCACTGAGCGTCAGACCCCG
TAGAAAAGATCAAAGGATCTTCTTGAGATCCTTTTTTTCTGCGCGTAATCTGCTGCTTGCAAACAAAAAAACCAC
CGCTACCAGCGGTGGTTTGTTTGCCGGATCAAGAGCTACCAACTCTTTTTCCGAAGGTAACTGGCTTCAGCAGAG
CGCAGATACCAAATACTGTTCTTCTAGTGTAGCCGTAGTTAGGCCACCACTTCAAGAACTCTGTAGCACCGCCTA
CATACCTCGCTCTGCTAATCCTGTTACCAGTGGCTGCTGCCAGTGGCGATAAGTCGTGTCTTACCGGGTTGGACT
CAAGACGATAGTTACCGGATAAGGCGCAGCGGTCGGGCTGAACGGGGGGTTCGTGCACACAGCCCAGCTTGGAGC
GAACGACCTACACCGAACTGAGATACCTACAGCGTGAGCTATGAGAAAGCGCCACGCTTCCCGAAGGGAGAAAGG
CGGACAGGTATCCGGTAAGCGGCAGGGTCGGAACAGGAGAGCGCACGAGGGAGCTTCCAGGGGGAAACGCCTGGT
ATCTTTATAGTCCTGTCGGGTTTCGCCACCTCTGACTTGAGCGTCGATTTTTGTGATGCTCGTCAGGGGGGCGGA
GCCTATGGAAAAACGCCAGCAACGCGGCCTTTTTACGGTTCCTGGCCTTTTGCTGGCCTTTTGCTCACATGTTCT
TTCCTGCGTTATCCCCTGATTCTGTGGATAACCGTATTACCGCCTTTGAGTGAGCTGATACCGCTCGCCGCAGCC
GAACGACCGAGCGCAGCGAGTCAGTGAGCGAGGAAGCGGA

Figure S6. Map and sequence of conditional reporter plasmid AQ02_pDonor.AAVS1.CMV.TS.An.TS.DsRed. *AAVS1* homology arm L and R, sequences homologous to the human *AAVS1* locus flanking the Cas9:gRNA^{S1} target site; TS AAVS1, target sequence of Cas9:gRNA^{S1} complexes; CMV enhancer/promoter, human cytomegalovirus (CMV) *immediate-early* gene regulatory sequences; DsRed-Express2, red fluorescent protein gene; PuroR, PuroR, gene coding for puromycin N-acetyltransferase conferring resistance to puromycin; hPGK promoter, human *phosphoglycerate kinase 1* regulatory sequences; bGH poly(A), bovine growth hormone polyadenylation signal; SV40 poly(A), polyadenylation signal from the simian virus 40 large T antigen. The different features are color coded in the plasmid map and respective nucleotide sequence.

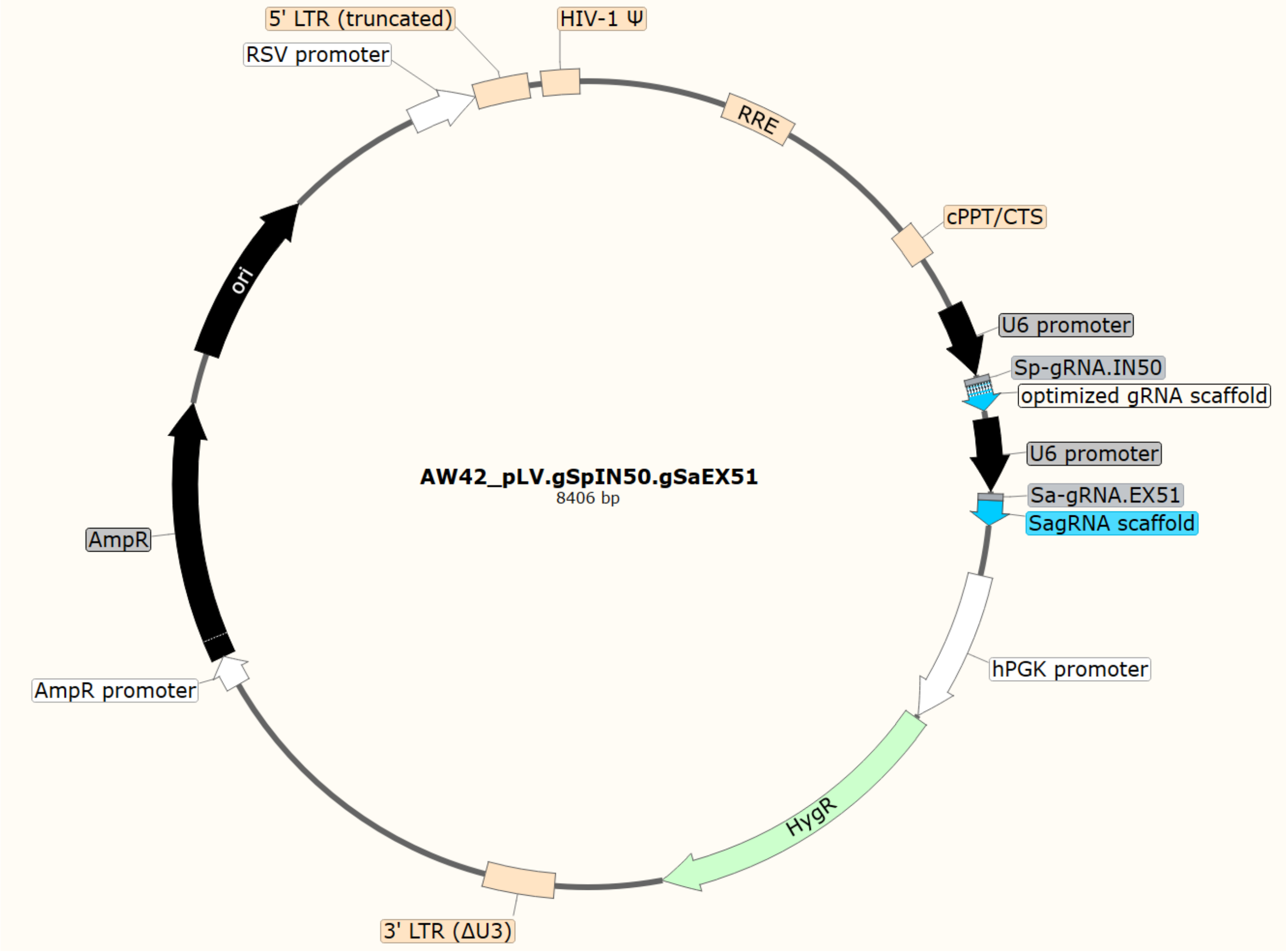

CGATGGGAAAAAATTCGGTTAAGGCCAGGGGGAAAGAAAAAATATAAATTAAAACATATAGTATGGGCAAGCAGG
GAGCTAGAACGATTCGCAGTTAATCCTGGCCTGTTAGAAACATCAGAAGGCTGTAGACAAATACTGGGACAGCTA
CAACCATCCCTTCAGACAGGATCAGAAGAACTTAGATCATTATATAATACAGTAGCAACCCTCTATTGTGTGCAT
CAAAGGATAGAGATAAAAGACACCAAGGAAGCTTTAGACAAGATAGAGGAAGAGCAAAACAAAAGTAAGACCACC
GCACAGCAAGCGGCCGCTGATCTTCAGACCTGGAGGAGGAGATATGAGGGACAATTGGAGAAGTGAATTATATAA
ATATAAAGTAGTAAAAATTGAACCATTAGGAGTAGCACCCACCAAGGCAAAGAGAAGAGTGGTGCAGAGAGAAAA
AAGAGCAGTGGGAATAGGAGCTTTGTTCCTTGGGTTCTTGGGAGCAGCAGGAAGCACTATGGGCGCAGCCTCAAT
GACGCTGACGGTACAGGCCAGACAATTATTGTCTGGTATAGTGCAGCAGCAGAACAATTTGCTGAGGGCTATTGA
GGCGCAACAGCATCTGTTGCAACTCACAGTCTGGGGCATCAAGCAGCTCCAGGCAAGAATCCTGGCTGTGGAAAG
ATACCTAAAGGATCAACAGCTCCTGGGGATTTGGGGTTGCTCTGGAAAACTCATTTGCACCACTGCTGTGCCTTG
GAATGCTAGTTGGAGTAATAAATCTCTGGAACAGATTGGAATCACACGACCTGGATGGAGTGGGACAGAGAAATT
AACAATTACACAAGCTTAATACACTCCTTAATTGAAGAATCGCAAAACCAGCAAGAAAAGAATGAACAAGAATTA
TTGGAATTAGATAAATGGGCAAGTTTGTGGAATTGGTTTAACATAACAAATTGGCTGTGGTATATAAAATTATTC
ATAATGATAGTAGGAGGCTTGGTAGGTTTAAGAATAGTTTTTGCTGTACTTTCTATAGTGAATAGAGTTAGGCAG
GGATATTCACCATTATCGTTTCAGACCCACCTCCCAACCCCGAGGGGACCCGACAGGCCCGAAGGAATAGAAGAA
GAAGGTGGAGAGAGAGACAGAGACAGATCCATTCGATTAGTGAACGGATCTCGACGGTATCGGTTAACTTTTAAA
AGAAAAGGGGGGATTGGGGGGTACAGTGCAGGGGAAAGAATAGTAGACATAATAGCAACAGACATACAAACTAAA
GAATTACAAAAACAAATTACAAAAATTCAAAATTTTATCGATCACGAGACTAGCCTCGACGATGGTCGAGTACCG
GGTAGGGGAGGCGCTTTTCCCAAGGCAGTCTGGAGCATGCGCTTTAGCAGCCCCGCTGGGCACTTGGCGCTACAC
AAGTGGCCTCTGGCCTCGCACACATTCCACATCCACCGGTACCTCTAGAGTCCGGCCGCCCCCTTCACCGAGGGC
CTATTTCCCATGATTCCTTCATATTTGCATATACGATACAAGGCTGTTAGAGAGATAATTGGAATTAATTTGACT
GTAAACACAAAGATATTAGTACAAAATACGTGACGTAGAAAGTAATAATTTCTTGGGTAGTTTGCAGTTTTAAAA
TTATGTTTTAAAATGGACTATCATATGCTTACCGTAACTTGAAAGTATTTCGATTTCTTGGCTTTATATATCTTG
TGGAAAGGACGAAACACC**GATACTTTGTTTAGCAATACA**GTTTCAGAGCTATGCTGGAAACAGCATAGCAAGTTG
AAATAAGGCTAGTCCGTTATCAACTTGAAAAAGTGGCACCGAGTCGGTGCTTTTTTTGAATTCGGTACCAAGGTC

GGGCAGGAAGAGGGCCTATTTCCCATGATTCCTTCATATTTGCATATACGATACAAGGCTGTTAGAGAGATAATT
AGAATTAATTTGACTGTAAACACAAAGATATTAGTACAAAATACGTGACGTAGAAAGTAATAATTTCTTGGGTAG
TTTGCAGTTTTAAAATTATGTTTTAAAATGGACTATCATATGCTTACCGTAACTTGAAAGTATTTCGATTTCTTG
GCTTTATATATCTTGTGGAAAGGACGAAACACC**GTTGTGTCACCAGAGTAACAGT**GTTTTAGTACTCTGTAATGA
AAATTACAGAATCTACTAAAACAAGGCAAAATGCCGTGTTTATCTCGTCAACTTGTTGGCGAGATTTTTTTAAGC
TTGGGCCGCTCGAGGTACCGGCGCGCCCGTACGACTAGAACTATAGCTAGCATGCGCAAATTTAAAGCGCTGATA
TCGATCGCGCGCAGATCTGTCATGATGATCATTGCAATTGGATCCATATATAGGGCCCGGGTTATAATTACCTCA
GGTCGAGGGGGTTGGGGTTGCGCCTTTTCCAAGGCAGCCCTGGGTTTGCGCAGGGACGCGGCTGCTCTGGGCGTG
GTTCCGGGAAACGCAGCGGCGCCGACCCTGGGTCTCGCACATTCTTCACGTCCGTTCGCAGCGTCACCCGGATCT
TCGCCGCTACCCTTGTGGGCCCCCCGGCGACGCTTCCTGCTCCGCCCCTAAGTCGGGAAGGTTCCTTGCGGTTCG
CGGCGTGCCGGACGTGACAAACGGAAGCCGCACGTCTCACTAGTACCCTCGCAGACGGACAGCGCCAGGGAGCAA
TGGCAGCGCGCCGACCGCGATGGGCTGTGGCCAATAGCGGCTGCTCAGCAGGGCGCGCCGAGAGCAGCGGCCGGG
AAGGGGCGGTGCGGGAGGCGGGGTGTGGGGCGGTAGTGTGGGCCCTGTTCCTGCCCGCGCGGTGTTCCGCATTCT
GCAAGCCTCCGGAGCGCACGTCGGCAGTCGGCTCCCTCGTTGACCGAATCACCGACCTCTCTCCCCAGGGGGATC
GTGATGAAAAAGCCTGAACTCACCGCGACGTCTGTCGAGAAGTTTCTGATCGAAAAGTTCGACAGCGTCTCCGAC
CTGATGCAGCTCTCGGAGGGCGAAGAATCTCGTGCTTTCAGCTTCGATGTAGGAGGGCGTGGATATGTCCTGCGG
GTAAATAGCTGCGCCGATGGTTTCTACAAAGATCGTTATGTTTATCGGCACTTTGCATCGGCCGCGCTCCCGATT
CCGGAAGTGCTTGACATTGGGGAATTCAGCGAGAGCCTGACCTATTGCATCTCCCGCCGTGCACAGGGTGTCACG
TTGCAAGACCTGCCTGAAACCGAACTGCCCGCTGTTCTGCAGCCGGTCGCGGAGGCCATGGATGCGATCGCTGCG
GCCGATCTTAGCCAGACGAGCGGGTTCGGCCCATTCGGACCGCAAGGAATCGGTCAATACACTACATGGCGTGAT
TTCATATGCGCGATTGCTGATCCCCATGTGTATCACTGGCAAACTGTGATGGACGACACCGTCAGTGCGTCCGTC
GCGCAGGCTCTCGATGAGCTGATGCTTTGGGCCGAGGACTGCCCCGAAGTCCGGCACCTCGTGCACGCGGATTTC
GGCTCCAACAATGTCCTGACGGACAATGGCCGCATAACAGCGGTCATTGACTGGAGCGAGGCGATGTTCGGGGAT
TCCCAATACGAGGTCGCCAACATCTTCTTCTGGAGGCCGTGGTTGGCTTGTATGGAGCAGCAGACGCGCTACTTC
GAGCGGAGGCATCCGGAGCTTGCAGGATCGCCGCGGCTCCGGGCGTATATGCTCCGCATTGGTCTTGACCAACTC
TATCAGAGCTTGGTTGACGGCAATTTCGATGATGCAGCTTGGGCGCAGGGTCGATGCGACGCAATCGTCCGATCC
GGAGCCGGGACTGTCGGGCGTACACAAATCGCCCGCAGAAGCGCGGCCGTCTGGACCGATGGCTGTGTAGAAGTA
CTCGCCGATAGTGGAAACCGACGCCCCAGCACTCGTCCGAGGGCAAAGGAATAGAGTAGATGCCGACCGAACAAG
AGCTGATTTCGAGAACGCCTCAGCCAGCAACTCGCGCGAGCCTAGCAAGGCAAATGCGAGAGAACGGCCTTACGC
TTGGTGGCACAGTTCTCGTCCACAGTTCGCTAAGCTCGCTCGGCTGGGTCGCGGGAGGGCCGGTCGCAGTGATTC
AGGCCCTTCTGGATTGTGTTGGTCCCCAGGGCACGATTGTCATGCCCACGCACTCGGGTGATCTGACTGATCCCG
CAGATTGGAGATCGCCGCCCGTGCCTGCCGATTGGGTGCAGATCGTACCTTTAAGACCAATGACTTACAAGGCAG
CTGTAGATCTTAGCCACTTTTTAAAAGAAAAGGGGGGACTGGAAGGGCTAATTCACTCCCAACGAAGACAAGATC
TGCTTTTTGCTTGTACTGGGTCTCTCTGGTTAGACCAGATCTGAGCCTGGGAGCTCTCTGGCTAACTAGGGAACC
CACTGCTTAAGCCTCAATAAAGCTTGCCTTGAGTGCTTCAAGTAGTGTGTGCCCGTCTGTTGTGTGACTCTGGTA
ACTAGAGATCCCTCAGACCCTTTTAGTCAGTGTGGAAAATCTCTAGCAGTAGTAGTTCATGTCATCTTATTATTC
AGTATTTATAACTTGCAAAGAAATGAATATCAGAGAGTGAGAGGAACTTGTTTATTGCAGCTTATAATGGTTACA
AATAAAGCAATAGCATCACAAATTTCACAAATAAAGCATTTTTTTCACTGCATTCTAGTTGTGGTTTGTCCAAAC
TCATCAATGTATCTTATCATGTCTGGCTCTAGCTATCCCGCCCCTAACTCCGCCCATCCCGCCCCTAACTCCGCC
CAGTTCCGCCCATTCTCCGCCCCATGGCTGACTAATTTTTTTTATTTATGCAGAGGCCGAGGCCGCCTCGGCCTC
TGAGCTATTCCAGAAGTAGTGAGGAGGCTTTTTTGGAGGCCTAGGGACGTACCCAATTCGCCCTATAGTGAGTCG
TATTACGCGCGCTCACTGGCCGTCGTTTTACAACGTCGTGACTGGGAAAACCCTGGCGTTACCCAACTTAATCGC
CTTGCAGCACATCCCCCTTTCGCCAGCTGGCGTAATAGCGAAGAGGCCCGCACCGATCGCCCTTCCCAACAGTTG
CGCAGCCTGAATGGCGAATGGCGCGACGCGCCCTGTAGCGGCGCATTAAGCGCGGCGGGTGTGGTGGTTACGCGC
AGCGTGACCGCTACACTTGCCAGCGCCCTAGCGCCCGCTCCTTTCGCTTTCTTCCCTTCCTTTCTCGCCACGTTC
GCCGGCTTTCCCCGTCAAGCTCTAAATCGGGGGCTCCCTTTAGGGTTCCGATTTAGTGCTTTACGGCACCTCGAC
CCCAAAAAACTTGATTAGGGTGATGGTTCACGTAGTGGGCCATCGCCCTGATAGACGGTTTTTCGCCCTTTGACG
TTGGAGTCCACGTTCTTTAATAGTGGACTCTTGTTCCAAACTGGAACAACACTCAACCCTATCTCGGTCTATTCT
TTTGATTTATAAGGGATTTTGCCGATTTCGGCCTATTGGTTAAAAAATGAGCTGATTTAACAAAAATTTAACGCG
AATTTTAACAAAATATTAACGTTTACAATTTCCCAGGTGGCACTTTTCGGGGAAATGTGCGCGGAACCCCTATTT
GTTTATTTTTCTAAATACATTCAAATATGTATCCGCTCATGAGACAATAACCCTGATAAATGCTTCAATAATATT
GAAAAAGGAAGAGTATGAGTATTCAACATTTCCGTGTCGCCCTTATTCCCTTTTTTGCGGCATTTTGCCTTCCTG
TTTTTGCTCACCCAGAAACGCTGGTGAAAGTAAAAGATGCTGAAGATCAGTTGGGTGCACGAGTGGGTTACATCG
AACTGGATCTCAACAGCGGTAAGATCCTTGAGAGTTTTCGCCCCGAAGAACGTTTTCCAATGATGAGCACTTTTA
AAGTTCTGCTATGTGGCGCGGTATTATCCCGTATTGACGCCGGGCAAGAGCAACTCGGTCGCCGCATACACTATT
CTCAGAATGACTTGGTTGAGTACTCACCAGTCACAGAAAAGCATCTTACGGATGGCATGACAGTAAGAGAATTAT
GCAGTGCTGCCATAACCATGAGTGATAACACTGCGGCCAACTTACTTCTGACAACGATCGGAGGACCGAAGGAGC
TAACCGCTTTTTTGCACAACATGGGGGATCATGTAACTCGCCTTGATCGTTGGGAACCGGAGCTGAATGAAGCCA
TACCAAACGACGAGCGTGACACCACGATGCCTGTAGCAATGGCAACAACGTTGCGCAAACTATTAACTGGCGAAC
TACTTACTCTAGCTTCCCGGCAACAATTAATAGACTGGATGGAGGCGGATAAAGTTGCAGGACCACTTCTGCGCT
CGGCCCTTCCGGCTGGCTGGTTTATTGCTGATAAATCTGGAGCCGGTGAGCGTGGGTCTCGCGGTATCATTGCAG

CACTGGGGCCAGATGGTAAGCCCTCCCGTATCGTAGTTATCTACACGACGGGGAGTCAGGCAACTATGGATGAAC
GAAATAGACAGATCGCTGAGATAGGTGCCTCACTGATTAAGCATTGGTAACTGTCAGACCAAGTTTACTCATATA
TACTTTAGATTGATTTAAAACTTCATTTTTAATTTAAAAGGATCTAGGTGAAGATCCTTTTTGATAATCTCATGA
CCAAAATCCCTTAACGTGAGTTTTCGTTCCACTGAGCGTCAGACCCCGTAGAAAAGATCAAAGGATCTTCTTGAG
ATCCTTTTTTTCTGCGCGTAATCTGCTGCTTGCAAACAAAAAAACCACCGCTACCAGCGGTGGTTTGTTTGCCGG
ATCAAGAGCTACCAACTCTTTTTCCGAAGGTAACTGGCTTCAGCAGAGCGCAGATACCAAATACTGTCCTTCTAG
TGTAGCCGTAGTTAGGCCACCACTTCAAGAACTCTGTAGCACCGCCTACATACCTCGCTCTGCTAATCCTGTTAC
CAGTGGCTGCTGCCAGTGGCGATAAGTCGTGTCTTACCGGGTTGGACTCAAGACGATAGTTACCGGATAAGGCGC
AGCGGTCGGGCTGAACGGGGGGTTCGTGCACACAGCCCAGCTTGGAGCGAACGACCTACACCGAACTGAGATACC
TACAGCGTGAGCTATGAGAAAGCGCCACGCTTCCCGAAGGGAGAAAGGCGGACAGGTATCCGGTAAGCGGCAGGG
TCGGAACAGGAGAGCGCACGAGGGAGCTTCCAGGGGGAAACGCCTGGTATCTTTATAGTCCTGTCGGGTTTCGCC
ACCTCTGACTTGAGCGTCGATTTTTGTGATGCTCGTCAGGGGGGCGGAGCCTATGGAAAAACGCCAGCAACGCGG
CCTTTTTACGGTTCCTGGCCTTTTGCTGGCCTTTTGCTCACATGTTCTTTCCTGCGTTATCCCCTGATTCTGTGG
ATAACCGTATTACCGCCTTTGAGTGAGCTGATACCGCTCGCCGCAGCCGAACGACCGAGCGCAGCGAGTCAGTGA
GCGAGGAAGCGGAAGAGCGCCCAATACGCAAACCGCCTCTCCCCGCGCGTTGGCCGATTCATTAATGCAGCTGGC
ACGACAGGTTTCCCGACTGGAAAGCGGGCAGTGAGCGCAACGCAATTAATGTGAGTTAGCTCACTCATTAGGCAC
CCCAGGCTTTACACTTTATGCTTCCGGCTCGTATGTTGTGTGGAATTGTGAGCGGATAACAATTTCACACAGGAA
ACAGCTATGACCATGATTACGCCAAGCGCGCAATTAACCCTCACTAAAGGGAACAAAAGCTGGAGCTGCAAGCTT
AATGTAGTCTTATGCAATACTCTTGTAGTCTTGCAACATGGTAACGATGAGTTAGCAACATGCCTTACAAGGAGA
GAAAAAGCACCGTGCATGCCGATTGGTGGAAGTAAGGTGGTACGATCGTGCCTTATTAGGAAGGCAACAGACGGG
TCTGACATGGATTGGACGAACCACTGAATTGCCGCATTGCAGAGATATTGTATTTAAGTGCCTAGCTCGATACAT
AAACGGGTCTCTCTGGTTAGACCAGATCTGAGCCTGGGAGCTCTCTGGCTAACTAGGGAACCCACTGCTTAAGCC
TCAATAAAGCTTGCCTTGAGTGCTTCAAGTAGTGTGTGCCCGTCTGTTGTGTGACTCTGGTAACTAGAGATCCCT
CAGACCCTTTTAGTCAGTGTGGAAAATCTCTAGCAGTGGCGCCCGAACAGGGACCTGAAAGCGAAAGGGAAACCA
GAGCTCTCTCGACGCAGGACTCGGCTTGCTGAAGCGCGCACGGCAAGAGGCGAGGGGCGGCGACTGGTGAGTACG
CCAAAAATTTTGACTAGCGGAGGCTAGAAGGAGAGAGATGGGTGCGAGAGCGTCAGTATTAAGCGGGGGAGAATT
AGATCG

Figure S7. Map and sequence of lentiviral vector transfer plasmid AW42_pLV.gSp^{IN50}.gSa^{EX51}. The HIV-1 *cis*-acting elements required for vector genome amplification and packaging are presented in light orange. 5'LTR (truncated), truncated 5' long terminal repeat; HIV-1 Ψ, packaging signal; RRE, Rev response element; cPPT/CTS, central polypurine tract and central termination sequence; 3' LTR (ΔU3), 3' self-inactivating long terminal repeat; hPGK promoter, human *phosphoglycerate kinase 1* regulatory sequences; RSV, Rous sarcoma virus enhancer/promoter; U6 promoter, RNA polymerase III promoter for human snRNA; Sp-gRNA.IN50 and Sa-gRNA.EX51, *Staphylococcus aureus* and *Streptococcus pyogenes* gRNAs with spacers (underlined) targeting *DMD* intron 50 and exon 51 sequences, respectively. HygR, gene coding for *E. coli* aminoglycoside phosphotransferase conferring resistance to hygromycin.

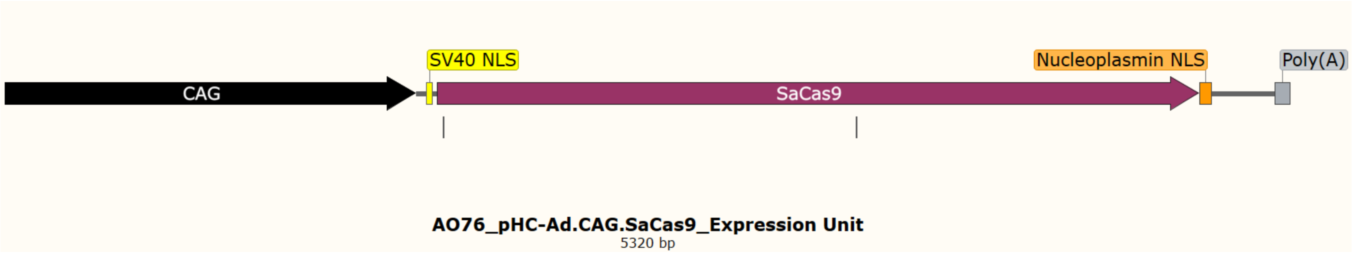

CTAGTTATTAATAGTAATCAATTACGGGGTCATTAGTTCATAGCCCATATATGGAGTTCCGCGTTACATAACTTA
CGGTAAATGGCCCGCCTGGCTGACCGCCCAACGACCCCCGCCCATTGACGTCAATAATGACGTATGTTCCCATAG
TAACGCCAATAGGGACTTTCCATTGACGTCAATGGGTGGAGTATTTACGGTAAACTGCCCACTTGGCAGTACATC
AAGTGTATCATATGCCAAGTACGCCCCCTATTGACGTCAATGACGGTAAATGGCCCGCCTGGCATTATGCCCAGT
ACATGACCTTATGGGACTTTCCTACTTGGCAGTACATCTACGTATTAGTCATCGCTATTACCATGGTCGAGGTG
AGCCCCACGTTCTGCTTCACTCTCCCCATCTCCCCCCCCTCCCCACCCCCAATTTTGTATTTATTTATTTTTTAA
TTATTTTGTGCAGCGATGGGGGCGGGGGGGGGGGGGGCGCGCGCCAGGCGGGGCGGGGCGGGGCGAGGGGCGGGG
CGGGGCGAGGCGGAGAGGTGCGGCGGCAGCCAATCAGAGCGGCGCGCTCCGAAAGTTTCCTTTTATGGCGAGGCG
GCGGCGGCGGCGGCCCTATAAAAAGCGAAGCGCGCGGCGGGCGGGAGTCGCTGCGTTGCCTTCGCCCCGTGCCCC
GCTCCGCGCCGCCTCGCGCCGCCCGCCCCGGCTCTGACTGACCGCGTTACTCCCACAGGTGAGCGGGCGGGACGG
CCCTTCTCCTCCGGGCTGTAATTAGCGCTTGGTTTAATGACGGCTCGTTTCTTTTCTGTGGCTGCGTGAAAGCCT
TAAAGGGCTCCGGGAGGGCCCTTTGTGCGGGGGGGAGCGGCTCGGGGGGTGCGTGCGTGTGTGTGTGCGTGGGGA
GCGCCGCGTGCGGCCCGCGCTGCCCGGCGGCTGTGAGCGCTGCGGGCGCGGCGCGGGGCTTTGTGCGCTCCGCGT
GTGCGCGAGGGGAGCGCGGCCGGGGGCGGTGCCCCGCGGTGCGGGGGGGCTGCGAGGGGAACAAAGGCTGCGTGC
GGGGTGTGTGCGTGGGGGGGTGAGCAGGGGGTGTGGGCGCGGCGGTCGGGCTGTAACCCCCCCCTGCACCCCCCT
CCCCGAGTTGCTGAGCACGGCCCGGCTTCGGGTGCGGGGCTCCGTGCGGGGCGTGGCGCGGGGCTCGCCGTGCCG
GGCGGGGGGTGGCGGCAGGTGGGGGTGCCGGGCGGGGCGGGGCCGCCTCGGGCCGGGGAGGGCTCGGGGGAGGGG
CGCGGCGGCCCCGGAGCGCCGGCGGCTGTCGAGGCGCGGCGAGCCGCAGCCATTGCCTTTTATGGTAATCGTGCG
AGAGGGCGCAGGGACTTCCTTTGTCCCAAATCTGGCGGAGCCGAAATCTGGGAGGCGCCGCCGCACCCCCTCTAG
CGGGCGCGGGCGAAGCGGTGCGGCGCCGGCAGGAAGGAAATGGGCGGGGAGGGCCTTCGTGCGTCGCCGCGCCGC
CGTCCCCTTCTCCATCTCCAGCCTCGGGGCTGCCGCAGGGGGACGGCTGCCTTCGGGGGGGACGGGGCAGGGCGG
GGTTCGGCTTCTGGCGTGTGACCGGCGGCTCTAGAGCCTCTGCTAACCATGTTCATGCCTTCTTCTTTTTCCTAC
AGCTCCTGGGCAACGTGCTGGTTGTTGTGCTGTCTCATCATTTTGGCAAAGAATTATCGCATGCCTGCAGAGCTC
TAGAGTCCCGGTGCCACC**ATG**GCCCCAAAGAAGAAGCGGAAGGTCGGTATCCACGGAGTCCCAGCAGCCAAGCGG
AACTACATCCTGGGCCTGGACATCGGCATCACCAGCGTGGGCTACGGCATCATCGACTACGAGACACGGGACGTG
ATCGATGCCGGCGTGCGGCTGTTCAAAGAGGCCAACGTGGAAAACAACGAGGGCAGGCGGAGCAAGAGAGGCGCC
AGAAGGCTGAAGCGGCGGAGGCGGCATAGAATCCAGAGAGTGAAGAAGCTGCTGTTCGACTACAACCTGCTGACC
GACCACAGCGAGCTGAGCGGCATCAACCCCTACGAGGCCAGAGTGAAGGGCCTGAGCCAGAAGCTGAGCGAGGAA
GAGTTCTCTGCCGCCCTGCTGCACCTGGCCAAGAGAAGAGGCGTGCACAACGTGAACGAGGTGGAAGAGGACACC
GGCAACGAGCTGTCCACCAAAGAGCAGATCAGCCGGAACAGCAAGGCCCTGGAAGAGAAATACGTGGCCGAACTG
CAGCTGGAACGGCTGAAGAAAGACGGCGAAGTGCGGGGCAGCATCAACAGATTCAAGACCAGCGACTACGTGAAA
GAAGCCAAACAGCTGCTGAAGGTGCAGAAGGCCTACCACCAGCTGGACCAGAGCTTCATCGACACCTACATCGAC
CTGCTGGAAACCCGGCGGACCTACTATGAGGGACCTGGCGAGGGCAGCCCCTTCGGCTGGAAGGACATCAAAGAA
TGGTACGAGATGCTGATGGGCCACTGCACCTACTTCCCCGAGGAACTGCGGAGCGTGAAGTACGCCTACAACGCC
GACCTGTACAACGCCCTGAACGACCTGAACAATCTCGTGATCACCAGGGACGAGAACGAGAAGCTGGAATATTAC
GAGAAGTTCCAGATCATCGAGAACGTGTTCAAGCAGAAGAAGAAGCCCACCCTGAAGCAGATCGCCAAAGAAATC
CTCGTGAACGAAGAGGATATTAAGGGCTACAGAGTGACCAGCACCGGCAAGCCCGAGTTCACCAACCTGAAGGTG
TACCACGACATCAAGGACATTACCGCCCGGAAAGAGATTATTGAGAACGCCGAGCTGCTGGATCAGATTGCCAAG
ATCCTGACCATCTACCAGAGCAGCGAGGACATCCAGGAAGAACTGACCAATCTGAACTCCGAGCTGACCCAGGAA
GAGATCGAGCAGATCTCTAATCTGAAGGGCTATACCGGCACCCACAACCTGAGCCTGAAGGCCATCAACCTGATC
CTGGACGAGCTGTGGCACACCAACGACAACCAGATCGCTATCTTCAACCGGCTGAAGCTGGTGCCCAAGAAGGTG
GACCTGTCCCAGCAGAAAGAGATCCCCACCACCCTGGTGGACGACTTCATCCTGAGCCCCGTCGTGAAGAGAAGC
TTCATCCAGAGCATCAAAGTGATCAACGCCATCATCAAGAAGTACGGCCTGCCCAACGACATCATTATCGAGCTG
GCCCGCGAGAAGAACTCCAAGGACGCCCAGAAAATGATCAACGAGATGCAGAAGCGGAACCGGCAGACCAACGAG
CGGATCGAGGAAATCATCCGGACCACCGGCAAAGAGAACGCCAAGTACCTGATCGAGAAGATCAAGCTGCACGAC

ATGCAGGAAGGCAAGTGCCTGTACAGCCTGGAAGCCATCCCTCTGGAAGATCTGCTGAACAACCCCTTCAACTAT
GAGGTGGACCACATCATCCCCAGAAGCGTGTCCTTCGACAACAGCTTCAACAACAAGGTGCTCGTGAAGCAGGAA
GAAAACAGCAAGAAGGGCAACCGGACCCCATTCCAGTACCTGAGCAGCAGCGACAGCAAGATCAGCTACGAAACC
TTCAAGAAGCACATCCTGAATCTGGCCAAGGGCAAGGGCAGAATCAGCAAGACCAAGAAAGAGTATCTGCTGGAA
GAACGGGACATCAACAGGTTCTCCGTGCAGAAAGACTTCATCAACCGGAACCTGGTGGATACCAGATACGCCACC
AGAGGCCTGATGAACCTGCTGCGGAGCTACTTCAGAGTGAACAACCTGGACGTGAAAGTGAAGTCCATCAATGGC
GGCTTCACCAGCTTTCTGCGGCGGAAGTGGAAGTTTAAGAAAGAGCGGAACAAGGGGTACAAGCACCACGCCGAG
GACGCCCTGATCATTGCCAACGCCGATTTCATCTTCAAAGAGTGGAAGAAACTGGACAAGGCCAAAAAAGTGATG
GAAAACCAGATGTTCGAGGAAAAGCAGGCCGAGAGCATGCCCGAGATCGAAACCGAGCAGGAGTACAAAGAGATC
TTCATCACCCCCCACCAGATCAAGCACATTAAGGACTTCAAGGACTACAAGTACAGCCACCGGGTGGACAAGAAG
CCTAATAGAGAGCTGATTAACGACACCCTGTACTCCACCCGGAAGGACGACAAGGGCAACACCCTGATCGTGAAC
AATCTGAACGGCCTGTACGACAAGGACAATGACAAGCTGAAAAAGCTGATCAACAAGAGCCCCGAAAAGCTGCTG
ATGTACCACCACGACCCCCAGACCTACCAGAAACTGAAGCTGATTATGGAACAGTACGGCGACGAGAAGAATCCC
CTGTACAAGTACTACGAGGAAACCGGGAACTACCTGACCAAGTACTCCAAAAAGGACAACGGCCCCGTGATCAAG
AAGATTAAGTATTACGGCAACAAACTGAACGCCCATCTGGACATCACCGACGACTACCCCAACAGCAGAAACAAG
GTCGTGAAGCTGTCCCTGAAGCCCTACAGATTCGACGTGTACCTGGACAATGGCGTGTACAAGTTCGTGACCGTG
AAGAATCTGGATGTGATCAAAAAAGAAAACTACTACGAAGTGAATAGCAAGTGCTATGAGGAAGCTAAGAAGCTG
AAGAAGATCAGCAACCAGGCCGAGTTTATCGCCTCCTTCTACAACAACGATCTGATCAAGATCAACGGCGAGCTG
TATAGAGTGATCGGCGTGAACAACGACCTGCTGAACCGGATCGAAGTGAACATGATCGACATCACCTACCGCGAG
TACCTGGAAAACATGAACGACAAGAGGCCCCCCAGGATCATTAAGACAATCGCCTCCAAGACCCAGAGCATTAAG
AAGTACAGCACAGACATTCTGGGCAACCTGTATGAAGTGAAATCTAAGAAGCACCCTCAGATCATCAAAAAGGGC
AAAAGGCCGGCGGCCACGAAAAAGGCCGGCCAGGCAAAAAAGAAAAAGGGATCCTACCCATACGATGTTCCAGAT
TACGCTTACCCATACGATGTTCCAGATTACGCTTACCCATACGATGTTCCAGATTACGCTTAAGAATTGGCCGCA
CTTAAGTTACGCGTGGAAATTCACTCCTCAGGTGCAGGCTGCCTATCAGAAGGTGGTGGCTGGTGTGGCCAATGC
CCTGGCTCACAAATACCACTGAGATCTTTTTCCCTCTGCCAAAAATTATGGGGACATCATGAAGCCCCTTGAGCA
TCTGACTTCTGGCTAATAAAGGAAATTTATTTTCATTGCAATAGTGTGTTGGAATTTTTTGTGTCTCTCA

Figure S8. Map and sequence of expression unit in AO76_pHC-Ad.CAG.SaCas9. CAG, hybrid promoter composed of the human cytomegalovirus *immediate-early* enhancer, the chicken *β-actin* promoter and a chimeric intron formed by chicken *β-actin* and rabbit *β-globin* sequences; SaCas9, Cas9 endonuclease derived from the *Staphylococcus aureus* Type II CRISPR-Cas system; SV40 NLS, nuclear localization signal motif from the simian virus 40 large T antigen; Nucleoplasmin NLS, nucleoplasmin nuclear localization signal; Poly(A), rabbit *β-globin* polyadenylation signal. The different elements are color coded in the expression unit map and respective nucleotide sequence.

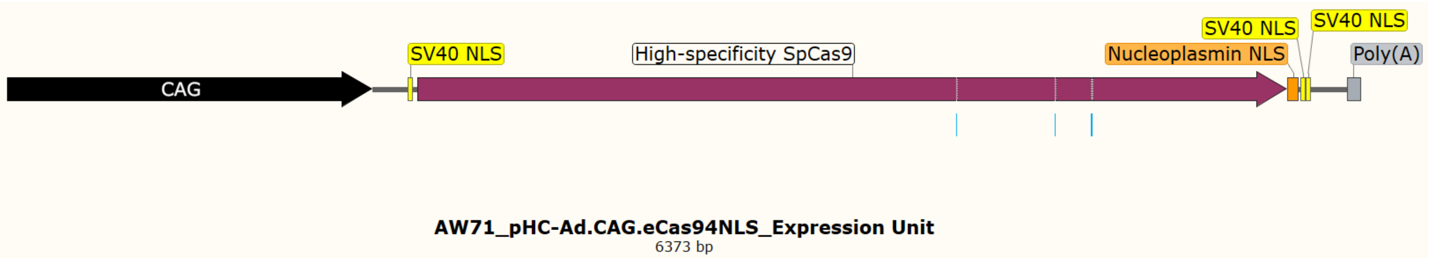

TCGACATTGATTATTGACTAGTTATTAATAGTAATCAATTACGGGGTCATTAGTTCATAGCCCATATA
TGGAGTTCCGCGTTACATAACTTACGGTAAATGGCCCGCCTGGCTGACCGCCCAACGACCCCCGCCCA
TTGACGTCAATAATGACGTATGTTCCCATAGTAACGCCAATAGGGACTTTCCATTGACGTCAATGGGTGGAGTAT
TTACGGTAAACTGCCCACTTGGCAGTACATCAAGTGTATCATATGCCAAGTACGCCCCCTATTGACGTCAATGAC
GGTAAATGGCCCGCCTGGCATTATGCCCAGTACATGACCTTATGGGACTTTCCTACTTGGCAGTACATCTACGTA
TTAGTCATCGCTATTACCATGGTCGAGGTGAGCCCCACGTTCTGCTTCACTCTCCCCATCTCCCCCCCCTCCCCA
CCCCCAATTTTGTATTTATTTATTTTTTAATTATTTTGTGCAGCGATGGGGGCGGGGGGGGGGGGGCGCGCGCC
AGGCGGGGCGGGGCGGGGCGAGGGGCGGGGCGGGGCGAGGCGGAGAGGTGCGGCGGCAGCCAATCAGAGCGGCGC

GCTCCGAAAGTTTCCTTTTATGGCGAGGCGGCGGCGGCGGCGGCCCTATAAAAAGCGAAGCGCGCGGCGGGCGGG
AGTCGCTGCGTTGCCTTCGCCCCGTGCCCCGCTCCGCGCCGCCTCGCGCCGCCCGCCCCGGCTCTGACTGACCGC
GTTACTCCCACAGGTGAGCGGGCGGGACGGCCCTTCTCCTCCGGGCTGTAATTAGCGCTTGGTTTAATGACGGCT
CGTTTCTTTTCTGTGGCTGCGTGAAAGCCTTAAAGGGCTCCGGGAGGGCCCTTTGTGCGGGGGGGAGCGGCTCGG
GGGGTGCGTGCGTGTGTGTGTGCGTGGGGAGCGCCGCGTGCGGCCCGCGCTGCCCGGCGGCTGTGAGCGCTGCGG
GCGCGGCGCGGGGCTTTGTGCGCTCCGCGTGTGCGCGAGGGGAGCGCGGCCGGGGGCGGTGCCCCGCGGTGCGGG
GGGGCTGCGAGGGGAACAAAGGCTGCGTGCGGGGTGTGTGCGTGGGGGGGTGAGCAGGGGGTGTGGGCGCGGCGG
TCGGGCTGTAACCCCCCCCTGCACCCCCCTCCCCGAGTTGCTGAGCACGGCCCGGCTTCGGGTGCGGGGCTCCGT
ACGGGGCGTGGCGCGGGGCTCGCCGTGCCGGGCGGGGGGTGGCGGCAGGTGGGGGTGCCGGGCGGGGCGGGGCCG
CCTCGGGCCGGGGAGGGCTCGGGGGAGGGGCGCGGCGGCCCCGGAGCGCCGGCGGCTGTCGAGGCGCGGCGAGCC
GCAGCCATTGCCTTTTATGGTAATCGTGCGAGAGGGCGCAGGGACTTCCTTTGTCCCAAATCTGTGCGGAGCCGA
AATCTGGGAGGCGCCGCCGCACCCCCTCTAGCGGGCGCGGGGCGAAGCGGTGCGGCGCCGGCAGGAAGGAAATGG
GCGGGGAGGGCCTTCGTGCGTCGCCGCGCCGCCGTCCCCTTCTCCCTCTCCAGCCTCGGGGCTGTCCGCGGGGGG
ACGGCTGCCTTCGGGGGGGACGGGGCAGGGCGGGGTTCGGCTTCTGGCGTGTGACCGGCGGCTCTAGAGCCTCTG
CTAACCATGTTCATGCCTTCTTCTTTTTCCTACAGCTCCTGGGCAACGTGCTGGTTATTGTGCTGTCTCATCATT
TTGGCAAAGAATTATCGCATGCCTGCAGAGCTCTAGAGTCTAATGTTTAATTACCTGGAGCACCTGCCTGAAATC
ACTTTTTTTCAGGTTGGACCGGTGCCACC**ATG**GACTATAAGGACCACGACGGAGACTACAAGGATCATGATATTG
ATTACAAAGACGATGACGATAAGATGGCCCCAAAGAAGAAGCGGAAGGTCGGTATCCACGGAGTCCCAGCAGCCG
ACAAGAAGTACAGCATCGGCCTGGACATCGGCACCAACTCTGTGGGCTGGGCCGTGATCACCGACGAGTACAAGG
TGCCCAGCAAGAAATTCAAGGTGCTGGGCAACACCGACCGGCACAGCATCAAGAAGAACCTGATCGGAGCCCTGC
TGTTCGACAGCGGCGAAACAGCCGAGGCCACCCGGCTGAAGAGAACCGCCAGAAGAAGATACACCAGACGGAAGA
ACCGGATCTGCTATCTGCAAGAGATCTTCAGCAACGAGATGGCCAAGGTGGACGACAGCTTCTTCCACAGACTGG
AAGAGTCCTTCCTGGTGGAAGAGGATAAGAAGCACGAGCGGCACCCCATCTTCGGCAACATCGTGGACGAGGTGG
CCTACCACGAGAAGTACCCCACCATCTACCACCTGAGAAAGAAACTGGTGGACAGCACCGACAAGGCCGACCTGC
GGCTGATCTATCTGGCCCTGGCCCACATGATCAAGTTCCGGGGCCACTTCCTGATCGAGGGCGACCTGAACCCCG
ACAACAGCGACGTGGACAAGCTGTTCATCCAGCTGGTGCAGACCTACAACCAGCTGTTCGAGGAAAACCCCATCA
ACGCCAGCGGCGTGGACGCCAAGGCCATCCTGTCTGCCAGACTGAGCAAGAGCAGACGGCTGGAAAATCTGATCG
CCCAGCTGCCCGGCGAGAAGAAGAATGGCCTGTTCGGAAACCTGATTGCCCTGAGCCTGGGCCTGACCCCCAACT
TCAAGAGCAACTTCGACCTGGCCGAGGATGCCAAACTGCAGCTGAGCAAGGACACCTACGACGACGACCTGGACA
ACCTGCTGGCCCAGATCGGCGACCAGTACGCCGACCTGTTTCTGGCCGCCAAGAACCTGTCCGACGCCATCCTGC
TGAGCGACATCCTGAGAGTGAACACCGAGATCACCAAGGCCCCCCTGAGCGCCTCTATGATCAAGAGATACGACG
AGCACCACCAGGACCTGACCCTGCTGAAAGCTCTCGTGCGGCAGCAGCTGCCTGAGAAGTACAAAGAGATTTTCT
TCGACCAGAGCAAGAACGGCTACGCCGGCTACATTGACGGCGGAGCCAGCCAGGAAGAGTTCTACAAGTTCATCA
AGCCCATCCTGGAAAAGATGGACGGCACCGAGGAACTGCTCGTGAAGCTGAACAGAGAGGACCTGCTGCGGAAGC
AGCGGACCTTCGACAACGGCAGCATCCCCCACCAGATCCACCTGGGAGAGCTGCACGCCATTCTGCGGCGGCAGG
AAGATTTTTACCCATTCCTGAAGGACAACCGGGAAAAGATCGAGAAGATCCTGACCTTCCGCATCCCCTACTACG
TGGGCCCTCTGGCCAGGGGAAACAGCAGATTCGCCTGGATGACCAGAAAGAGCGAGGAAACCATCACCCCCTGGA
ACTTCGAGGAAGTGGTGGACAAGGGCGCTTCCGCCCAGAGCTTCATCGAGCGGATGACCAACTTCGATAAGAACC
TGCCCAACGAGAAGGTGCTGCCCAAGCACAGCCTGCTGTACGAGTACTTCACCGTGTATAACGAGCTGACCAAAG
TGAAATACGTGACCGAGGGAATGAGAAAGCCCGCCTTCCTGAGCGGCGAGCAGAAAAAGGCCATCGTGGACCTGC
TGTTCAAGACCAACCGGAAAGTGACCGTGAAGCAGCTGAAAGAGGACTACTTCAAGAAAATCGAGTGCTTCGACT
CCGTGGAAATCTCCGGCGTGGAAGATCGGTTCAACGCCTCCCTGGGCACATACCACGATCTGCTGAAAATTATCA
AGGACAAGGACTTCCTGGACAATGAGGAAAACGAGGACATTCTGGAAGATATCGTGCTGACCCTGACACTGTTTG
AGGACAGAGAGATGATCGAGGAACGGCTGAAAACCTATGCCCACCTGTTCGACGACAAAGTGATGAAGCAGCTGA
AGCGGCGGAGATACACCGGCTGGGGCAGGCTGAGCCGGAAGCTGATCAACGGCATCCGGGACAAGCAGTCCGGCA
AGACAATCCTGGATTTCCTGAAGTCCGACGGCTTCGCCAACAGAAACTTCATGCAGCTGATCCACGACGACAGCC
TGACCTTTAAAGAGGACATCCAGAAAGCCCAGGTGTCCGGCCAGGGCGATAGCCTGCACGAGCACATTGCCAATC
TGGCCGGCAGCCCCGCCATTAAGAAGGGCATCCTGCAGACAGTGAAGGTGGTGGACGAGCTCGTGAAAGTGATGG
GCCGGCACAAGCCCGAGAACATCGTGATCGAAATGGCCAGAGAGAACCAGACCACCCAGAAGGGACAGAAGAACA
GCCGCGAGAGAATGAAGCGGATCGAAGAGGGCATCAAAGAGCTGGGCAGCCAGATCCTGAAAGAACACCCCGTGG
AAAACACCCAGCTGCAGAACGAGAAGCTGTACCTGTACTACCTGCAGAATGGGCGGGATATGTACGTGGACCAGG
AACTGGACATCAACCGGCTGTCCGACTACGATGTGGACCATATCGTGCCTCAGAGCTTTCTGGCCGACGACTCCA
TCGACAACAAGGTGCTGACCAGAAGCGACAAGAACCGGGGCAAGAGCGACAACGTGCCCTCCGAAGAGGTCGTGA
AGAAGATGAAGAACTACTGGCGGCAGCTGCTGAACGCCAAGCTGATTACCCAGAGAAAGTTCGACAATCTGACCA
AGGCCGAGAGAGGCGGCCTGAGCGAACTGGATAAGGCCGGCTTCATCAAGAGACAGCTGGTGGAAACCCGGCAGA
TCACAAAGCACGTGGCACAGATCCTGGACTCCCGGATGAACACTAAGTACGACGAGAATGACAAGCTGATCCGGG
AAGTGAAAGTGATCACCCTGAAGTCCAAGCTGGTGTCCGATTTCCGGAAGGATTTCCAGTTTTACAAAGTGCGCG

AGATCAACAACTACCACCACGCCCACGACGCCTACCTGAACGCCGTCGTGGGAACCGCCCTGATCAAAAAGTACC
CTGCGCTGGAAAGCGAGTTCGTGTACGGCGACTACAAGGTGTACGACGTGCGGAAGATGATCGCCAAGAGCGAGC
AGGAAATCGGCAAGGCTACCGCCAAGTACTTCTTCTACAGCAACATCATGAACTTTTTCAAGACCGAGATTACCC
TGGCCAACGGCGAGATCCGGAAGGCGCCTCTGATCGAGACAAACGGCGAAACCGGGGAGATCGTGTGGGATAAGG
GCCGGGATTTTGCCACCGTGCGGAAAGTGCTGAGCATGCCCCAAGTGAATATCGTGAAAAAGACCGAGGTGCAGA
CAGGCGGCTTCAGCAAAGAGTCTATCCTGCCCAAGAGGAACAGCGATAAGCTGATCGCCAGAAAGAAGGACTGGG
ACCCTAAGAAGTACGGCGGCTTCGACAGCCCCACCGTGGCCTATTCTGTGCTGGTGGTGGCCAAAGTGGAAAAGG
GCAAGTCCAAGAAACTGAAGAGTGTGAAAGAGCTGCTGGGGATCACCATCATGGAAAGAAGCAGCTTCGAGAAGA
ATCCCATCGACTTTCTGGAAGCCAAGGGCTACAAAGAAGTGAAAAAGGACCTGATCATCAAGCTGCCTAAGTACT
CCCTGTTCGAGCTGGAAAACGGCCGGAAGAGAATGCTGGCCTCTGCCGGCGAACTGCAGAAGGGAAACGAACTGG
CCCTGCCCTCCAAATATGTGAACTTCCTGTACCTGGCCAGCCACTATGAGAAGCTGAAGGGCTCCCCCGAGGATA
ATGAGCAGAAACAGCTGTTTGTGGAACAGCACAAGCACTACCTGGACGAGATCATCGAGCAGATCAGCGAGTTCT
CCAAGAGAGTGATCCTGGCCGACGCTAATCTGGACAAAGTGCTGTCCGCCTACAACAAGCACCGGGATAAGCCCA
TCAGAGAGCAGGCCGAGAATATCATCCACCTGTTTACCCTGACCAATCTGGGAGCCCCTGCCGCCTTCAAGTACT
TTGACACCACCATCGACCGGAAGAGGTACACCAGCACCAAAGAGGTGCTGGACGCCACCCTGATCCACCAGAGCA
TCACCGGCCTGTACGAGACACGGATCGACCTGTCTCAGCTGGGAGGCGAC**AAAAGGCCGGCGGCCACGAAAAAGG**
CCGGCCAGGCAAAAAAGAAAAAGGCTAGCGGCTCCCCCAAGAAAAAACGCAAGGTGGAAGATCCTAAGAAAAAGC
GGAAAGTGTAAGAATTCCCTGCAGGACGCGTGGAAATTCACTCCTCAGGTGCAGGCTGCCTATCAGAAGGTGGTG
GCTGGTGTGGCCAATGCCCTGGCTCACAAATACCACTGAGATCTTTTTCCCTCTGCCAAAAATTATGGGGACATC
ATGAAGCCCCTTGAGCATCTGACTTCTGGCTAATAAAGGAAATTTATTTTCATTGCAATAGTGTGTTGGAATTTT
TTGTGTCTCTCA

Figure S9. Map and sequence of expression unit in AW71_pHC-Ad.CAG.eCas9.4NLS. CAG, hybrid promoter consisting of the human cytomegalovirus *immediate-early* enhancer, the chicken *β-actin* promoter and a chimeric intron formed by chicken *β-actin* and rabbit *β-globin* sequences; High-specificity SpCas9, optimized variant of eSpCas9(1.1) derived from the *Streptococcus pyogenes* Type II CRISPR-Cas system. The point mutations K848A, K1003A and R1060A, conferring enhanced specificity, are marked (vertical cyan lines); SV40 NLS, nuclear localization signal motif from the simian virus 40 large T antigen; Nucleoplasmin NLS, nucleoplasmin nuclear localization signal; Poly(A), rabbit *β-globin* polyadenylation signal. The different features are color coded in the expression unit map and respective nucleotide sequence.

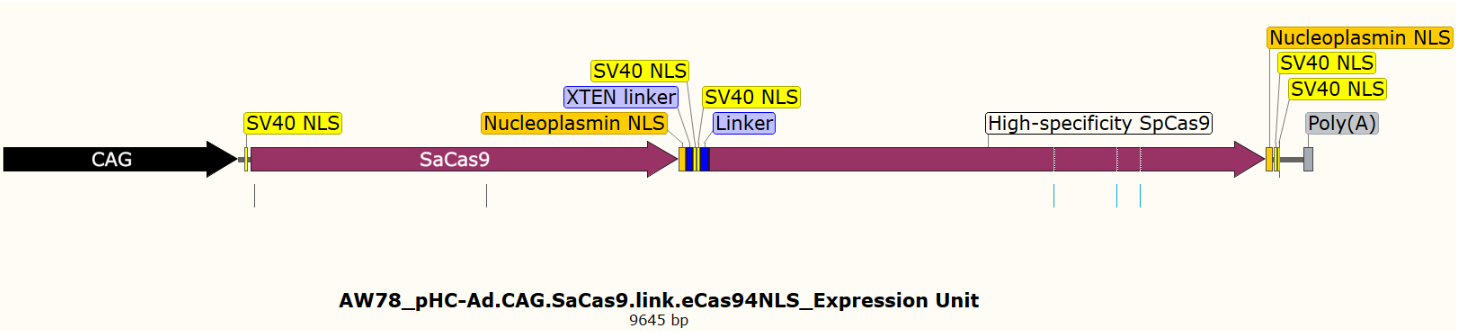

TCGACATTGATTATTGACTAGTTATTAATAGTAATCAATTACGGGGTCATTAGTTCATAGCCCATATATGGAGTT
CCGCGTTACATAACTTACGGTAAATGGCCCGCCTGGCTGACCGCCCAACGACCCCCGCCCATTGACGTCAATAAT
GACGTATGTTCCCATAGTAACGCCAATAGGGACTTTCCATTGACGTCAATGGGTGGAGTATTTACGGTAAACTGC
CCACTTGGCAGTACATCAAGTGTATCATATGCCAAGTACGCCCCCTATTGACGTCAATGACGGTAAATGGCCCGC
CTGGCATTATGCCCAGTACATGACCTTATGGGACTTTCCTACTTGGCAGTACATCTACGTATTAGTCATCGCTAT
TACCATGGTCGAGGTGAGCCCCACGTTCTGCTTCACTCTCCCCATCTCCCCCCCCTCCCCACCCCCAATTTTGTA
TTTATTTATTTTTTAATTATTTTGTGCAGCGATGGGGGCGGGGGGGGGGGGGGGGCGCGCGCCAGGCGGGGCGGG
GCGGGGCGAGGGGCGGGGCGGGGCGAGGCGGAGAGGTGCGGCGGCAGCCAATCAGAGCGGCGCGCTCCGAAAGTT
TCCTTTTATGGCGAGGCGGCGGCGGCGGCGGCCCTATAAAAAGCGAAGCGCGCGGCGGGCGGGAGTCGCTGCGAC

GCTGCCTTCGCCCCGTGCCCCGCTCCGCCGCCGCCTCGCGCCGCCCGCCCCGGCTCTGACTGACCGCGTTACTCC
CACAGGTGAGCGGGCGGGACGGCCCTTCTCCTCCGGGCTGTAATTAGCGCTTGGTTTAATGACGGCTTGTTTCTT
TTCTGTGGCTGCGTGAAAGCCTTGAGGGGCTCCGGGAGGGCCCTTTGTGCGGGGGGAGCGGCTCGGGGGGTGCGT
GCGTGTGTGTGTGCGTGGGGAGCGCCGCGTGCGGCTCCGCGCTGCCCGGCGGCTGTGAGCGCTGCGGGCGCGGCG
CGGGGCTTTGTGCGCTCCGCAGTGTGCGCGAGGGGAGCGCGGCCGGGGGCGGTGCCCCGCGGTGCGGGGGGGGCT
GCGAGGGGAACAAAGGCTGCGTGCGGGGTGTGTGCGTGGGGGGGTGAGCAGGGGGTGTGGGCGCGTCGGTCGGGC
TGCAACCCCCCCTGCACCCCCCTCCCCGAGTTGCTGAGCACGGCCCGGCTTCGGGTGCGGGGCTCCGTACGGGGC
GTGGCGCGGGGCTCGCCGTGCCGGGCGGGGGGTGGCGGCAGGTGGGGGTGCCGGGCGGGGCGGGGCCGCCTCGGG
CCGGGGAGGGCTCGGGGGAGGGGCGCGGCGGCCCCCGGAGCGCCGGCGGCTGTCGAGGCGCGGCGAGCCGCAGCC
ATTGCCTTTTATGGTAATCGTGCGAGAGGGCGCAGGGACTTCCTTTGTCCCAAATCTGTGCGGAGCCGAAATCTG
GGAGGCGCCGCCGCACCCCCTCTAGCGGGCGCGGGGCGAAGCGGTGCGGCGCCGGCAGGAAGGAAATGGGCGGGG
AGGGCCTTCGTGCGTCGCCGCGCCGCCGTCCCCTTCTCCCTCTCCAGCCTCGGGGCTGTCCGCGGGGGGACGGCT
GCCTTCGGGGGGGACGGGGCAGGGCGGGGTTCGGCTTCTGGCGTGTGACCGGCGGCTCTAGAGCCTCTGCTAACC
ATGTTCATGCCTTCTTCTTTTTCCTACAGCTCCTGGGCAACGTGCTGGTTATTGTGCTGTCTCATCATTTTGGCA
AAGAATTATCGCATGCCTGCAGAGCTCTCTGGCTAACTACCGGTGCCACC**ATG**GCCCCAAAGAAGAAGCGGAAGG
TCGGTATCCACGGAGTCCCAGCAGCCAAGCGGAACTACATCCTGGGCCTGGACATCGGCATCACCAGCGTGGGCT
ACGGCATCATCGACTACGAGACACGGGACGTGATCGATGCCGGCGTGCGGCTGTTCAAAGAGGCCAACGTGGAAA
ACAACGAGGGCAGGCGGAGCAAGAGAGGCGCCAGAAGGCTGAAGCGGCGGAGGCGGCATAGAATCCAGAGAGTGA
AGAAGCTGCTGTTCGACTACAACCTGCTGACCGACCACAGCGAGCTGAGCGGCATCAACCCCTACGAGGCCAGAG
TGAAGGGCCTGAGCCAGAAGCTGAGCGAGGAAGAGTTCTCTGCCGCCCTGCTGCACCTGGCCAAGAGAAGAGGCG
TGCACAACGTGAACGAGGTGGAAGAGGACACCGGCAACGAGCTGTCCACCAAAGAGCAGATCAGCCGGAACAGCA
AGGCCCTGGAAGAGAAATACGTGGCCGAACTGCAGCTGGAACGGCTGAAGAAAGACGGCGAAGTGCGGGGCAGCA
TCAACAGATTCAAGACCAGCGACTACGTGAAAGAAGCCAAACAGCTGCTGAAGGTGCAGAAGGCCTACCACCAGC
TGGACCAGAGCTTCATCGACACCTACATCGACCTGCTGGAAACCCGGCGGACCTACTATGAGGGACCTGGCGAGG
GCAGCCCCTTCGGCTGGAAGGACATCAAAGAATGGTACGAGATGCTGATGGGCCACTGCACCTACTTCCCCGAGG
AACTGCGGAGCGTGAAGTACGCCTACAACGCCGACCTGTACAACGCCCTGAACGACCTGAACAATCTCGTGATCA
CCAGGGACGAGAACGAGAAGCTGGAATATTACGAGAAGTTCCAGATCATCGAGAACGTGTTCAAGCAGAAGAAGA
AGCCCACCCTGAAGCAGATCGCCAAAGAAATCCTCGTGAACGAAGAGGATATTAAGGGCTACAGAGTGACCAGCA
CCGGCAAGCCCGAGTTCACCAACCTGAAGGTGTACCACGACATCAAGGACATTACCGCCCGGAAAGAGATTATTG
AGAACGCCGAGCTGCTGGATCAGATTGCCAAGATCCTGACCATCTACCAGAGCAGCGAGGACATCCAGGAAGAAC
TGACCAATCTGAACTCCGAGCTGACCCAGGAAGAGATCGAGCAGATCTCTAATCTGAAGGGCTATACCGGCACCC
ACAACCTGAGCCTGAAGGCCATCAACCTGATCCTGGACGAGCTGTGGCACACCAACGACAACCAGATCGCTATCT
TCAACCGGCTGAAGCTGGTGCCCAAGAAGGTGGACCTGTCCCAGCAGAAAGAGATCCCCACCACCCTGGTGGACG
ACTTCATCCTGAGCCCCGTCGTGAAGAGAAGCTTCATCCAGAGCATCAAAGTGATCAACGCCATCATCAAGAAGT
ACGGCCTGCCCAACGACATCATTATCGAGCTGGCCCGCGAGAAGAACTCCAAGGACGCCCAGAAAATGATCAACG
AGATGCAGAAGCGGAACCGGCAGACCAACGAGCGGATCGAGGAAATCATCCGGACCACCGGCAAAGAGAACGCCA
AGTACCTGATCGAGAAGATCAAGCTGCACGACATGCAGGAAGGCAAGTGCCTGTACAGCCTGGAAGCCATCCCTC
TGGAAGATCTGCTGAACAACCCCTTCAACTATGAGGTGGACCACATCATCCCCAGAAGCGTGTCCTTCGACAACA
GCTTCAACAACAAGGTGCTCGTGAAGCAGGAAGAAAACAGCAAGAAGGGCAACCGGACCCCATTCCAGTACCTGA
GCAGCAGCGACAGCAAGATCAGCTACGAAACCTTCAAGAAGCACATCCTGAATCTGGCCAAGGGCAAGGGCAGAA
TCAGCAAGACCAAGAAAGAGTATCTGCTGGAAGAACGGGACATCAACAGGTTCTCCGTGCAGAAAGACTTCATCA
ACCGGAACCTGGTGGATACCAGATACGCCACCAGAGGCCTGATGAACCTGCTGCGGAGCTACTTCAGAGTGAACA
ACCTGGACGTGAAAGTGAAGTCCATCAATGGCGGCTTCACCAGCTTTCTGCGGCGGAAGTGGAAGTTTAAGAAAG
AGCGGAACAAGGGGTACAAGCACCACGCCGAGGACGCCCTGATCATTGCCAACGCCGATTTCATCTTCAAAGAGT
GGAAGAAACTGGACAAGGCCAAAAAAGTGATGGAAAACCAGATGTTCGAGGAAAAGCAGGCCGAGAGCATGCCCG
AGATCGAAACCGAGCAGGAGTACAAAGAGATCTTCATCACCCCCCACCAGATCAAGCACATTAAGGACTTCAAGG
ACTACAAGTACAGCCACCGGGTGGACAAGAAGCCTAATAGAGAGCTGATTAACGACACCCTGTACTCCACCCGGA
AGGACGACAAGGGCAACACCCTGATCGTGAACAATCTGAACGGCCTGTACGACAAGGACAATGACAAGCTGAAAA
AGCTGATCAACAAGAGCCCCGAAAAGCTGCTGATGTACCACCACGACCCCCAGACCTACCAGAAACTGAAGCTGA
TTATGGAACAGTACGGCGACGAGAAGAATCCCCTGTACAAGTACTACGAGGAAACCGGGAACTACCTGACCAAGT
ACTCCAAAAAGGACAACGGCCCCGTGATCAAGAAGATTAAGTATTACGGCAACAAACTGAACGCCCATCTGGACA
TCACCGACGACTACCCCAACAGCAGAAACAAGGTCGTGAAGCTGTCCCTGAAGCCCTACAGATTCGACGTGTACC
TGGACAATGGCGTGTACAAGTTCGTGACCGTGAAGAATCTGGATGTGATCAAAAAAGAAAACTACTACGAAGTGA
ATAGCAAGTGCTATGAGGAAGCTAAGAAGCTGAAGAAGATCAGCAACCAGGCCGAGTTTATCGCCTCCTTCTACA
ACAACGATCTGATCAAGATCAACGGCGAGCTGTATAGAGTGATCGGCGTGAACAACGACCTGCTGAACCGGATCG
AAGTGAACATGATCGACATCACCTACCGCGAGTACCTGGAAAACATGAACGACAAGAGGCCCCCCAGGATCATTA
AGACAATCGCCTCCAAGACCCAGAGCATTAAGAAGTACAGCACAGACATTCTGGGCAACCTGTATGAAGTGAAAT

CTAAGAAGCACCCTCAGATCATCAAAAAGGGC**AAAAGGCCGGCGGCCACGAAAAAGGCCGGCCAGGCAAAAAAGA**
AAAAGGGA**TCCGGCAGCGAGACTCCCGGGACCTCAGAGTCCGCCACACCCGAAAGT**TTCGAACCCAAGAAGAAGA
GGAAAGTCCCTAGGCCAAAGAAGAAGCGGAAGGTCACC**GGTGGCTCTGGAGATAGAGACGATAGTGATCCATCAG**
ATAAAAATGACGGGAGTGGAGGTAGTGGTACCGACAAGAAGTACAGCATCGGCCTGGACATCGGCACCAACTCTG
TGGGCTGGGCCGTGATCACCGACGAGTACAAGGTGCCCAGCAAGAAATTCAAGGTGCTGGGCAACACCGACCGGC
ACAGCATCAAGAAGAACCTGATCGGAGCCCTGCTGTTCGACAGCGGCGAAACAGCCGAGGCCACCCGGCTGAAGA
GAACCGCCAGAAGAAGATACACCAGACGGAAGAACCGGATCTGCTATCTGCAAGAGATCTTCAGCAACGAGATGG
CCAAGGTGGACGACAGCTTCTTCCACAGACTGGAAGAGTCCTTCCTGGTGGAAGAGGATAAGAAGCACGAGCGGC
ACCCCATCTTCGGCAACATCGTGGACGAGGTGGCCTACCACGAGAAGTACCCCACCATCTACCACCTGAGAAAGA
AACTGGTGGACAGCACCGACAAGGCCGACCTGCGGCTGATCTATCTGGCCCTGGCCCACATGATCAAGTTCCGGG
GCCACTTCCTGATCGAGGGCGACCTGAACCCCGACAACAGCGACGTGGACAAGCTGTTCATCCAGCTGGTGCAGA
CCTACAACCAGCTGTTCGAGGAAAACCCCATCAACGCCAGCGGCGTGGACGCCAAGGCCATCCTGTCTGCCAGAC
TGAGCAAGAGCAGACGGCTGGAAAATCTGATCGCCCAGCTGCCCGGCGAGAAGAAGAATGGCCTGTTCGGAAACC
TGATTGCCCTGAGCCTGGGCCTGACCCCCAACTTCAAGAGCAACTTCGACCTGGCCGAGGATGCCAAACTGCAGC
TGAGCAAGGACACCTACGACGACGACCTGGACAACCTGCTGGCCCAGATCGGCGACCAGTACGCCGACCTGTTTC
TGGCCGCCAAGAACCTGTCCGACGCCATCCTGCTGAGCGACATCCTGAGAGTGAACACCGAGATCACCAAGGCCC
CCCTGAGCGCCTCTATGATCAAGAGATACGACGAGCACCACCAGGACCTGACCCTGCTGAAAGCTCTCGTGCGGC
AGCAGCTGCCTGAGAAGTACAAAGAGATTTTCTTCGACCAGAGCAAGAACGGCTACGCCGGCTACATTGACGGCG
GAGCCAGCCAGGAAGAGTTCTACAAGTTCATCAAGCCCATCCTGGAAAAGATGGACGGCACCGAGGAACTGCTCG
TGAAGCTGAACAGAGAGGACCTGCTGCGGAAGCAGCGGACCTTCGACAACGGCAGCATCCCCCACCAGATCCACC
TGGGAGAGCTGCACGCCATTCTGCGGCGGCAGGAAGATTTTTACCCATTCCTGAAGGACAACCGGGAAAAGATCG
AGAAGATCCTGACCTTCCGCATCCCCTACTACGTGGGCCCTCTGGCCAGGGGAAACAGCAGATTCGCCTGGATGA
CCAGAAAGAGCGAGGAAACCATCACCCCCTGGAACTTCGAGGAAGTGGTGGACAAGGGCGCTTCCGCCCAGAGCT
TCATCGAGCGGATGACCAACTTCGATAAGAACCTGCCCAACGAGAAGGTGCTGCCCAAGCACAGCCTGCTGTACG
AGTACTTCACCGTGTATAACGAGCTGACCAAAGTGAAATACGTGACCGAGGGAATGAGAAAGCCCGCCTTCCTGA
GCGGCGAGCAGAAAAAGGCCATCGTGGACCTGCTGTTCAAGACCAACCGGAAAGTGACCGTGAAGCAGCTGAAAG
AGGACTACTTCAAGAAAATCGAGTGCTTCGACTCCGTGGAAATCTCCGGCGTGGAAGATCGGTTCAACGCCTCCC
TGGGCACATACCACGATCTGCTGAAAATTATCAAGGACAAGGACTTCCTGGACAATGAGGAAAACGAGGACATTC
TGGAAGATATCGTGCTGACCCTGACACTGTTTGAGGACAGAGAGATGATCGAGGAACGGCTGAAAACCTATGCCC
ACCTGTTCGACGACAAAGTGATGAAGCAGCTGAAGCGGCGGAGATACACCGGCTGGGGCAGGCTGAGCCGGAAGC
TGATCAACGGCATCCGGGACAAGCAGTCCGGCAAGACAATCCTGGATTTCCTGAAGTCCGACGGCTTCGCCAACA
GAAACTTCATGCAGCTGATCCACGACGACAGCCTGACCTTTAAAGAGGACATCCAGAAAGCCCAGGTGTCCGGCC
AGGGCGATAGCCTGCACGAGCACATTGCCAATCTGGCCGGCAGCCCCGCCATTAAGAAGGGCATCCTGCAGACAG
TGAAGGTGGTGGACGAGCTCGTGAAAGTGATGGGCCGGCACAAGCCCGAGAACATCGTGATCGAAATGGCCAGAG
AGAACCAGACCACCCAGAAGGGACAGAAGAACAGCCGCGAGAGAATGAAGCGGATCGAAGAGGGCATCAAAGAGC
TGGGCAGCCAGATCCTGAAAGAACACCCCGTGGAAAACACCCAGCTGCAGAACGAGAAGCTGTACCTGTACTACC
TGCAGAATGGGCGGGATATGTACGTGGACCAGGAACTGGACATCAACCGGCTGTCCGACTACGATGTGGACCATA
TCGTGCCTCAGAGCTTTCTGGCCGACGACTCCATCGACAACAAGGTGCTGACCAGAAGCGACAAGAACCGGGGCA
AGAGCGACAACGTGCCCTCCGAAGAGGTCGTGAAGAAGATGAAGAACTACTGGCGGCAGCTGCTGAACGCCAAGC
TGATTACCCAGAGAAAGTTCGACAATCTGACCAAGGCCGAGAGAGGCGGCCTGAGCGAACTGGATAAGGCCGGCT
TCATCAAGAGACAGCTGGTGGAAACCCGGCAGATCACAAAGCACGTGGCACAGATCCTGGACTCCCGGATGAACA
CTAAGTACGACGAGAATGACAAGCTGATCCGGGAAGTGAAAGTGATCACCCTGAAGTCCAAGCTGGTGTCCGATT
TCCGGAAGGATTTCCAGTTTTACAAAGTGCGCGAGATCAACAACTACCACCACGCCCACGACGCCTACCTGAACG
CCGTCGTGGGAACCGCCCTGATCAAAAAGTACCCTGCGCTGGAAAGCGAGTTCGTGTACGGCGACTACAAGGTGT
ACGACGTGCGGAAGATGATCGCCAAGAGCGAGCAGGAAATCGGCAAGGCTACCGCCAAGTACTTCTTCTACAGCA
ACATCATGAACTTTTTCAAGACCGAGATTACCCTGGCCAACGGCGAGATCCGGAAGGCGCCTCTGATCGAGACAA
ACGGCGAAACCGGGGAGATCGTGTGGGATAAGGGCCGGGATTTTGCCACCGTGCGGAAAGTGCTGAGCATGCCCC
AAGTGAATATCGTGAAAAAGACCGAGGTGCAGACAGGCGGCTTCAGCAAAGAGTCTATCCTGCCCAAGAGGAACA
GCGATAAGCTGATCGCCAGAAAGAAGGACTGGGACCCTAAGAAGTACGGCGGCTTCGACAGCCCCACCGTGGCCT
ATTCTGTGCTGGTGGTGGCCAAAGTGGAAAAGGGCAAGTCCAAGAAACTGAAGAGTGTGAAAGAGCTGCTGGGGA
TCACCATCATGGAAAGAAGCAGCTTCGAGAAGAATCCCATCGACTTTCTGGAAGCCAAGGGCTACAAAGAAGTGA
AAAAGGACCTGATCATCAAGCTGCCTAAGTACTCCCTGTTCGAGCTGGAAAACGGCCGGAAGAGAATGCTGGCCT
CTGCCGGCGAACTGCAGAAGGGAAACGAACTGGCCCTGCCCTCCAAATATGTGAACTTCCTGTACCTGGCCAGCC
ACTATGAGAAGCTGAAGGGCTCCCCCGAGGATAATGAGCAGAAACAGCTGTTTGTGGAACAGCACAAGCACTACC
TGGACGAGATCATCGAGCAGATCAGCGAGTTCTCCAAGAGAGTGATCCTGGCCGACGCTAATCTGGACAAAGTGC
TGTCCGCCTACAACAAGCACCGGGATAAGCCCATCAGAGAGCAGGCCGAGAATATCATCCACCTGTTTACCCTGA
CCAATCTGGGAGCCCCTGCCGCCTTCAAGTACTTTGACACCACCATCGACCGGAAGAGGTACACCAGCACCAAAG

AGGTGCTGGACGCCACCCTGATCCACCAGAGCATCACCGGCCTGTACGAGACACGGATCGACCTGTCTCAGCTGG
GAGGCGACAAAAGGCCGGCGGCCACGAAAAAGGCCGGCCAGGCAAAAAAGAAAAAGGCTAGCGGCTCCCCCAAGA
AAAAACGCAAGGTGGAAGATCCTAAGAAAAAGCGGAAAGTGTAAGAATTCCCTGCAGGACGCGTGGAAATTCACT
CCTCAGGTGCAGGCTGCCTATCAGAAGGTGGTGGCTGGTGTGGCCAATGCCCTGGCTCACAAATACCACTGAGAT
CTTTTTCCCTCTGCCAAAAATTATGGGGACATCATGAAGCCCCTTGAGCATCTGACTTCTGGCTAATAAAGGAAA
TTTATTTTCATTGCAATAGTGTGTTGGAATTTTTTGTGTCTCTCA

Figure S10. Map and sequence of expression unit in AW78_pHC-Ad.CAG.SaCas9.link.eCas9.4NLS. CAG, hybrid promoter composed of the human cytomegalovirus *immediate-early* enhancer, the chicken *β-actin* promoter and a chimeric intron formed by chicken *β-actin* and rabbit *β-globin* sequences; SaCas9, Cas9 endonuclease derived from the *Staphylococcus aureus* Type II CRISPR-Cas system; High-specificity SpCas9, optimized variant of eSpCas9(1.1) derived from the *Streptococcus pyogenes* Type II CRISPR-Cas system. The point mutations K848A, K1003A and R1060A, conferring enhanced specificity, are marked (vertical cyan lines); SV40 NLS, nuclear localization signal motif from the simian virus 40 large T antigen; Nucleoplasmin NLS, nucleoplasmin nuclear localization signal; XTEN linker and Linker, DNA coding for flexible peptide linkers; Poly(A), rabbit *β-globin* polyadenylation signal. The different features are color coded in the expression unit map and respective nucleotide sequence.

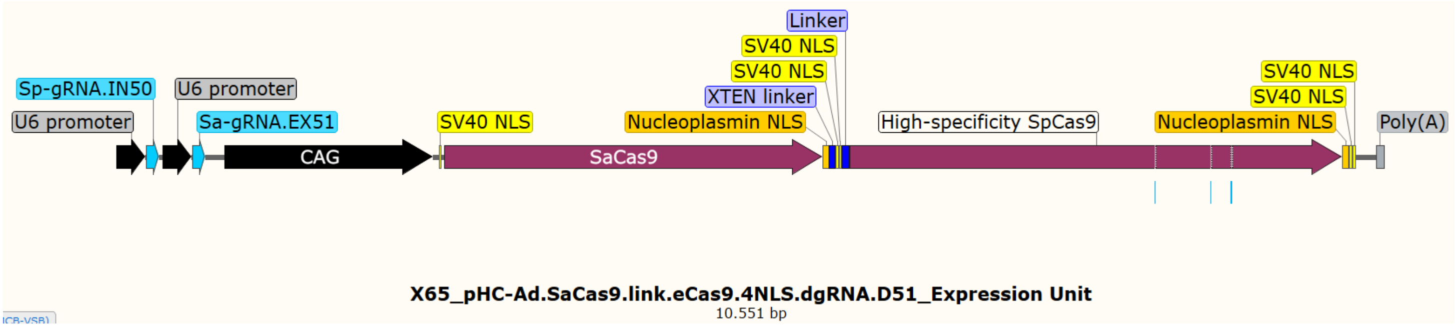

GAGGGCCTATTTCCCATGATTCCTTCATATTTGCATATACGATACAAGGCTGTTAGAGAGATAATTGGAATTAAT
TTGACTGTAAACACAAAGATATTAGTACAAAATACGTGACGTAGAAAGTAATAATTTCTTGGGTAGTTTGCAGTT
TTAAAATTATGTTTTAAAATGGACTATCATATGCTTACCGTAACTTGAAAGTATTTCGATTTCTTGGCTTTATAT
ATCTTGTGGAAAGGACGAAACACC**GATACTTTGTTTAGCAATACA**GTTTCAGAGCTATGCTGGAAACAGCATAGC
AAGTTGAAATAAGGCTAGTCCGTTATCAACTTGAAAAAGTGGCACCGAGTCGGTGCTTTTTTTGAATTCGGTACC
AAGGTCGGGCAGGAAGAGGGCCTATTTCCCATGATTCCTTCATATTTGCATATACGATACAAGGCTGTTAGAGAG
ATAATTAGAATTAATTTGACTGTAAACACAAAGATATTAGTACAAAATACGTGACGTAGAAAGTAATAATTTCTT
GGGTAGTTTGCAGTTTTAAAATTATGTTTTAAAATGGACTATCATATGCTTACCGTAACTTGAAAGTATTTCGAT
TTCTTGGCTTTATATATCTTGTGGAAAGGACGAAACACC**GTTGTGTCACCAGAGTAACAGT**GTTTTAGTACTCTG
TAATGAAAATTACAGAATCTACTAAAACAAGGCAAAATGCCGTGTTTATCTCGTCAACTTGTTGGCGAGATTTTT
TTAAGCTTGGGCCGCTCGAGGTACCGGCGCGCCCGTACGACTAGTATTACCCTGTTATCCCTAGCGGCCGCTTGG
CCGCACTTAAGTTACGCGTACGTCGCGACCGCGGACATGTACAGAGCTCGAGAAGAAACATTTAAATCTCGAGCC
ATGGATTCGACATTGATTATTGACTAGTTATTAATAGTAATCAATTACGGGGTCATTAGTTCATAGCCCATATAT
GGAGTTCCGCGTTACATAACTTACGGTAAATGGCCCGCCTGGCTGACCGCCCAACGACCCCCGCCCATTGACGTC
AATAATGACGTATGTTCCCATAGTAACGCCAATAGGGACTTTCCATTGACGTCAATGGGTGGAGTATTTACGGTA
AACTGCCCACTTGGCAGTACATCAAGTGTATCATATGCCAAGTACGCCCCCTATTGACGTCAATGACGGTAAATG
GCCCGCCTGGCATTATGCCCAGTACATGACCTTATGGGACTTTCCTACTTGGCAGTACATCTACGTATTAGTCAT
CGCTATTACCATGGTCGAGGTGAGCCCCACGTTCTGCTTCACTCTCCCCATCTCCCCCCCCTCCCCACCCCCAAT
TTTGTATTTATTTATTTTTTAATTATTTTGTGCAGCGATGGGGGCGGGGGGGGGGGGGGGGCGCGCGCCAGGCGG
GGCGGGGCGGGGCGAGGGGCGGGGCGGGGCGAGGCGGAGAGGTGCGGCGGCAGCCAATCAGAGCGGCGCGCTCCG
AAAGTTTCCTTTTATGGCGAGGCGGCGGCGGCGGCGGCCCTATAAAAAGCGAAGCGCGCGGCGGGCGGGAGTCGC

TGCGACGCTGCCTTCGCCCCGTGCCCCGCTCCGCCGCCGCCTCGCGCCGCCCGCCCCGGCTCTGACTGACCGCGT
TACTCCCACAGGTGAGCGGGCGGGACGGCCCTTCTCCTCCGGGCTGTAATTAGCGCTTGGTTTAATGACGGCTTG
TTTCTTTTCTGTGGCTGCGTGAAAGCCTTGAGGGGCTCCGGGAGGGCCCTTTGTGCGGGGGGAGCGGCTCGGGG
GTGCGTGCGTGTGTGTGTGCGTGGGGAGCGCCGCGTGCGGCTCCGCGCTGCCCGGCGGCTGTGAGCGCTGCGGGC
GCGGCGCGGGGCTTTGTGCGCTCCGCAGTGTGCGCGAGGGGAGCGCGGCCGGGGGCGGTGCCCCGCGGTGCGGGG
GGGGCTGCGAGGGGAACAAAGGCTGCGTGCGGGGTGTGTGCGTGGGGGGGTGAGCAGGGGGTGTGGGCGCGTCGG
TCGGGCTGCAACCCCCCCTGCACCCCCCTCCCCGAGTTGCTGAGCACGGCCCGGCTTCGGGTGCGGGGCTCCGTA
CGGGGCGTGGCGCGGGGCTCGCCGTGCCGGGCGGGGGGTGGCGGCAGGTGGGGGTGCCGGGCGGGGCGGGGCCGC
CTCGGGCCGGGGAGGGCTCGGGGGAGGGGCGCGGCGGCCCCCGGAGCGCCGGCGGCTGTCGAGGCGCGGCGAGCC
GCAGCCATTGCCTTTTATGGTAATCGTGCGAGAGGGCGCAGGGACTTCCTTTGTCCCAAATCTGTGCGGAGCCGA
AATCTGGGAGGCGCCGCCGCACCCCCTCTAGCGGGCGCGGGGCGAAGCGGTGCGGCGCCGGCAGGAAGGAAATGG
GCGGGGAGGGCCTTCGTGCGTCGCCGCGCCGCCGTCCCCTTCTCCCTCTCCAGCCTCGGGGCTGTCCGCGGGGGG
ACGGCTGCCTTCGGGGGGGACGGGGCAGGGCGGGGTTCGGCTTCTGGCGTGTGACCGGCGGCTCTAGAGCCTCTG
CTAACCATGTTCATGCCTTCTTCTTTTTCCTACAGCTCCTGGGCAACGTGCTGGTTATTGTGCTGTCTCATCATT
TTGGCAAAGAATTATCGCATGCCTGCAGAGCTCTCTGGCTAACTACCGGTGCCACC**ATG**GCCCCAAAGAAGAAGC
GGAAGGTCGGTATCCACGGAGTCCCAGCAGCCAAGCGGAACTACATCCTGGGCCTGGACATCGGCATCACCAGCG
TGGGCTACGGCATCATCGACTACGAGACACGGGACGTGATCGATGCCGGCGTGCGGCTGTTCAAAGAGGCCAACG
TGGAAAACAACGAGGGCAGGCGGAGCAAGAGAGGCGCCAGAAGGCTGAAGCGGCGGAGGCGGCATAGAATCCAGA
GAGTGAAGAAGCTGCTGTTCGACTACAACCTGCTGACCGACCACAGCGAGCTGAGCGGCATCAACCCCTACGAGG
CCAGAGTGAAGGGCCTGAGCCAGAAGCTGAGCGAGGAAGAGTTCTCTGCCGCCCTGCTGCACCTGGCCAAGAGAA
GAGGCGTGCACAACGTGAACGAGGTGGAAGAGGACACCGGCAACGAGCTGTCCACCAAAGAGCAGATCAGCCGGA
ACAGCAAGGCCCTGGAAGAGAAATACGTGGCCGAACTGCAGCTGGAACGGCTGAAGAAAGACGGCGAAGTGCGGG
GCAGCATCAACAGATTCAAGACCAGCGACTACGTGAAAGAAGCCAAACAGCTGCTGAAGGTGCAGAAGGCCTACC
ACCAGCTGGACCAGAGCTTCATCGACACCTACATCGACCTGCTGGAAACCCGGCGGACCTACTATGAGGGACCTG
GCGAGGGCAGCCCCTTCGGCTGGAAGGACATCAAAGAATGGTACGAGATGCTGATGGGCCACTGCACCTACTTCC
CCGAGGAACTGCGGAGCGTGAAGTACGCCTACAACGCCGACCTGTACAACGCCCTGAACGACCTGAACAATCTCG
TGATCACCAGGGACGAGAACGAGAAGCTGGAATATTACGAGAAGTTCCAGATCATCGAGAACGTGTTCAAGCAGA
AGAAGAAGCCCACCCTGAAGCAGATCGCCAAAGAAATCCTCGTGAACGAAGAGGATATTAAGGGCTACAGAGTGA
CCAGCACCGGCAAGCCCGAGTTCACCAACCTGAAGGTGTACCACGACATCAAGGACATTACCGCCCGGAAAGAGA
TTATTGAGAACGCCGAGCTGCTGGATCAGATTGCCAAGATCCTGACCATCTACCAGAGCAGCGAGGACATCCAGG
AAGAACTGACCAATCTGAACTCCGAGCTGACCCAGGAAGAGATCGAGCAGATCTCTAATCTGAAGGGCTATACCG
GCACCCACAACCTGAGCCTGAAGGCCATCAACCTGATCCTGGACGAGCTGTGGCACACCAACGACAACCAGATCG
CTATCTTCAACCGGCTGAAGCTGGTGCCCAAGAAGGTGGACCTGTCCCAGCAGAAAGAGATCCCCACCACCCTGG
TGGACGACTTCATCCTGAGCCCCGTCGTGAAGAGAAGCTTCATCCAGAGCATCAAAGTGATCAACGCCATCATCA
AGAAGTACGGCCTGCCCAACGACATCATTATCGAGCTGGCCCGCGAGAAGAACTCCAAGGACGCCCAGAAAATGA
TCAACGAGATGCAGAAGCGGAACCGGCAGACCAACGAGCGGATCGAGGAAATCATCCGGACCACCGGCAAAGAGA
ACGCCAAGTACCTGATCGAGAAGATCAAGCTGCACGACATGCAGGAAGGCAAGTGCCTGTACAGCCTGGAAGCCA
TCCCTCTGGAAGATCTGCTGAACAACCCCTTCAACTATGAGGTGGACCACATCATCCCCAGAAGCGTGTCCTTCG
ACAACAGCTTCAACAACAAGGTGCTCGTGAAGCAGGAAGAAAACAGCAAGAAGGGCAACCGGACCCCATTCCAGT
ACCTGAGCAGCAGCGACAGCAAGATCAGCTACGAAACCTTCAAGAAGCACATCCTGAATCTGGCCAAGGGCAAGG
GCAGAATCAGCAAGACCAAGAAAGAGTATCTGCTGGAAGAACGGGACATCAACAGGTTCTCCGTGCAGAAAGACT
TCATCAACCGGAACCTGGTGGATACCAGATACGCCACCAGAGGCCTGATGAACCTGCTGCGGAGCTACTTCAGAG
TGAACAACCTGGACGTGAAAGTGAAGTCCATCAATGGCGGCTTCACCAGCTTTCTGCGGCGGAAGTGGAAGTTTA
AGAAAGAGCGGAACAAGGGGTACAAGCACCACGCCGAGGACGCCCTGATCATTGCCAACGCCGATTTCATCTTCA
AAGAGTGGAAGAAACTGGACAAGGCCAAAAAAGTGATGGAAAACCAGATGTTCGAGGAAAAGCAGGCCGAGAGCA
TGCCCGAGATCGAAACCGAGCAGGAGTACAAAGAGATCTTCATCACCCCCCACCAGATCAAGCACATTAAGGACT
TCAAGGACTACAAGTACAGCCACCGGGTGGACAAGAAGCCTAATAGAGAGCTGATTAACGACACCCTGTACTCCA
CCCGGAAGGACGACAAGGGCAACACCCTGATCGTGAACAATCTGAACGGCCTGTACGACAAGGACAATGACAAGC
TGAAAAAGCTGATCAACAAGAGCCCCGAAAAGCTGCTGATGTACCACCACGACCCCCAGACCTACCAGAAACTGA
AGCTGATTATGGAACAGTACGGCGACGAGAAGAATCCCCTGTACAAGTACTACGAGGAAACCGGGAACTACCTGA
CCAAGTACTCCAAAAAGGACAACGGCCCCGTGATCAAGAAGATTAAGTATTACGGCAACAAACTGAACGCCCATC
TGGACATCACCGACGACTACCCCAACAGCAGAAACAAGGTCGTGAAGCTGTCCCTGAAGCCCTACAGATTCGACG
TGTACCTGGACAATGGCGTGTACAAGTTCGTGACCGTGAAGAATCTGGATGTGATCAAAAAAGAAAACTACTACG
AAGTGAATAGCAAGTGCTATGAGGAAGCTAAGAAGCTGAAGAAGATCAGCAACCAGGCCGAGTTTATCGCCTCCT
TCTACAACAACGATCTGATCAAGATCAACGGCGAGCTGTATAGAGTGATCGGCGTGAACAACGACCTGCTGAACC
GGATCGAAGTGAACATGATCGACATCACCTACCGCGAGTACCTGGAAAACATGAACGACAAGAGGCCCCCCAGGA
TCATTAAGACAATCGCCTCCAAGACCCAGAGCATTAAGAAGTACAGCACAGACATTCTGGGCAACCTGTATGAAG

TGAAATCTAAGAAGCACCCTCAGATCATCAAAAAGGGC**AAAAGGCCGGCGGCCACGAAAAAGGCCGGCCAGGCAA**
AAAAGAAAAAGGGA**TCCGGCAGCGAGACTCCCGGACCTCAGAGTCCGCCACACCCGAAAGT**TTCGAACCCAAGA
AGAAGAGGAAAGTCCCTAGGCCAAAGAAGAAGCGGAAGGTCACC**GGTGGCTCTGGAGATAGAGACGATAGTGATC**
CATCAGATAAAAATGACGGGAGTGGAGGTAGTGGTACCGACAAGAAGTACAGCATCGGCCTGGACATCGGCACCA
ACTCTGTGGGCTGGGCCGTGATCACCGACGAGTACAAGGTGCCCAGCAAGAAATTCAAGGTGCTGGGCAACACCG
ACCGGCACAGCATCAAGAAGAACCTGATCGGAGCCCTGCTGTTCGACAGCGGCGAAACAGCCGAGGCCACCCGGC
TGAAGAGAACCGCCAGAAGAAGATACACCAGACGGAAGAACCGGATCTGCTATCTGCAAGAGATCTTCAGCAACG
AGATGGCCAAGGTGGACGACAGCTTCTTCCACAGACTGGAAGAGTCCTTCCTGGTGGAAGAGGATAAGAAGCACG
AGCGGCACCCCATCTTCGGCAACATCGTGGACGAGGTGGCCTACCACGAGAAGTACCCCACCATCTACCACCTGA
GAAAGAAACTGGTGGACAGCACCGACAAGGCCGACCTGCGGCTGATCTATCTGGCCCTGGCCCACATGATCAAGT
TCCGGGGCCACTTCCTGATCGAGGGCGACCTGAACCCCGACAACAGCGACGTGGACAAGCTGTTCATCCAGCTGG
TGCAGACCTACAACCAGCTGTTCGAGGAAAACCCCATCAACGCCAGCGGCGTGGACGCCAAGGCCATCCTGTCTG
CCAGACTGAGCAAGAGCAGACGGCTGGAAAATCTGATCGCCCAGCTGCCCGGCGAGAAGAAGAATGGCCTGTTCG
GAAACCTGATTGCCCTGAGCCTGGGCCTGACCCCCAACTTCAAGAGCAACTTCGACCTGGCCGAGGATGCCAAAC
TGCAGCTGAGCAAGGACACCTACGACGACGACCTGGACAACCTGCTGGCCCAGATCGGCGACCAGTACGCCGACC
TGTTTCTGGCCGCCAAGAACCTGTCCGACGCCATCCTGCTGAGCGACATCCTGAGAGTGAACACCGAGATCACCA
AGGCCCCCCTGAGCGCCTCTATGATCAAGAGATACGACGAGCACCACCAGGACCTGACCCTGCTGAAAGCTCTCG
TGCGGCAGCAGCTGCCTGAGAAGTACAAAGAGATTTTCTTCGACCAGAGCAAGAACGGCTACGCCGGCTACATTG
ACGGCGGAGCCAGCCAGGAAGAGTTCTACAAGTTCATCAAGCCCATCCTGGAAAAGATGGACGGCACCGAGGAAC
TGCTCGTGAAGCTGAACAGAGAGGACCTGCTGCGGAAGCAGCGGACCTTCGACAACGGCAGCATCCCCCACCAGA
TCCACCTGGGAGAGCTGCACGCCATTCTGCGGCGGCAGGAAGATTTTTACCCATTCCTGAAGGACAACCGGGAAA
AGATCGAGAAGATCCTGACCTTCCGCATCCCCTACTACGTGGGCCCTCTGGCCAGGGGAAACAGCAGATTCGCCT
GGATGACCAGAAAGAGCGAGGAAACCATCACCCCCTGGAACTTCGAGGAAGTGGTGGACAAGGGCGCTTCCGCCC
AGAGCTTCATCGAGCGGATGACCAACTTCGATAAGAACCTGCCCAACGAGAAGGTGCTGCCCAAGCACAGCCTGC
TGTACGAGTACTTCACCGTGTATAACGAGCTGACCAAAGTGAAATACGTGACCGAGGGAATGAGAAAGCCCGCCT
TCCTGAGCGGCGAGCAGAAAAAGGCCATCGTGGACCTGCTGTTCAAGACCAACCGGAAAGTGACCGTGAAGCAGC
TGAAAGAGGACTACTTCAAGAAAATCGAGTGCTTCGACTCCGTGGAAATCTCCGGCGTGGAAGATCGGTTCAACG
CCTCCCTGGGCACATACCACGATCTGCTGAAAATTATCAAGGACAAGGACTTCCTGGACAATGAGGAAAACGAGG
ACATTCTGGAAGATATCGTGCTGACCCTGACACTGTTTGAGGACAGAGAGATGATCGAGGAACGGCTGAAAACCT
ATGCCCACCTGTTCGACGACAAAGTGATGAAGCAGCTGAAGCGGCGGAGATACACCGGCTGGGGCAGGCTGAGCC
GGAAGCTGATCAACGGCATCCGGGACAAGCAGTCCGGCAAGACAATCCTGGATTTCCTGAAGTCCGACGGCTTCG
CCAACAGAAACTTCATGCAGCTGATCCACGACGACAGCCTGACCTTTAAAGAGGACATCCAGAAAGCCCAGGTGT
CCGGCCAGGGCGATAGCCTGCACGAGCACATTGCCAATCTGGCCGGCAGCCCCGCCATTAAGAAGGGCATCCTGC
AGACAGTGAAGGTGGTGGACGAGCTCGTGAAAGTGATGGGCCGGCACAAGCCCGAGAACATCGTGATCGAAATGG
CCAGAGAGAACCAGACCACCCAGAAGGGACAGAAGAACAGCCGCGAGAGAATGAAGCGGATCGAAGAGGGCATCA
AAGAGCTGGGCAGCCAGATCCTGAAAGAACACCCCGTGGAAAACACCCAGCTGCAGAACGAGAAGCTGTACCTGT
ACTACCTGCAGAATGGGCGGGATATGTACGTGGACCAGGAACTGGACATCAACCGGCTGTCCGACTACGATGTGG
ACCATATCGTGCCTCAGAGCTTTCTGGCCGACGACTCCATCGACAACAAGGTGCTGACCAGAAGCGACAAGAACC
GGGGCAAGAGCGACAACGTGCCCTCCGAAGAGGTCGTGAAGAAGATGAAGAACTACTGGCGGCAGCTGCTGAACG
CCAAGCTGATTACCCAGAGAAAGTTCGACAATCTGACCAAGGCCGAGAGAGGCGGCCTGAGCGAACTGGATAAGG
CCGGCTTCATCAAGAGACAGCTGGTGGAAACCCGGCAGATCACAAAGCACGTGGCACAGATCCTGGACTCCCGGA
TGAACACTAAGTACGACGAGAATGACAAGCTGATCCGGGAAGTGAAAGTGATCACCCTGAAGTCCAAGCTGGTGT
CCGATTTCCGGAAGGATTTCCAGTTTTACAAAGTGCGCGAGATCAACAACTACCACCACGCCCACGACGCCTACC
TGAACGCCGTCGTGGGAACCGCCCTGATCAAAAAGTACCCTGCGCTGGAAAGCGAGTTCGTGTACGGCGACTACA
AGGTGTACGACGTGCGGAAGATGATCGCCAAGAGCGAGCAGGAAATCGGCAAGGCTACCGCCAAGTACTTCTTCT
ACAGCAACATCATGAACTTTTTCAAGACCGAGATTACCCTGGCCAACGGCGAGATCCGGAAGGCGCCTCTGATCG
AGACAAACGGCGAAACCGGGGAGATCGTGTGGGATAAGGGCCGGGATTTTGCCACCGTGCGGAAAGTGCTGAGCA
TGCCCCAAGTGAATATCGTGAAAAAGACCGAGGTGCAGACAGGCGGCTTCAGCAAAGAGTCTATCCTGCCCAAGA
GGAACAGCGATAAGCTGATCGCCAGAAAGAAGGACTGGGACCCTAAGAAGTACGGCGGCTTCGACAGCCCCACCG
TGGCCTATTCTGTGCTGGTGGTGGCCAAAGTGGAAAAGGGCAAGTCCAAGAAACTGAAGAGTGTGAAAGAGCTGC
TGGGGATCACCATCATGGAAAGAAGCAGCTTCGAGAAGAATCCCATCGACTTTCTGGAAGCCAAGGGCTACAAAG
AAGTGAAAAAGGACCTGATCATCAAGCTGCCTAAGTACTCCCTGTTCGAGCTGGAAAACGGCCGGAAGAGAATGC
TGGCCTCTGCCGGCGAACTGCAGAAGGGAAACGAACTGGCCCTGCCCTCCAAATATGTGAACTTCCTGTACCTGG
CCAGCCACTATGAGAAGCTGAAGGGCTCCCCCGAGGATAATGAGCAGAAACAGCTGTTTGTGGAACAGCACAAGC
ACTACCTGGACGAGATCATCGAGCAGATCAGCGAGTTCTCCAAGAGAGTGATCCTGGCCGACGCTAATCTGGACA
AAGTGCTGTCCGCCTACAACAAGCACCGGGATAAGCCCATCAGAGAGCAGGCCGAGAATATCATCCACCTGTTTA
CCCTGACCAATCTGGGAGCCCCTGCCGCCTTCAAGTACTTTGACACCACCATCGACCGGAAGAGGTACACCAGCA

CCAAAGAGGTGCTGGACGCCACCCTGATCCACCAGAGCATCACCGGCCTGTACGAGACACGGATCGACCTGTCTC
AGCTGGGAGGCGAC**AAAAGGCCGGCGGCCACGAAAAAGGCCGGCCAGGCAAAAAAGAAAAAG**GCTAGCGGCTCCC
CCAAGAAAAAACGCAAGGTGGAAGATCCTAAGAAAAAGCGGAAAGTGTAAGAATTCCCTGCAGGACGCGTGGAAA
TTCACTCCTCAGGTGCAGGCTGCCTATCAGAAGGTGGTGGCTGGTGTGGCCAATGCCCTGGCTCACAAATACCAC
TGAGATCTTTTTCCCTCTGCCAAAAATTATGGGGACATCATGAAGCCCCTTGAGCATCTGACTTCTGGCTAATAA
AGGAAATTTATTTTCATTGCAATAGTGTGTTGGAATTTTTTGTGTCTCTCA

Figure S11. Map and sequence of expression unit in X65_pHC-Ad.gSp^{IN50}.gSa^{EX51}.SaCas9.link.eCas9.4NLS.

CAG, hybrid promoter composed of the human cytomegalovirus *immediate-early* enhancer, the chicken *β-actin* promoter and a chimeric intron formed by chicken *β-actin* and rabbit *β-globin* sequences; SaCas9, Cas9 endonuclease derived from the *Staphylococcus aureus* Type II CRISPR-Cas system; High-specificity SpCas9, optimized variant of eSpCas9(1.1) derived from the *Streptococcus pyogenes* Type II CRISPR-Cas system. The point mutations K848A, K1003A and R1060A, conferring enhanced specificity, are indicated (vertical cyan lines); U6, RNA polymerase III promoter for human snRNA; Sp-gRNA.IN50 and Sa-gRNA.EX51, *Staphylococcus aureus* and *Streptococcus pyogenes* gRNAs with spacers (underlined) targeting *DMD* intron 50 and exon 51 sequences, respectively. SV40 NLS, nuclear localization signal motif from the simian virus 40 large T antigen; Nucleoplasmin NLS, nucleoplasmin nuclear localization signal; XTEN linker and Linker, DNA coding for flexible peptide linkers; Poly(A), rabbit *β-globin* polyadenylation signal. The different elements are color coded in the expression unit map and respective nucleotide sequence.

```
cutadapt -a agatcggaagagcacacg -A ctgtctcttatacacatc -o out_R1.fastq
-p out_R2.fastq reads_R1.fastq reads_R2.fastq
```

Figure S12. The script for adapter trimming of raw NGS fastq reads. The script supports trimming of paired-end reads with Cutadapt 2.10. The output files and input reads are highlighted in blue and red, respectively.

Set 1

```
#!/bin/bash
for fl in *_R1_001.fastq.gz
do
  SAMPLE=$(echo ${fl} | sed "s/_R1_\001\.fastq\.gz//")
echo ${SAMPLE}_R1_001.fastq.gz ${SAMPLE}_R2_001.fastq.gz
CRISPResso --fastq_r1 ${SAMPLE}_R1_001.fastq.gz --fastq_r2 ${SAMPLE}_R2_001.fastq.gz -g
CTCGTGACCACCCTGACCTA,GCAACATCCTGGGGCACAAGC -w 0 --amplicon_seq
GGGCTTGATGACGTTCTCAGTGCTATCCATGGTGGCGACCGGTACTCCAGCAGTCAGGGTGGTCACGAGGGTTGAA
TTcgagctctgcttatatagacctcccaccgtacacgcctaccgcccatttgcttcaatggggcggagttgttac,GACGTTCTCAGTGCTATCCA
TGGTGGCGACCGGTACTCCAGCAACATCCTGGGGCACAAGCTGGAGTTGTACATTACTTATTTAAACTTGTTTATTG
CAGCTTATAATGGTTACAAATAAAGCAATAGCATCACAAATTTCACAAATAAAGCATTTTTTTCACTGCATTCTAGTT
GTGGTTTGTCCAAACTCATCAATGTATCTTATCATTCTAGAGCCGTAGGTCAGGGTGGTCACGAGGGTTGAATTCGA
GCTCTGCTTATATAGACCTCCCACCGTACACGCCTACCGCCCATTTGCTTCAATGGGGCGGA -an Del,Ref --
exclude_bp_from_left 30 --exclude_bp_from_right 30 --max_paired_end_reads_overlap 368 -amas 40 --
ignore_substitutions --trim_sequences --trimmomatic_options_string
"ILLUMINACLIP:adapter.fa:0:90:10:0:true LEADING:25 TRAILING:25"\

done
```

Set 2

```
#!/bin/bash
for fl in *_R1_001.fastq.gz
do
  SAMPLE=$(echo ${fl} | sed "s/_R1_\001\.fastq\.gz//")
echo ${SAMPLE}_R1_001.fastq.gz ${SAMPLE}_R2_001.fastq.gz
CRISPResso --fastq_r1 ${SAMPLE}_R1_001.fastq.gz --fastq_r2
${SAMPLE}_R2_001.fastq.gz -g CTCGTGACCACCCTGACCTA,gcttgtgccccaggatgttgc -w
0 --amplicon_seq
GGGCTTGATGACGTTCTCAGTGCTATCCATGGTGGCGACCGGTACTCCAGCAGTCAGGGTGGTCACGAGGGTTGA
ATTcgagctctgcttatatagacctcccaccgtacacgcctaccgcccatttgcttcaatggggcggagttgtta
c,GACGTTCTCAGTGCTATCCATGGTGGCGACCGGTACTCCAGCAACATCCTGGGGCACAAGCTGGAGTTGTACA
TTACTTATTTAAACTTGTTTATTGCAGCTTATAATGGTTACAAATAAAGCAATAGCATCACAAATTTCACAAATA
AAGCATTTTTTTCACTGCATTCTAGTTGTGGTTTGTCCAAACTCATCAATGTATCTTATCATTCTAGAGCCGTAG
GTCAGGGTGGTCACGAGGGTTGAATTCGAGCTCTGCTTATATAGACCTCCCACCGTACACGCCTACCGCCCATTT
GCTTCAATGGGGCGGA -an Del,Ref --exclude_bp_from_left 30 --
exclude_bp_from_right 30 --max_paired_end_reads_overlap 368 -amas 40 --
ignore_substitutions --trim_sequences --trimmomatic_options_string
"ILLUMINACLIP:adapter.fa:0:90:10:0:true LEADING:25 TRAILING:25"\

done
```

Set 3

```
#!/bin/bash
for fl in *_R1_001.fastq.gz
```

```
do
  SAMPLE=$(echo ${fl} | sed "s/_R1_\001\.fastq\.gz//")
echo ${SAMPLE}_R1_001.fastq.gz ${SAMPLE}_R2_001.fastq.gz
CRISPResso --fastq_r1 ${SAMPLE}_R1_001.fastq.gz --fastq_r2
${SAMPLE}_R2_001.fastq.gz -g gcaacatcctggggcacaagc,gtaggtcagggtggtcacga
-w 0 --amplicon_seq
GGGCTTGATGACGTTCTCAGTGCTATCCATGGTGGCGACCGGTACTCCAGCAACATCCTGGGGCACACGAG
GGTTGAATTcgagctctgcttatatagacctcccaccgtacacgcctaccgcccatttgcttcaatggggc
ggagttgttac,GACGTTCTCAGTGCTATCCATGGTGGCGACCGGTACTCCAGCAACATCCTGGGGCACAA
GCTGGAGTTGTACATTACTTATTTAAACTTGTTTATTGCAGCTTATAATGGTTACAAATAAAGCAATAGCA
TCACAAATTTCACAAATAAAGCATTTTTTTCACTGCATTCTAGTTGTGGTTTGTCCAAACTCATCAATGTA
TCTTATCATTCTAGAGCCGTAGGTCAGGGTGGTCACGAGGGTTGAATTCGAGCTCTGCTTATATAGACCTC
CCACCGTACACGCCTACCGCCCATTTGCTTCAATGGGGCGGA -an Del,Ref --
exclude_bp_from_left 30 --exclude_bp_from_right 30 --
max_paired_end_reads_overlap 368 -amas 40 --ignore_substitutions --
trim_sequences --trimmomatic_options_string
"ILLUMINACLIP:adapter.fa:0:90:10:0:true LEADING:25 TRAILING:25"\

done
```

Set 4

```
#!/bin/bash
for fl in *_R1_001.fastq.gz
do
  SAMPLE=$(echo ${fl} | sed "s/_R1_\001\.fastq\.gz//")
echo ${SAMPLE}_R1_001.fastq.gz ${SAMPLE}_R2_001.fastq.gz
CRISPResso     --fastq_r1     ${SAMPLE}_R1_001.fastq.gz     --fastq_r2
${SAMPLE}_R2_001.fastq.gz -g gtaggtcagggtggtcacga,gcttgtgccccaggatgttgc
-w                     0                       --amplicon_seq
GGGCTTGATGACGTTCTCAGTGCTATCCATGGTGGCGACCGGTACTCCAGCAACATCCTGGGGCACAGTCA
GGGTGGTCACGAGGGTTGAATTCGAGCTCTGCTTATATAGACCTCCCACCGTACACGCCTACCGCCCATTT
GCTTCAATGGGGCGGAGTTGTTAC,GACGTTCTCAGTGCTATCCATGGTGGCGACCGGTACTCCAGCAACA
TCCTGGGGCACAAGCTGGAGTTGTACATTACTTATTTAAACTTGTTTATTGCAGCTTATAATGGTTACAAA
TAAAGCAATAGCATCACAAATTTCACAAATAAAGCATTTTTTTCACTGCATTCTAGTTGTGGTTTGTCCAA
ACTCATCAATGTATCTTATCATTCTAGAGCCGTAGGTCAGGGTGGTCACGAGGGTTGAATTCGAGCTCTGC
TTATATAGACCTCCCACCGTACACGCCTACCGCCCATTTGCTTCAATGGGGCGGA -an Del,Ref --
exclude_bp_from_left     30     --exclude_bp_from_right     30     --
max_paired_end_reads_overlap 368 -amas 40 --ignore_substitutions --
trim_sequences                       --trimmomatic_options_string
"ILLUMINACLIP:adapter.fa:0:90:10:0:true LEADING:25 TRAILING:25"\

done
```

Figure S13. Scripts for characterize genome editing outcomes in fluorescent-based reporter cell line HeLa.DsRed$^{TS.An.TS}$ transduced with AdVP.SpC9 and AdVP.SaC9 or AdV.SpC9::SaC9. The scripts are used for CRISPResso2 analysis via the Docker containerization systems. The scripts to analyses samples derived trough Set 1, Set 2, Set 3 and Set 4 are listed. The result of this analysis is presented in Figure 1.

```
#!/bin/bash
for fl in *_R1_001.fastq.gz
do
```

```
  SAMPLE=$(echo ${fl} | sed "s/_R1_\001\.fastq\.gz//")
echo ${SAMPLE}_R1_001.fastq.gz ${SAMPLE}_R2_001.fastq.gz
CRISPResso --fastq_r1 ${SAMPLE}_R1_001.fastq.gz --fastq_r2
${SAMPLE}_R2_001.fastq.gz -g
GTTGTGTCACCAGAGTAACAGT,GATACTTTGTTTAGCAATACA --amplicon_seq
GTACCTCCAACATCAAGGAAGATGGCATTTCTAGTTTGGAGATGGCAGTTTCCTTAGTAACCACAGGT
TGTGTCACCAGAGTAACTACATGGTAGAAAATGAAAAGATTCAACCTTTTCATTAATATACATTTAAC
AATTTTTTAAAAAACGATTTTGCTCATTCTCATGC,gtacctccaacatcaaggaagatggcatttct
agtttggagatggcagtttccttagtaaccacaggttgtgtcaccagagtaacagtctgagtaggagc
taaaatattttgggtttttgcaaaaaggaaaaaagaagaaaaagaaaaattagaaacacaagctaaag
agccaatttcaataacaataagtcaaatttaattgaagagtaacaatttgagccaaactcttattcat
gacattatatatctttttctaacaatgtggatactttgtttagcaatacatggtagaaaatgaaaaga
ttcaaccttttcattaatatacatttaacaattttttaaaaaacgattttgctcattctcatgc -an
Del,Ref --exclude_bp_from_left 30 --exclude_bp_from_right 30 --
max_paired_end_reads_overlap 368 -amas 40 --ignore_substitutions --
trim_sequences --trimmomatic_options_string
"ILLUMINACLIP:adapter.fa:0:90:10:0:true LEADING:25 TRAILING:25" -qwc
60-100,65-300\

done
```

Figure S14. Scripts used for characterizing genome editing outcomes in myoblasts cells exposed to different experimental set up comprising the use of AdVP.SpC9, AdVP.SaC9 and AdV.SpC9::SaC9 in combination with gSa^{EX51} and gSp^{IN50} or AdV.SpC9::SaC9.dgRNA$^{\Delta 51}$. The scripts are used for CRISPResso2 analysis via the Docker containerization systems. The results of these analysis are presented in Figure 2-3-4.

AL356154

```
#!/bin/bash
for fl in *_R1_001.fastq.gz
do

  SAMPLE=$(echo ${fl} | sed "s/_R1_\001\.fastq\.gz//")
echo ${SAMPLE}_R1_001.fastq.gz ${SAMPLE}_R2_001.fastq.gz
CRISPResso --fastq_r1 ${SAMPLE}_R1_001.fastq.gz --fastq_r2
${SAMPLE}_R2_001.fastq.gz --amplicon_seq
AATGGTTGAACTGAAGCTCAGTTCCTAAAAATCAATAGGCAAACATCACAGAGACCTCATTATTTGCT
TAGCAATACAAGGATATGTTAAGACAGGAATGATATATTTGGAGTCCACCTTTGTAAGAAAGAAAAAA
TATTTTGAATAAATTATAGATACTGAGAGACAGCATTTCCATTATGACTATGGAAATGACTCCCTGCA
--exclude_bp_from_left 10 --exclude_bp_from_right 10 -g
ATTATTTGCTTAGCAATACAAGG -w 20 --ignore_substitutions\

done
```

GABBR2

```
#!/bin/bash
for fl in *_R1_001.fastq.gz
do
  SAMPLE=$(echo ${fl} | sed "s/_R1_\001\.fastq\.gz//")
echo ${SAMPLE}_R1_001.fastq.gz ${SAMPLE}_R2_001.fastq.gz
```

```
CRISPResso --fastq_r1 ${SAMPLE}_R1_001.fastq.gz --fastq_r2
${SAMPLE}_R2_001.fastq.gz --amplicon_seq
gaattgaggctccaggagattaagaagcaaagttttcagctaatcagaggtggagctgtgatttgaac
ccaggtctctgacttcaaaatctaataagctgtttttaccataccatgtttagcaacacagggtttgg
cacaaagtagatgatcaatagatacttgctggatgaaaggatggatggatgcatgggtactgtcagca
gcaaaaaatgctaaaaatttttaacgaacaagcagaaagatcttggagcatgcctggaagcaagtcca
cagaacgg --exclude_bp_from_left 10 --exclude_bp_from_right 10 -g
ataccatgtttagcaacacaggg -w 20 --ignore_substitutions\

done
```

MYOZ3

```
#!/bin/bash
for fl in *_R1_001.fastq.gz
do

  SAMPLE=$(echo ${fl} | sed "s/_R1_\001\.fastq\.gz//")
echo ${SAMPLE}_R1_001.fastq.gz ${SAMPLE}_R2_001.fastq.gz
CRISPResso --fastq_r1 ${SAMPLE}_R1_001.fastq.gz --fastq_r2
${SAMPLE}_R2_001.fastq.gz --amplicon_seq
tagaatgtcctgcgtcctggatttggcttatcgcttcctcatggtgtggttaaacttgttcttctgtc
catttctcttgaaccctctccagttaggctttcacacccaaacactccatagaaactgctttagtga
aaattaccaatgaccttgtattgctaaatataatagtggattttcagccctcatcttccttgacacat
cagcagcacttggctctgttagtctctctatcatacctgaaatgctttcatcatttatttccaggatg
ccatcctctttagtttcttcctatgttgtgggctgc --exclude_bp_from_left 10 --
exclude_bp_from_right 10 -g ccttgtattgctaaatataatag -w 20 --
ignore_substitutions\

done
```

LAMA2

```
#!/bin/bash
for fl in *_R1_001.fastq.gz
do

  SAMPLE=$(echo ${fl} | sed "s/_R1_\001\.fastq\.gz//")
echo ${SAMPLE}_R1_001.fastq.gz ${SAMPLE}_R2_001.fastq.gz
CRISPResso --fastq_r1 ${SAMPLE}_R1_001.fastq.gz --fastq_r2
${SAMPLE}_R2_001.fastq.gz --amplicon_seq
Cccaagagtgagagtttgtcttgaggagaagtgtttaaaaggaatatataaatcaaggatgcactata
gtatcaccaaagtaacagtgtgaacttgaacaatttaacctcttagagccacgattgctccatctata
caatagaataataataataaacctacattatacaatttccggagattaaatgaggataaagagtactt
ttgaaattctaaagttccatgaaaatgttagctttgattacatatggttcagcaattgggtctc --
exclude_bp_from_left 10 --exclude_bp_from_right 10 -g
tagtatcaccaaagtaacagtgtgaac -w 20 --ignore_substitutions\

done
```

ZNF433

```
#!/bin/bash
for fl in *_R1_001.fastq.gz
do
  SAMPLE=$(echo ${fl} | sed "s/_R1_\001\.fastq\.gz//")
echo ${SAMPLE}_R1_001.fastq.gz ${SAMPLE}_R2_001.fastq.gz
```

```
CRISPResso --fastq_r1 ${SAMPLE}_R1_001.fastq.gz --fastq_r2
${SAMPLE}_R2_001.fastq.gz --amplicon_seq
cttaccacgggcattgctttagagtactcgggtgtcctccagcctgctcgaacactcattgggttagt
tccacattctccaactgttactccggtgacactttgacctgggttcaagacccacacatgtacaccac
ttgccaagaccatctcggtcatggagaccctaacccaggggcactagaggaattaaagacacacacag
agaaatatggagtgtggagtggcaaatc --exclude_bp_from_left 10 --
exclude_bp_from_right 10 -g tctccaactgttactccggtgacactt -w 20 --
ignore_substitutions\

done
```

LYPD6

```
#!/bin/bash
for fl in *_R1_001.fastq.gz
do
  SAMPLE=$(echo ${fl} | sed "s/_R1_\001\.fastq\.gz//")
echo ${SAMPLE}_R1_001.fastq.gz ${SAMPLE}_R2_001.fastq.gz
CRISPResso --fastq_r1 ${SAMPLE}_R1_001.fastq.gz --fastq_r2
${SAMPLE}_R2_001.fastq.gz --amplicon_seq
caagcatttgacgaagaaggtagttattatggaggaactggctttcttctcattgttactctggttac
acagaccatcagctacaaagcagcaggaatcagcccttataccaggagtcagacctgtcctttctcat
gtttcttcagaccccttccattctctgagctcccatatatcattctagccagtccccttttattccaa
taaagttttatgttagctagggtcagtttctgttgcttgccaccaaagaaccctgctggtttagggta
gttgagctgggatt --exclude_bp_from_left 10 --exclude_bp_from_right 10
-g cttctcattgttactctggttacacag -w 20 --ignore_substitutions\

done
```

Figure S15. Script used for assessing off-target DNA cleavage upon all-in-one AdVP transduction in wild-type myoblasts. DNA cleaving activities at the dual gRNA target sites in *DMD* intron 50 and *DMD* exon 51 and at three top-ranked candidate off-target sites for gSp^{EX51} (i.e., *AL356154, GABBR2* and *MYOZ3*) and gSa^{IN50} (i.e., *LAMA2, ZNF433* and *LYPD6*) were quantified by amplicon deep sequencing using ~50,000 paired-end reads per sample using the script here reported. The scripts are used for CRISPResso2 analysis via the Docker containerization systems. The result of this analysis is presented in Figure 4E.
